# Common Features at the Start of the Neurodegeneration Cascade

**DOI:** 10.1371/journal.pbio.1001335

**Published:** 2012-05-29

**Authors:** Rubén Hervás, Javier Oroz, Albert Galera-Prat, Oscar Goñi, Alejandro Valbuena, Andrés M. Vera, Àngel Gómez-Sicilia, Fernando Losada-Urzáiz, Vladimir N. Uversky, Margarita Menéndez, Douglas V. Laurents, Marta Bruix, Mariano Carrión-Vázquez

**Affiliations:** 1 Instituto Cajal, IC-CSIC & Centro de Investigación Biomédica en Red sobre Enfermedades Neurodegenerativas (CIBERNED), Madrid, Spain; 2 Instituto Madrileño de Estudios Avanzados en Nanociencia (IMDEA-Nanociencia), Madrid, Spain; 3 University of South Florida, College of Medicine and Byrd Alzheimer's Research Institute, Tampa, Florida, United States of America; 4 Institute for Biological Instrumentation. Russian Academy of Sciences, Pushchino, Moscow Region, Russia; 5 Instituto de Química-Física Rocasolano, IQFR-CSIC & Centro de Investigación Biomédica en Red sobre Enfermedades Respiratorias (CIBERES), Madrid, Spain; 6 Instituto de Química-Física Rocasolano, IQFR-CSIC, Madrid, Spain; University of California San Francisco/Howard Hughes Medical Institute, United States of America

## Abstract

Amyloidogenic neurodegenerative diseases are incurable conditions with high social impact that are typically caused by specific, largely disordered proteins. However, the underlying molecular mechanism remains elusive to established techniques. A favored hypothesis postulates that a critical conformational change in the monomer (an ideal therapeutic target) in these “neurotoxic proteins” triggers the pathogenic cascade. We use force spectroscopy and a novel methodology for unequivocal single-molecule identification to demonstrate a rich conformational polymorphism in the monomer of four representative neurotoxic proteins. This polymorphism strongly correlates with amyloidogenesis and neurotoxicity: it is absent in a fibrillization-incompetent mutant, favored by familial-disease mutations and diminished by a surprisingly promiscuous inhibitor of the critical monomeric β-conformational change, neurotoxicity, and neurodegeneration. Hence, we postulate that specific mechanostable conformers are the cause of these diseases, representing important new early-diagnostic and therapeutic targets. The demonstrated ability to inhibit the conformational heterogeneity of these proteins by a single pharmacological agent reveals common features in the monomer and suggests a common pathway to diagnose, prevent, halt, or reverse multiple neurodegenerative diseases.

## Abstract 2

A single-molecule study reveals that neurotoxic proteins share common structural features that may trigger neurodegeneration, thus identifying new targets for therapy and diagnosis.

## Author Summary

Neurodegenerative diseases like Alzheimer's or Parkinson's are currently incurable. They are caused by different proteins that, under certain circumstances, aggregate and become toxic as we grow older, but the molecular events underlying this process remain unclear. The lack of a well-defined structure, and the tendency of these “neurotoxic proteins” to aggregate make them difficult to study using conventional techniques. Here, we use an established single-molecule manipulation technique combined with a new protein-engineering strategy to show that all these proteins can adopt a rich collection of structures (conformers) that includes a high proportion of mechanostable conformers, which are associated with toxicity and disease. We also find that a known drug can block the formation of these mechanostable structures in different neurotoxic proteins. We suggest that the most mechanostable conformers, or their precursors, may trigger the pathogenic cascade that results in toxicity. We thus propose that these mechanostable structures are ideal targets for early diagnosis, prevention, and treatment of these fatal diseases.

## Introduction

Amyloid-related neurodegenerative diseases are incurable disorders that are currently classified as conformational diseases (a subset of the proteinopathies). These diseases are thought to be primarily caused by an unknown conformational change in specific proteins, termed “neurotoxic proteins” (NPs), which confers them a cytotoxic gain of function. This initial transition in the soluble monomer triggers a series of events that lead to the formation of small soluble oligomers and protofibrils, and ultimately the generation of highly ordered fibrillar insoluble aggregates. More than 20 of these NPs have been found to be intrinsically disordered proteins (IDPs). Despite the fact that the monomers of NPs have different primary and, when folded, tertiary structures, their oligomers and aggregates share common structural properties [1],[2], including recognition by a conformational antibody [3]. Whereas the nature of the cytotoxic species and the molecular mechanisms involved in their misfolding and aggregation remain largely unknown, there is growing evidence that conformational changes preceding aggregation are fundamental in the molecular pathogenesis of these diseases [1]. Furthermore, as the formation of monomeric misfolding intermediates is regarded as the earliest event in the pathogenic cascade, this process represents an ideal therapeutic target [4].

Remarkably, it was recently demonstrated that the key conformational change that endows amyloidogenesis occurs in the monomer, triggering aggregation and fibrillogenesis as well as cytotoxicity [5]–[7]. Thus, to understand the acquisition of toxic properties by NPs, the three-dimensional structure of this toxic monomeric conformer must be characterized. However, the propensity of monomers to aggregate in vitro hampers their structural analysis using classical high resolution techniques such as X-ray crystallography or solution nuclear magnetic resonance (NMR). Nevertheless, it was shown that some NPs are rich in α-helical [8],[9] or disordered [10],[11] segments. It must be noted that the oligomeric state of α-synuclein, the NP associated to Parkinson disease, remains controversial. Thus, while some reports validate it as a natively unfolded monomer [12], other studies have shown, interestingly, that its potential native state is a dynamic α-helical tetramer [13],[14] that undergoes unfolding/folding cycles associated to its physiological function to promote SNARE complex formation in neurons [15]. Furthermore, it was suggested that many alternative monomeric conformations may coexist in a dynamic equilibrium that progressively shifts toward conformers enriched in β-structure, which are thought to be the first toxic species [2]. However, the inherent ensemble averaging of these techniques prevents the full complexity of monomer conformational equilibria from being elucidated and the fast fluctuations involved from being resolved. Moreover, solid-state NMR and X-ray crystallography can only provide structural information about the final species (i.e., amyloid fibers [16]).

Bulk-based, theoretical, and single-molecule studies support the existence of conformational heterogeneity in intrinsically disordered NPs [1],[17],[18]. However, the co-existence of several species in solution at equilibrium, some of which may be scarcely populated, makes analyzing IDPs a particularly challenging task using established biophysical techniques [19]. By contrast, single-molecule techniques provide a unique opportunity to tackle this problem being able to reveal monomeric conformers [18]. Some of these techniques have already been used to monitor the activity and conformation of proteins with conformational plasticity [20], resolving fast fluctuations (20–300 ns) at the monomeric level [21]. Of the available techniques, nanomanipulation has two additional advantages. First, it allows the forces involved in stabilization of the intramolecular interactions to be directly measured (mechanical stability); and second, it enables the mechanical stability of a protein to be related with its conformation. In particular, atomic force microscopy (AFM)-based single-molecule force spectroscopy (SMFS, for short) shows remarkable potential to analyze the conformational equilibrium of monomers, and even to detect low-abundant conformers [18]. In principle, measuring the mechanostability of NPs using SMFS should be feasible, as the toxic gain of function of NPs parallels the acquisition of β-structure [1],[2], a secondary structure that is usually detectable and is often more mechanostable (M) than α-helical or disordered (random coil [RC]) conformations [22]. Furthermore, determining the mechanostability of NPs is particularly relevant, given their processing by chaperones and chaperone-related proteases [23], which unfold their substrates mechanically using AAA+ ATPase molecular motors [24],[25].

**Figure 1:**
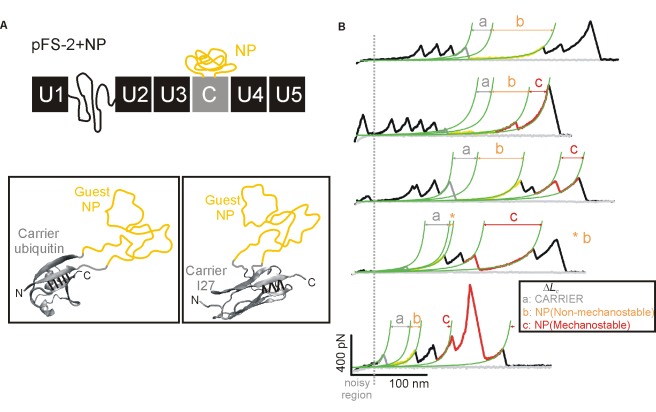
Nanomechanical analysis of NPs using the pFS-2 strategy. (A) Top: Schematic representation of the pFS-2 polyprotein [30] carrying a guest NP (in orange) mechanically protected within a carrier module (C, in gray), flanked by ubiquitin repeats (U, in black). Bottom: cartoon representations of the two carrier-guest constructions used in this study: ubiquitin (left, PDB code 1d3z) and titin I27 (right, PDB code 1tit). The images were prepared by VMD 1.8.6 [58]. The hydrogen bonds of the mechanical clamp in both carriers are indicated by black bars [51]. (B) Representative force-extension recordings of pFS-2+Sup35NM. Using this strategy, we can unambiguously resolve a variety of conformations adopted by Sup35NM, ranging from a typical NM conformation (top trace, in orange), to M conformations with different degrees of mechanical stability (in red), including hM conformers (bottom). This color code will be maintained throughout the rest of the article. On the basis of our carrier-guest design, the carrier module must always unfold completely (“a” is Δ*L*_c_ for the carrier module) before the force can access and stretch the guest NP, resulting in its unfolding (“b” and “c” represent the Δ*L*_c_ for NM and M regions of the NP, respectively). The sum of b+c corresponds to the complete unfolding of the NP. The pFS-2 vector also contains an NM region, represented as a coil (a fragment of titin N2B [30]) that can act as a spacer to avoid the noisy proximal region of the force-extension recordings, a major problem in SMFS.

Previous SMFS studies of NPs [26]–[29] have been hindered by three serious problems. Firstly, standard polyproteins were used as single-molecule markers fused to the NP, and since the NP is placed in series with the marker repeats, the SMFS recordings obtained are typically contaminated in the proximal region (Figure 1), thereby compromising the reliability of the data obtained. Secondly, the amyloidogenic behavior of the NP fused to the polyprotein was not demonstrated. Finally, there is no convincing evidence demonstrating that the selected data originated from bona fide intramolecular interactions (rather than intermolecular interactions). In the present study, these limitations have been overcome to reveal the existence of a rich conformational polymorphism in the monomer of NPs and to demonstrate its link to amyloidogenesis and neurotoxicity (Figure S1).

## Results

### Unequivocal Single-Molecule Analysis and Controls for Structure and Amyloidogenesis

To circumvent the first of the aforementioned limitations, we recently developed a novel strategy that uses a new vector to mechanically protect the NP as a guest inside a carrier protein of well-known mechanical properties (plasmid for force spectroscopy-2 [pFS-2]) (Figure 1A) [30]. Accordingly, unlike previous works [26], by means of this approach (termed the “carrier-guest” strategy) the SMFS data obtained from the NP are always read after the unfolding of the carrier, far from the proximal region to the AFM substrate, which is known to be typically noisy and thus unreliable (Figure 1B; Text S1). We used SMFS to analyze the nanomechanics of NPs expressed as fusion polyproteins in pFS-2. Using the so-called length-clamp mode of this technique, two basic parameters are directly obtained (see Text S1): the mechanical stability of the resistance barriers (*F*, measured directly by the height of the force peaks in a force-extension recording), and the length released after unfolding (increase in contour length (Δ*L*_c_) as measured by fitting the force-extension recording to the worm-like chain [WLC] model of polymer elasticity). We followed highly stringent criteria when selecting single-molecule recordings using the carrier-guest mechanical protection strategy whereby we only selected those recordings in which the Δ*L*_c_ of the carrier (alone or including a non-mechanostable fraction from the NP) was followed, although not necessarily consecutively [29], by force peaks with Δ*L*_c_ values that accounted for the total expected length released by the carrier-guest protein (Figure 1B; Text S1).

This strategy was employed to study four model NPs: three human NPs (expanded poly-glutamines -polyQ-, β-amyloid_1–42_ -Aβ42-, and α-synuclein) and a neurotoxic-like protein that has been extensively used as a model for human prions (Sup35NM, a yeast prion). These NPs are amyloidogenic IDPs [18], and they are thought to cause the most representative neurodegenerative diseases: polyQ (familial disorders such as Huntington's disease), Alzheimer's, Parkinson's, and prion diseases, respectively [1],[18]. All these NPs, including Aβ [31], are found intracellularly, and they are therefore susceptible to be stretched by the protein-processing machinery [23].

To test whether the properties of these engineered NPs and carriers are preserved in the carrier-guest proteins (the second limitation of the former SMFS studies listed above), we performed standard structural, calorimetric, and fibrillogenic experiments on the isolated carrier-guest proteins (Figures S2, S3, S4, S5, S6, S7, S8, S9; Table S1; Text S1). Whereas the conformational stability of the carrier proteins is reduced when they contain the guest NP, and in some preparations some molecules even lose their folded tertiary structure, the carriers essentially maintain their structure and mechanical properties (Table S2), which enables the unambiguous observation of the conformational polymorphism of the monomeric NPs. Furthermore, regarding the possible presence of spurious interactions, in these studies we found no evidence of contacts or other interactions between the carrier and guest proteins beyond the covalent linkage of the polypeptide chain. This rules out the possibility of artifactual contacts, and hence a possible gain of structure, induced in the NPs when nested inside the carriers or the other way around.

To the best of our knowledge, the pFS-2 vector is the first strategy capable of successfully avoiding the contamination of SMFS data with non-specific interactions from the proximal region of the AFM, which is particularly useful for studying IDPs [30]. Indeed, this approach guarantees that the selected data originate from the stretching of single NP molecules (Figure 1). However, in principle, the stretching of a single NP monomer does not exclude possible interactions of the NP with the carrier, the AFM elements (substrate or tip) or other surrounding NPs (intermolecular interactions from oligomers and/or fibers). Such potential interactions (the third limitation of those SMFS studies listed above) must be ruled out before force events can be unequivocally attributed to intramolecular interactions in the NP monomers. By using specific controls, we provide compelling evidence that in our experimental conditions, the stringently selected SMFS data (we sampled about 100 molecules of each NP with each set of recordings resulting from 50,000–100,000 pulling attempts) exclusively arose from monomeric NPs and, therefore, represent bona fide intramolecular interactions (Text S1).

Finally, by restricting the degrees of freedom of the hosted NPs, the pFS-2 strategy has the additional advantage of dramatically slowing down fibrillogenesis (Figure S6D; Text S1), thereby minimizing the formation of aggregates, during the nanomechanical analysis of the monomeric NPs.

### Conformational Polymorphism of NPs

**Figure 2:**
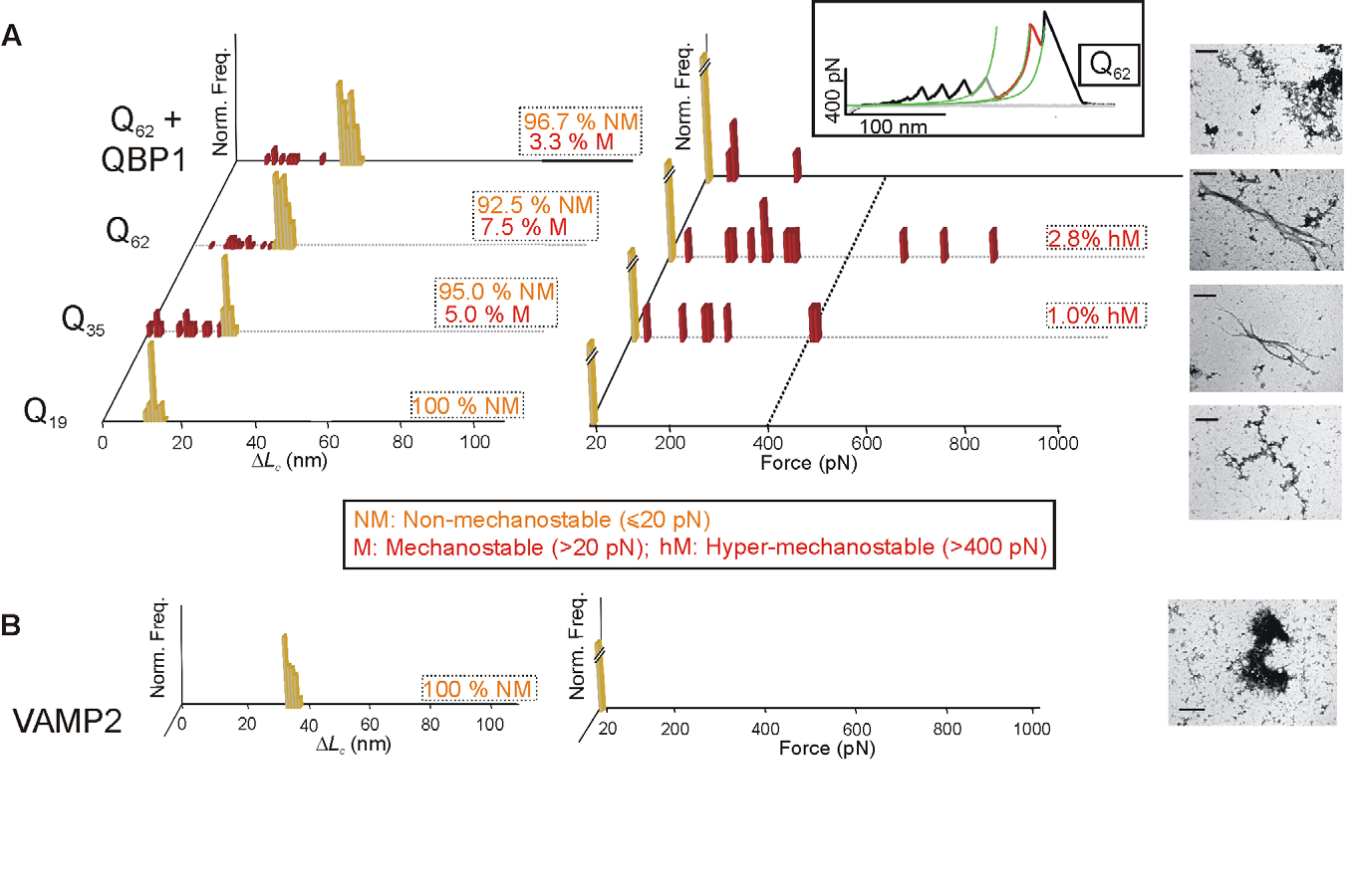
Nanomechanical analysis of polyQ tracts and a non-amyloidogenic IDP (VAMP2). (A) Δ*L*_c_ (left) and *F* (right) histograms for pFS-2 polyproteins carrying polyQ tracts. The sub-threshold Q_19_ (front row) only shows NM conformers (orange bars; represented in the force histograms below the force sensitivity of our AFM: *F*∼20 pN). Familial-disease mutations of this protein (expanded polyQs: Q_35_ and Q_62_) exhibit conformational polymorphism that ranges from NM conformers to M conformers (red bars), the latter class including some hM conformers (*F*≥400 pN, i.e., likely toxic conformers according to our hypothesis, see text). It should be noted that the longer the polyQ tract, the greater the conformational polymorphism and the more hM conformers found. The inhibitor QBP1 (20 µM [42]) reduces this polymorphism and abolishes the hM conformers. The inset shows an example of a hM conformer of Q_62_. Note that the hM class of conformers is a subset of the M set so that the percentage of hM conformers (respect to the total number of molecules sampled, *n*) is included into that of M (this also applies to the remainder main figures of this work as well as to Table 1). TEM images of the amyloid fibers formed by the corresponding carrier-guest proteins (not the whole pFS polyprotein) are shown on the right, highlighting the relationship between hM conformers and amyloidosis. From bottom to top, the scale bars correspond to 0.6, 0.3, 0.3, and 0.6 µm, respectively. (B) SMFS analysis of pFS-2+VAMP2. This non-amyloidogenic IDP [35] does not show conformational polymorphism. The scale bar from the TEM image on the right corresponds to 0.6 µm. Only data from the guest protein, and not from the carrier, are plotted in the histograms presented in this figure and Figures 3–5.

**Figure 3 pbio-1001335-g003:**
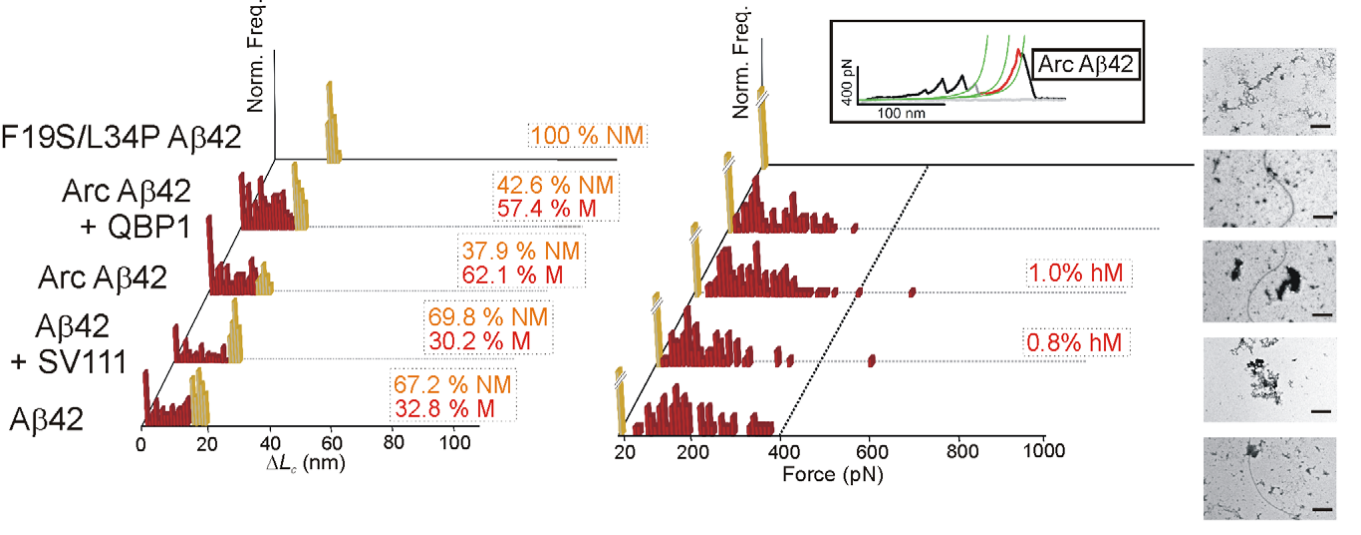
Nanomechanical analysis of Aβ42. Δ*L*_c_ (left) and *F* (right) histograms of pFS-2 polyproteins carrying Aβ42. The wt protein (first row) shows broad polymorphism ranging from NM conformers (orange bars) to M conformers (red bars). No hM conformers were found in this protein. Incubation of Aβ42 with and without SV111 (100 µM [38]) yielded similar results, strongly indicating that our SMFS data reflect unfolding events originating from different conformations adopted by the monomeric forms of NPs (as opposed to intermolecular interactions of the oligomeric species). Arc Aβ42 (E22G) familial-disease mutation increases the number of M and hM conformers when compared to the wt protein. Incubation with QBP1 peptide (20 µM [42]) does not impair the formation of M conformers in Arc Aβ42. For F19S/L34P Aβ42 only NM conformations were observed despite the larger sample size (Table 1). TEM images of the amyloid fibers formed by the corresponding proteins are shown on the right. From bottom to top, the scale bars correspond to 0.6, 0.6, 0.45, 0.35, and 0.9 µm, respectively. An example of hM conformer of Arc Aβ42 is shown in the inset.

PolyQ diseases are unique among amyloidogenic neurodegenerative diseases in that they are both genetically determined and their NPs, polyQ expansions, have pathological thresholds (21–30 and 36–40 glutamine residues in spinocerebellar ataxia type 6 [SCA6] and Huntington disease, respectively). Interestingly, there is a positive correlation between the number of glutamine repeats and the severity of the disease, the age of onset, and the oligomerization kinetics [4],[32]. Thus, in the simplest hypothesis, the disease determinants are expected to correlate with the number of glutamine residues. Using SMFS we looked for possible mechanical differences in the monomeric polyQ tracts of three lengths that are considered to be sub-, near- (except for SCA6) and super-threshold for triggering polyQ diseases: Q_19_, Q_35_, and Q_62_ (Figure 2A). We found that the subthreshold tract showed no force peaks; we term these conformers “non-mechanostable” (NM) (i.e., there were no detectable SMFS conformations; the force detection limit of SMFS was taken as twice the force error, 20 pN, at a pulling speed of 0.4 nm/ms) (Figure 2A, orange bars; this representation is followed also in Figures 3–5; Text S1). Similar mechanical behavior was observed for non-amyloidogenic/non-neurotoxic IDPs [33],[34], including VAMP2 (Figure 2B) [35]. Accordingly, neither Q_19_ nor VAMP2 constructs produced amyloidogenesis (Figures 2, S2C, S2D, S3C, and S3D).

**Figure 4 pbio-1001335-g004:**
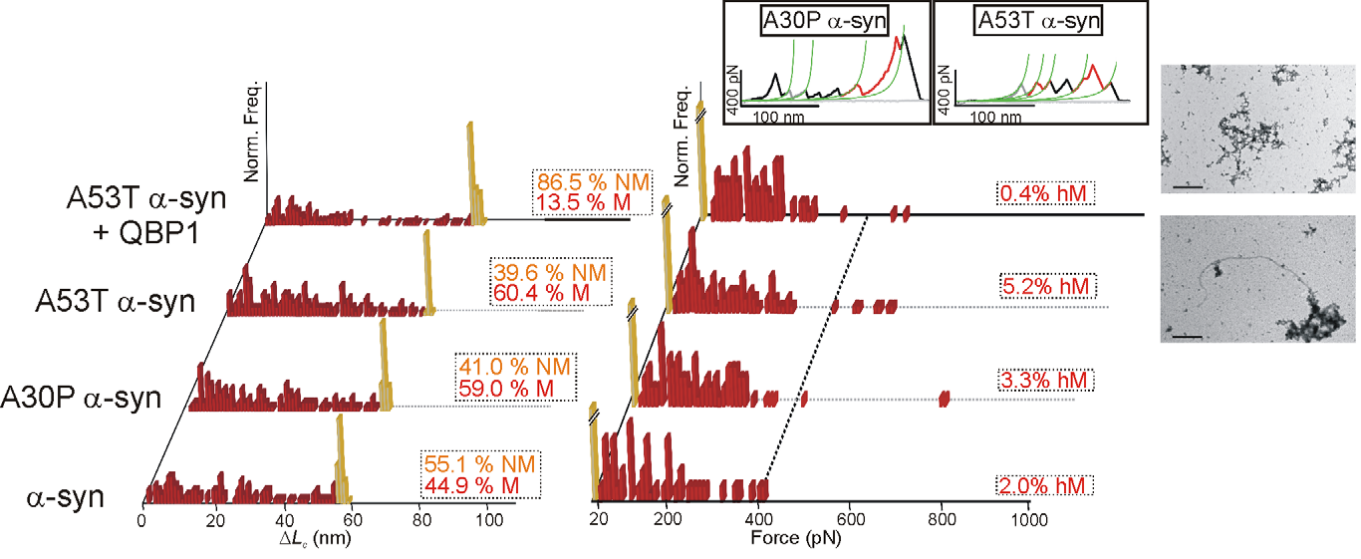
Nanomechanical analysis of α-synuclein. Δ*L*_c_ (left) and *F* (right) histograms of pFS-2 polyproteins carrying α-synuclein. The wt protein (first row) exhibits a wide-range polymorphism ranging from NM conformers (orange bars) to M conformers (red bars), including some hM conformers. Familial-disease mutations A30P and A53T increase the number of M and hM conformers of α-synuclein when compared to the wt. Treatment with QBP1 peptide (20 µM [42]) reduces the formation of M and hM conformers in A53T α-synuclein. TEM images of the amyloid fibers formed by ubi+A53T α-synuclein are shown on the right in which amyloid fibers are clearly not formed in the presence of QBP1 (top image). From bottom to top, the scale bars correspond to 0.45 and 0.6 µm, respectively. Examples of hM conformers of A30P and A53T α-synuclein are shown in the inset.

**Figure 5:**
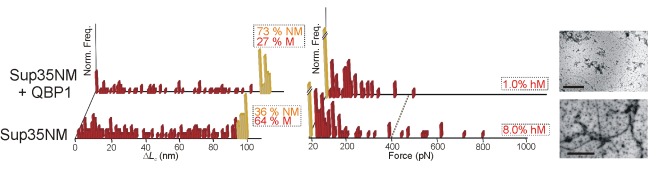
Nanomechanical analysis of Sup35NM. Δ*L*_c_ (left) and *F* (right) histograms of pFS-2 polyproteins carrying Sup35NM. Like the wt proteins in Figures 3 and 4, Sup35NM (first row) shows a broad polymorphism, including rare hM conformers. Treatment with QBP1 peptide (20 µM [42], back row) decreases the formation of M and hM conformers, as seen for both polyQ and α-synuclein (Q_62_ and A53T, Figures 2 and 4). TEM images of the amyloid fibers formed by I27+Sup35NM are shown on the right. Addition of QBP1 clearly reduces the formation of amyloid fibers. The scale bars correspond to 0.6 (top) and 0.4 µm (bottom).

By contrast, Q_35_, which showed moderate amyloidogenesis (Figure S2C and S2D), exhibited not only numerous NM conformations (*F*≤20 pN) but also several force peaks with a variety of Δ*L*_c_ values; we call these conformers “mechanostable” (M) (*F*>20 pN) (Figure 2A, red bars). Δ*L*_c_ and *F* were found to be uncorrelated (Figure S10A) as recently reported for the NP tau [29]. Interestingly, this incipient conformational polymorphism included conformers with extremely high mechanical stability (*F*≥400 pN). Given that the mechanical stability (upon N-C stretching) of eukaryotic proteins studied to date is below 400 pN [22], we have operationally defined an additional subset in the latter class based on an arbitrary force value of 400 pN. This subset of M conformers that accounts for events with *F*≥400 pN (which are rare) is termed hyper-mechanostable (hM).

Based on our current knowledge of NPs [1] and recent studies of polyQ tracts [5], it is likely that NM and M conformers correspond to RC and β-stranded structures, respectively (see Text S1), as has been previously assumed [26],[27]. Together, these results indicate that polyQ tracts of 35 glutamines can experience a structural transition that permits conformational fluctuations. Significantly, Q_62_ exhibited even greater conformational polymorphism that also included a higher number of hM conformers (Figure 2A). Thus, expanded polyQ tracts (Q_35_ and Q_62_) allow the appearance of conformations that can be detected by SMFS; these mechanical conformations include hM conformers. This polymorphism (estimated as the frequency of M conformers, including hM ones) is positively correlated with the polyQ length, which in turn correlates with amyloidogenesis. Interestingly, a structural correlation with the pathological threshold for polyQs has been previously reported [5].

The SMFS analysis of the three NPs used as models of sporadic neurodegenerative diseases (Aβ42, α-synuclein, and Sup35NM) [1],[2] showed that the wild-type (wt) form of each protein exhibited a rich conformational polymorphism, which also included rare hM conformers (except for Aβ42) (Figures 3–5). The differences in the occurrences of stable conformers in NPs relative to non-amyloidogenic IDPs (VAMP2, Q_19_, and F19S/L34P Aβ42, see below) are statistically significant (Table 1).

**Table 1:** Summary of the nanomechanical analysis of NPs.

Protein			*n*	NM (%)	M (%)	hM (%)
PolyQ	Q_19_		111	100	0	0
	Q_35_		100	95.0	5.0 (4)	1
	Q_62_	−QBP1	107	92.5	7.5 (5)	2.8
		+QBP1	124	96.7	3.3 (3)	0
VAMP2	I27-VAMP2		188	100	0	0
	Ubi-VAMP2		234	100	0	0
Aβ	Aβ42	−SV111	116	67.2	32.8 (9)	0
		+SV111	128	69.8	30.2 (8)	0.8
	Arc Aβ42	−QBP1	102	37.9	62.1 (10)	1
		+QBP1	108	42.6	57.4 (9)	0
	F19S/L34P Aβ42		396	100	0	0
α-syn	wt		98	55.1	44.9 (10)	2±1
	A30P		92	41.0	59.0 (10)	3.3
	A53T	−QBP1	96	39.6	60.4 (10)	5.2±1.0
		+QBP1	259	86.5	13.5 (4)	0.4
Sup35NM		−QBP1	100	36.0	64.0 (10)	8
		+QBP1	100	73.0	27.0 (9)	1

SMFS experiments were performed on pFS-2 polyprotein constructs, although the data reported only refer to the guest NPs. The calculation of the associated experimental errors is described in Text S1 and they are only indicated when they are different to zero. Due to the small number of events, the frequency of hM conformers in the NPs (a subset of the M set, operationally defined with a high *F* cut off) is not statistically significant. However it correlates very well with the %M. Furthermore, statistical analysis shows that, with the exception of polyQ tracts (for which the number of M, events is too low), the differences in the %M events for NP guests are statistically significant for the following pairs: NP and non-NP, NP and familial NP, NP (except for Arc Aβ42) and NP+QBP1. These differences are not statistically significant for the following pairs: Aβ42 and Aβ42+SV111, Arc Aβ42 and Arc Aβ42+QBP1, and non NPs. The numbers in parenthesis are half of the 95% confidence interval for the %M (or NM, modeled as a Bernoulli distribution) while the numbers after ± are the SMFS experimental errors. Note that the % numbers here are the estimation for the population while the % numbers in Figures 2–5 correspond to the raw samples.

*n*, sample size. NM, no force peaks detected: *F*≤20 pN; M, at least one force peak: *F*>20 pN; hM, subset of M conformers with at least one force peak with *F*≥400 pN.

In the wt form of the four model NPs, the frequency of M conformers ranged from 0% in Q_19_ (Q_21_ is the minimum pathological threshold detected in polyQ diseases, specifically in SCA6 [2]) to 64% in Sup35NM, which for each NP was positively correlated with amyloid fiber density (Figures 2–5, S2D, S3D, S4D, S5D, S6D; Table S1). The frequency of hM conformers for wt NPs ranged from 0% to 8% (Figures 2–5). These rare events are associated with extremely high rupture forces (up to ∼800 pN), greater than those reported for the most mechanostable proteins described to date (i.e., bacterial cohesin modules [36]) and just below the force range required to break a covalent bond [37]. Table 1 summarizes the frequency for each conformer type. Frequently, more than one force peak was found for each NP molecule (Figure S11), suggesting the presence of more than one structured region in the M conformers.

It should be noted that the number (and thus the frequency) of M conformers may in reality be greater than that estimated due to the existence of additional SMFS curves that could correspond to incomplete recordings, i.e., compatible with the unfolding of a carrier bearing a mechanostable element that was not fully observed probably due to the premature detachment of the molecule. Although mechanostability is a stochastic property, these premature detachment events should often be lower than the forces needed to unravel the undetected NP (putative M conformers). A subset of these recordings contained putative hM events (Figure S12). Although these putative events were not included in the sample size, *n*, it is interesting to note that there is a good correlation between the putative and detected hM events among the different NPs and conditions tested (Table S3).

Taken together, SMFS analysis revealed that whereas VAMP2, a non-neurotoxic IDP, always shows a low (undetectable) resistance to mechanical unfolding, all the NPs studied behaved similarly, displaying a high degree of mechanical conformational polymorphism that often included a few hM conformers. We postulate that this behavior is associated with amyloidogenesis and neurotoxicity, which, in agreement with previous results [5], strongly suggests that a critical conformational change in the monomer leads to the generation of the first toxic NP species. Hence, we have tested this working hypothesis by studying four conditions in which the disease and/or amyloidogenesis are affected in two ways: diminished with a peptide that blocks the oligomerization process, an aggregation-defective mutant, or a peptide that inhibits the β-conformational change; and intensified by familial-disease mutations. According to our hypothesis, in these conditions the conformational polymorphism (and hM frequency) should be unaltered, abolished, decreased, or increased, respectively.

### Oligomerization Inhibition Does Not Alter Polymorphism

We first studied the effect of blocking oligomerization on the conformational polymorphism of monomeric NPs. Specifically, we used the SV111 peptide, which inhibits Aβ42 oligomerization and fibrillogenesis by binding to a specific β-hairpin structure [38], but should not affect the conformational fluctuations of the unbound monomers. Notably, incubation of Aβ42 with SV111 (100 µM [38]) yielded SMFS results similar to those from the untreated Aβ42, including the number of events per molecule (Figures 3 and S11; Table 1), whereas fibrillogenesis was inhibited (Figures 3 and S4D; Table S1). Since this peptide effectively inhibits oligomerization, this experiment also served as a control to rule out a significant contribution of intermolecular interactions to our SMFS data, strongly suggesting that the behavior observed originated from bona fide intramolecular interactions within the NP monomer (Text S1).

### Fibrillogenesis-Defective Mutant Lacks Polymorphism

We then analyzed a double mutant of Aβ42 known to suppress fibrillogenesis (F19S/L34P) [39]. As expected, the results obtained (Figures 3 and S4) were similar to those obtained with Q_19_ or VAMP2 (Figures 2, S2, and S3). This experiment also served as a control to exclude potential interactions between the NPs and the AFM elements as a source of the M events (Text S1).

### Familial-Disease Mutations Increase Polymorphism

We next studied the effect of additional representative familial neurodegenerative disease mutations on the conformational polymorphism, using dominant forms responsible for the early onset of the disease and for the acceleration of oligomerization kinetics [1],[2]. In addition to Q_35_ and Q_62_, we also studied the arctic (Arc: E22G) mutation in Aβ42 that has been implicated in familial Alzheimer's disease, as well as the A30P and A53T mutations in α-synuclein that are involved in familial Parkinsonism [1],[2]. Like the results obtained with Q_35_ and Q_62_, we found an increase in the frequency of M conformers, including hM ones, for all the mutants analyzed (Figures 3 and 4; Table 1). Similarly, the number of mechanostable regions (i.e., force peaks) per molecule increased for each NP mutant studied (Figure S11). Finally, two of the familial-disease mutations also increased the number of putative hM recordings (Q_62_ in polyQ tracts and A53T in α-synuclein) (Figure S12). Taken together, these results indicate that familial-disease mutations increase the propensity to form M conformers, including hM forms, an effect that is correlated with aggregation and fibrillogenesis, at least for polyQ tracts. This increased conformational polymorphism may also explain the dominant character of these NP mutations.

### A Promiscuous β-Sheet Blocker Decreases Polymorphism

We finally analyzed how the conformational polymorphism of NPs was affected by PolyQ-binding peptide 1 (QBP1), an amyloidosis inhibitor known to block the critical β-conformational change of expanded polyQs at the monomer level. QBP1 binds RC conformers [5],[40], suppressing oligomerization, amyloidogenesis, cytotoxicity [5], and neurodegeneration [41]. Specifically, we used the minimal active core of this peptide (QBP1-M8) [42]. Incubation of Q_62_ with QBP1 completely abolished the formation of fibers (Figures 2 and S2; Table S1), and effectively reduced both the frequency of detected and putative M conformer formation, including hM forms (Figures 2, S12B; Tables 1 and S3). The number of mechanostable events per molecule (Figure S11A) was also reduced.

In what was originally intended as a negative control experiment, we also tested the effect of this peptide on the other three NPs, selecting two well-characterized familial-disease mutations (Arc Aβ42 [Figure 3] and A53T α-synuclein [Figure 4]) and the yeast prion Sup35NM (Figure 5). Surprisingly, QBP1 strongly diminished the formation of M conformers (detected and putative) of both A53T α-synuclein and Sup35NM (Figures 4, 5, and S12; Table S3). The subset of hM conformers (detected and putative) showed a significant decrease as well (Figure S12; Table S3). Aggregation and fibrillization were also reduced or suppressed by QBP1 in these two NPs (Table S1). It should be noted that the effect of the peptide on Sup35NM was incomplete as some fibers were still formed (Figures 5 and S6D), while its effect on A53T α-synuclein was not as significant, given the small number of fibers formed by this mutant in the absence of the inhibitor (Figures 4 and S5D). Interestingly, in the specific conditions used in our experiments, QBP1 appeared to have no significant effect on Arc Aβ42, either on the conformational polymorphism or its aggregation and fibrillization capabilities (Figures 3, S4C, and S4D; Tables 1 and S1). Furthermore, the frequency of hM events in the untreated Arc Aβ42 was so low (the lowest of the four NPs) that any apparent effect could not be considered significant. To rule out any effect of QBP1 on Aβ42, higher concentrations of QBP1 (from 100 µM to 1 mM) were tested on the isolated Aβ42 and no effect in fiber formation was observed (Figure S4D).

These results point to the existence of common characteristics at the monomer level in at least three of the NPs studied (expanded polyQs, α-synuclein, and Sup35NM), which appear to be recognized by QBP1. In spite of the similarities found for all the NPs studied, the inability of QBP1 to recognize Aβ42 indicates that there may be some variation in nascent amyloidogenesis. It should also be noted that structural characterization as well as aggregation and fibrillogenesis experiments (Figures S2, S5, and S6) suggest that the inhibitory effect of QBP1 is stronger on expanded polyQ tracts than on A53T α-synuclein or Sup35NM, while it seems not to affect Arc Aβ42 (at the same peptide concentration) (Figure S4), in accordance to its mentioned effect on the conformational polymorphism. Given that QBP1 blocks the formation of β-structures [5], it is likely that at least some M conformers contain such β-structures, as discussed below. These results extend previous observations of common molecular mechanisms in both oligomerization and fibrillogenesis on the basis of the recognition of similar oligomeric structures in all NPs tested by the A11 conformational antibody [3] and in the existence of a similar though somewhat variable cross-β spine structure discovered in these fibers [16],[43].

Thus, our results point to the existence of additional common molecular mechanisms upstream in amyloidogenesis acting in the initial stages of this process at the monomer level.

## Discussion

Single-molecule techniques are ideal to analyze the conformational polymorphism of NPs and SMFS in particular is a highly promising technique that has already been used to this end [26]–[29]. However, the disordered nature of NPs and their tendency to oligomerize pose significant challenges to this kind of analysis. We have successfully developed a strategy to unequivocally analyze at the single-molecule level the conformational polymorphism of IDPs, and in particular NPs. Accordingly, we have sampled the conformational space with about 100 molecules from each of the four most representative NPs and their variants: polyQ tracts, Aβ42, α-synuclein and Sup35NM (see Table 1). We demonstrate that proteins (neurotoxic or not) that do not form amyloid fibers (i.e., non-pathological polyQ tracts such as Q_19_, VAMP2, and the F19S/L34P Aβ42 mutant) show no mechanical conformational polymorphism, as monitored by SMFS. By contrast, proteins with propensity to form amyloid fibers (expanded polyQs, Sup35NM, and wt and familial-disease mutations for Aβ42 and α-synuclein) exhibit a rich conformational polymorphism that includes rare hM conformers.

As mentioned, it is likely that NM and M conformers correspond to RC and β-stranded structures, respectively. In the case of prions, conformers separated by a high-energy barrier and stabilized by strong non-covalent forces (presumably high-density hydrogen bonding), have recently been proposed to account for the heritability of conformations [44]. It is tempting to speculate that these structures may contain β-strands in a shear (rather than zipper) configuration, as this is the most mechanostable structure observed experimentally to date [36],[45]. These hM conformers were already suggested in previous SMFS studies of polyQs [28], although the authors assumed they involved collapsed rather than β-stranded structures, even for non-pathological tracts. It is important to emphasize that our SMFS results correlate closely with our structural and aggregation/fibrillogenesis controls (Table S1). However, our results are not directly comparable to those of previous studies, which did not include unequivocal single-molecule markers, structural controls, or controls for intermolecular interactions. Thus, while our findings are in line with previous results from tau [29], except that these authors did not report NM conformers, they diverge significantly from those reported for α-synuclein [26],[27], possibly owing to the larger sample size used for tau as compared with α-synuclein (214–453 versus 34–63, respectively).

Neurotoxic and neurotoxic-like proteins involved in sporadic neurodegenerative diseases (Aβ42, α-synuclein, and Sup35NM) [1],[2] exist as a broad ensemble of NM and M conformers, including a few hM forms. In contrast, Q_19_ (which fails to trigger disease) does not show M conformers. These findings and previous observations [41] strengthen the connection between M conformers, amyloidogenesis, and disease. Indeed, mutations that induce an early disease onset and accelerate amyloidogenesis [1],[2] increase the frequency of M (and hM) conformers. Conversely, treatment with the QBP1 inhibitor peptide strongly reduced the frequency of M (and hM) conformers in A53T α-synuclein and Sup35NM. Intriguingly, this treatment did not affect the conformational polymorphism of Arc Aβ42. Moreover, incubation of Aβ42 with SV111 yielded a similar conformational polymorphism as for the untreated Aβ42, although it did inhibit aggregation and fibrillogenesis, in agreement with previous results [38].

Overall, we observed an excellent correlation between the propensity to develop a neurodegenerative disease, previously demonstrated by others [1],[2], and conformational polymorphism, including hM conformers, of the relevant NPs. We show that this polymorphism can be altered in both directions, towards more “structured” conformers by pathological mutations or towards the NM conformation, by the QBP1 inhibitor and by non-fibrillogenic mutations. The dramatic reduction of the frequency of M conformers, including hM forms, produced by QBP1 suggests that hM conformers and/or their precursors from the M pool are good candidates to represent the primary cause of neurodegenerative diseases (i.e., the hypothetical initial misfolded intermediate). Since QBP1 inhibits the formation of these conformers and exerts anti-neurodegenerative effect [41], we conclude that hM conformers, and/or their precursors, are likely on-pathway intermediates in amyloidogenesis and/or cytotoxicity. The discovery of these common features offers hope that a single therapeutic agent may be able to prevent, halt, or reverse the development of many neurodegenerative diseases arising from these different proteins.

Prior to our work, it was known that common molecular mechanisms existed in the later stages of amyloidogenesis, including oligomerization [3] and amyloid fiber formation [43]. Also, prion-like behavior has been recently shown for polyQ tracts, Aβ and α-synuclein [46]. Our identification of a common pattern of conformational propensities among the monomers of the four most representative NPs further extends this unifying view to the earliest stages of the amyloidogenesis process. Furthermore, the inhibition of fibrillogenesis by the same peptide in three out of those four NPs suggest that amyloidogenesis shows common molecular features from the very beginning of the process, although some diversity is also likely [16].

In general, our results strongly support the conformational change hypothesis as the mechanism to explain the initiation of amyloidogenesis [47], wherein amyloidogenesis is thought to be preceded and triggered by a conformational change in the monomer precursor [4]. In the particular case of polyQs (the clearest-cut system), the nature of the conformational changes that occur during polyQ amyloidogenesis have remained controversial [2],[4],[48]. In fact, other models for polyQ diseases have received greater attention in the literature, such as the “polymerization hypothesis” (i.e., the conformational change is induced upon monomer polymerization into fibers) or recruitment mechanisms like the “linear lattice” model [2],[48]. Our data strongly suggest that it is the monomer that undergoes the critical conformational change that results in the generation of the first toxic species [5]. Furthermore, our data provide strong evidence supporting the so-called “structural threshold hypothesis,” which states that above the pathological threshold polyQ tracts undergo a structural transition from RC to β-sheet structure [49] and indicating that it occurs in the monomer. Our single-molecule approach also provides an explanation for the failure to detect this change using NMR spectroscopy in a previous study [50]. M conformers represent just 7.5% or less of species, a fraction that is difficult to detect even if all these conformers were from a unique species (which seems not to be the case according to our Δ*L*_c_ data). Finally, we note that in the aforementioned study the Glutathione S-transferase-Q_n_ (GST-Q_n_) fusion proteins used did not show evidence of aggregation.

We have adopted a new strategy to unequivocally identify and quantify the conformational polymorphism of NPs and their pathological and non-pathological variants. This approach permits—for the first time to our knowledge—rare hM conformers to be unequivocally detected. We postulate that these conformers, or their precursors, may mediate the primary event in the pathological cascade leading to amyloid fiber formation and concomitant neurodegenerative disease. On the basis of these findings, we propose the self-explanatory term “proteinoscleroses” to collectively refer to amyloid-related neurodegenerative diseases. In this context, hM conformers (or their earliest precursors, if we assume a sequential pathway) constitute ideal pharmacological targets and biomarkers of the propensity to develop such diseases. Moreover, the conformational inhibitor QBP1 appears to target the pathway that leads to hM conformer formation in expanded polyQs, α-synuclein, and Sup35NM prion.

Whereas there is evidence for rapid interconversion of conformers in NPs [21], we propose that once formed, the hM conformers are likely to be kinetically trapped (because of the high energy unfolding barriers) and might mechanically jam the unfoldases of the protein-processing machinery of the cell as postulated previously [51]. Such an effect would slow down their processing, which would unbalance the cell proteostasis by increasing the cytosolic concentration of hM conformers. This imbalance could affect distinct cellular processes and ultimately, it may lead to the formation of oligomers and amyloid fibers (Figure S1). There are three findings that support this hypothesis: (1) amyloid-related neurodegenerative diseases have a late onset, developing as aging impairs the efficiency of the protein-processing machinery [52],[53]; (2) the proteasome is transiently impaired in vivo by the expression of the N-terminus of pathologic huntingtin [54]; and (3) the AAA+ ATPases from the degradative machines have recently been shown to unfold their substrates mechanically using relatively low forces (with a pulling geometry apparently similar to that of SMFS experiments and a low calculated loading rate of 0.15 pN/s) [24],[25]. Alternatively, toxic NP conformers may sequester other IDPs involved in essential cell functions [55] or affect the normal lysosomal autophagy process [56]. It should also be noted that the causal relationships between misfolding and oxidative stress remain a matter of debate [57]. In spite of this, it must be noted that, alternatively, hM conformers may represent dead-end species, i.e., by-products, off-pathway of amyloidogenesis and cytotoxicity; in this case the toxic species would be part of the remaining species of the M pool.

In conclusion, our findings show a clear correlation in all NPs studied between conformational polymorphism (including the presence of hM conformers) and neurotoxicity. They strongly indicate that hM events represent highly collapsed or structured conformers in the fibrillization pathway and that these conformers, or their precursors, may be the primary cause of amyloid-related neurodegenerative diseases. Our approach opens the door to understanding the molecular mechanism of amyloidogenesis and developing a single therapeutic strategy that may treat distinct neurodegenerative diseases using a single drug. Our novel approach also offers a new means of studying other proteins involved in conformational diseases and should allow the dissection (by using the appropriate conditions) of the toxicity and fibrillogenic pathways involved in amyloidogenesis.

## Methods

Polyprotein engineering was performed using a new vector recently described by our group (pFS-2) (Table S4) [30]. Detailed methods on polyprotein sample preparation, AFM-SMFS, circular dichroism (CD), differential scanning calorimetry (DSC), NMR, turbidimetry, Congo Red binding assay, transmission electron microscopy (TEM), and imaging AFM experiments can be found in Text S1.

## Supporting Information

Figure S1 **Mechanical hypothesis of the primary cause for amyloid-related neurodegenerative diseases.** The use of the newly developed pFS-2 vector [30] has allowed us to observe a high degree of conformational polymorphism in different NPs, comprising conformations with no detectable mechanical stability (NM conformers, that exhibit no force peaks in SMFS) and M conformations (with at least one force peak), the latter including the subset of hM conformers (with at least one force peak with *F*≥400 pN). As discussed in the main text, it is reasonable to assume that both M and hM conformers are β-structured. These different conformers are thought to be in a highly dynamic equilibrium [21]; however, once formed the hM conformers may be kinetically trapped by a high unfolding barrier. We tentatively propose a plausible mechanism of cytotoxicity in which hM conformers mechanically block the cell's protein-processing machinery leading to an increase in their cytosolic concentration and affecting multiple cellular processes, which ultimately results in the formation of oligomers and amyloid fibers. These hM conformers may originate sequentially from less toxic intermediate β-structured conformations (on-pathway) or they may trigger the disease through a pathway other than that proposed here. Alternatively, hM conformers may represent kinetically trapped by-products of the amyloidogenic/cytotoxic pathway. The steps blocked by the two inhibitor peptides used in this study are indicated: QBP1 blocks the conformational change and displaces the putative RC/β-structure equilibrium towards the RC conformer (thereby interrupting the pathogenic cascade in its earliest stages), while SV111 inhibits the oligomerization of the β-hairpin monomers of Aβ42 [38].
(TIF)

Figure S2 **Controls for polyQ tract structure and aggregation in the carrier protein.** (A) Far-UV CD spectra (left) and estimated increase in β-structure content (Δ%β, relative to day zero) over the incubation period at 37°C (right). The features of the CD spectra indicate that the Q_19_ and Q_35_ tracts fused to the I27 carrier remain disordered ([θ] = molar ellipticity per residue). In addition, QBP1 appears to reduce the increase in the content of β-structure in I27+Q_35_. (B) ^1^H NMR. Left, complete 1D spectra; right, zoom of HN region where representative resonances of the I27 moiety are labeled [59]. The spectrum of I27-multicloning site (MCS, top) is very similar to that of the fully folded wt protein [59], and it exhibits the extensive chemical shift dispersion typical of structured proteins. In the constructs containing Q_19_ (middle, red) or Q_35_ (bottom, green) the I27-MCS spectrum is retained. Since the ^1^H chemical shift is exquisitely sensitive to the local environment, these data are unequivocal evidence that the native backbone structure is maintained when the carrier hosts the MCS, Q_19_, or Q_35_. Rather sharp peaks appear in the variants containing Q_19_ or Q_35_. These signals can be assigned to glutamine residue resonances: backbone HN, 8.4 ppm; sidechain H_2_N, 7.50 and 6.84; Hα, 4.3; Hγ, 2.3; Hβ, 2.1; and Hβ′, 2.0 ppm. The sharpness of these signals and the similarity of the chemical shift values to the reference values for glutamine in an RC conformation [60] indicate that these polyQ segments are disordered when grafted in I27. CD and NMR experiments were not carried out for I27+Q_62_ because of the difficulty in producing the protein in the required concentration, although it must be noted that both I27+Q_35_ (used for our CD experiments) and I27+Q_62_ showed equivalent fibrillogenic behavior when followed by TEM (see below). (C) Aggregation of polyQ samples over the incubation period at 37°C monitored by turbidimetry at 405 nm. Only the I27+Q_35_ and I27+Q_62_ samples aggregated. QBP1 inhibits the aggregation of I27+Q_62,_ as described previously for the Q_62_ tract fused to thioredoxin [5]. (D) TEM images of I27+Q_19_ (1), I27+Q_35_ (2), I27+Q_62_ (3), and I27+Q_62_+QBP1 (4), all after a 32-d incubation at 37°C. The protein concentration was 12 µM while that of QBP1 was 20 µM. No amyloid fibers were observed in I27+Q_19_ and I27+Q_62_+QBP1 samples. The images were acquired at 30,000× (panels 1, 4) and 60,000× (panels 2, 3). Scale bars correspond to 0.6 (panels 1, 4) and 0.3 µm (panels 2, 3).
(TIF)

Figure S3 **Controls for VAMP2 structure and aggregation in the carrier protein.** (A) Far-UV CD spectra (left), and estimated increase in β-structure content over the incubation time at 37°C (right). Considering that the experimental uncertainty is about 1% Δβ, no significant increase in β-structure over time was observed for any of these proteins. (B) ^1^H NMR. The VAMP2 spectrum (middle, blue) shows resonances whose chemical shift values and line widths, and lack of signals (right), are hallmarks of a RC. In the spectrum of the carrier-guest (bottom, red), signal broadening and retention of native-like I27 signals were observed, indicating that I27 remains folded while VAMP2 is still disordered. An additional 2D ^1^H NOESY spectrum (not shown) allowed us to assign unambiguously backbone amide, aliphatic, aromatic, and hydroxyl proton resonances arising from the tertiary structure of I27 and to definitively corroborate that I27 can adopt its native folded structure when hosted VAMP2. In this spectrum, no NOEs between I27 and VAMP2 could be unambiguously identified (data not shown), suggesting the lack of interaction between both proteins in the construct. (C) As expected, given the non-amyloidogenic nature of this IDP, VAMP2 did not aggregate under any of the conditions studied [35]. (D) TEM images of the I27-MCS (1), ubi-MCS (2), VAMP2 (3), and I27+VAMP2 (4) samples, used as negative controls for fibrillogenesis over 30-d incubation at 37°C. The protein concentration was 40 µM. None of these samples formed amyloid fibers and, instead, the images revealed clumps of amorphous aggregates. Images were acquired at 30,000× and the scale bars correspond to 0.6 µm.
(TIF)

Figure S4 **Controls for Aβ42 structure and aggregation in the carrier protein.** (A) Far-UV CD spectra (left), and estimated increase in β-structure content during the incubation time at 37°C (right). Moreover, F19S/L34P Aβ42 does not undergo structural transitions, upon incubation at 37°C, and the presence of the QBP1 and SV111 peptides do not appear to affect this process (right, in close agreement with our SMFS results, Figure 3). (B) ^1^H NMR spectroscopy. Signals of the native I27 carrier (top, black) are retained with only slight shifts in the variants bearing the Aβ42 (red), Arc Aβ42 (green), and F19S/L34P Aβ42 (bottom, dark red) grafts indicating that the I27 carrier retains its native, folded structure. Signals around 8.5–8.0, 7.3–7.0, and 0.9 ppm increase in size in the presence of the guests, in line with previous observations that are consistent with hydrogens of amide, aromatic and methyl groups of Aβ42, respectively, in a predominantly RC conformation [61],[62]. (C) Aggregation of I27+Aβ42 samples over the incubation period at 37°C. The presence of QBP1 does not appear to affect the aggregation of Aβ42. By contrast, the SV111 peptide does inhibit the aggregation of I27+Aβ42, as previously reported for isolated Aβ42 [38]. F19S/L34P Aβ42 contains two point mutations reported to impede Aβ42 aggregation [39] and, accordingly, it does not aggregate in our experimental conditions. (D) TEM images of the I27+Aβ42 (1), I27+Arc Aβ42 (2), I27+Arc Aβ42+QBP1 (3), I27+Aβ42+SV111 (4), I27+F19S/L34P Aβ42 (5), Aβ42 (6), and Aβ42+QBP1 (7) samples after 32 d at 37°C, except for 6 and 7 whose images were taken after 13-d incubation. The protein, QBP1 and SV111 concentrations were 10, 20, and 100 µM, respectively, except for images 6 and 7, where the concentration of the Aβ42 protein alone corresponds to 50 µM while the concentration of QBP1 (7) was 500 µM. These observations closely reflect the expected results. No amyloid fibers were observed in the presence of SV111 or for F19S/L34P Aβ42, while QBP1 appeared to have no effect on the amyloidogenic capability of Aβ42 (even at high concentrations). Images were acquired at 30,000× (1, 4), 50,000× (2), 40,000× (3), 20,000× (5–7). Scale bars correspond to 0.6 (1, 4), 0.35 (2), 0.45 (3), and 0.9 µm (5–7).
(TIF)

Figure S5 **Controls for α-synuclein structure and aggregation in the carrier protein.** (A) Far-UV CD spectra (left), and estimated increase in β-structure content during the incubation at 37°C (right). The ubiquitin module shows a typical α/β spectrum [63], α-synuclein proteins present characteristic RC spectra [64]. The carrier-guest protein spectra fairly agree with the addition of carrier and guest spectra weighted by the relative contribution of respective sequences to the fusion protein (not shown). This indicates that α-synuclein inserted into ubiquitin remains essentially disordered. In the presence of a membrane mimetic, SDS, α-synuclein as a guest within the ubiquitin carrier also appears to adopt a highly α-helical conformation as previously detected by CD and NMR [9]. Accordingly, we found that α-synuclein and ubi+α-synuclein showed α-helical structure in the presence of 1 mM SDS. When isolated or as a guest with ubiquitin, α-synucleins acquire β-structure, during incubation at 37°C, an effect that is suppressed or slowed down by the presence of QBP1 (right). (B) The 1H NMR spectra of A53T α-synuclein (blue) and ubi+A53T α-synuclein (red) are largely comparable, except for very slight changes (mainly at <1 ppm and ∼8 ppm). This indicates that the behavior of A53T α-synuclein is similar when isolated or inserted into ubiquitin and no strong interactions occur between the two proteins in the carrier-guest protein. Furthermore, the chemical shift values of the resonances in the upfield region in the ^1^H NMR spectra for ubi+MCS (black) and ubi+A53T α-synuclein (red) are very similar. Since these values are exquisitely sensitive to local environment, this similarity is conclusive evidence that the hydrophobic core packing in ubiquitin is unaffected by the presence of the A53T α-synuclein guest. In the spectrum, the broader lines of ubiquitin carrying the A53T α-synuclein guest relative to ubi+MCS are consistent with the larger size and slower tumbling of the former (right). A53T α-synuclein lacks the upfield signals and its complete ^1^H spectrum is typical of a RC. The residues assigned correspond to the folded ubiquitin structure [65]. Furthermore, crosspeaks between aliphatic and aromatic protons, such as Phe45 and Ile61 or Tyr59 and Leu50, are observed in the 2D ^1^H NOESY spectrum (data not shown), indicating that the fold of ubiquitin is maintained when it hosts α-synuclein. Thus, the spectra indicate that the two fused proteins maintain a high degree of structural integrity. (C) Aggregation of α-synuclein samples monitored by turbidimetry at 405 nm. The α-synuclein–containing proteins (isolated or fused to the ubiquitin carrier module) aggregated while the isolated carrier module did not. Addition of QBP1 appears to reduce the aggregation of ubi+A53T α-synuclein, although to a lesser extent than that of Q_62_ (Figure S2C). (D) 1–4: TEM images of A53T α-synuclein (1), ubi+A53T α-synuclein (2, 4), and ubi+A53T α-synuclein+QBP1 (3) after 22 d at 37°C. The amyloid fibers observed for A53T α-synuclein and ubi+A53T α-synuclein were closely comparable. Protein concentration was 10 µM and QBP1 concentration was 20 µM. No fibers were observed for ubi+A53T α-synuclein+QBP1. Images were acquired at 60,000× (1), 40,000× (2), 30,000× (3), and 120,000× (4). Scale bars correspond to 0.3, 0.45, 0.6, and 0.15 µm, respectively. 5: AFM image in liquid of ubi+A53T α-synuclein after 52 d at 37°C, acquired using the dynamic mode as described previously [66]. Scale bar corresponds to 50 nm.
(TIF)

Figure S6 **Controls for Sup35NM structure and aggregation in the carrier protein.** (A) Far-UV CD spectra (left), and estimated increase in β-structure content during the incubation at 37°C (right). The CD spectra indicate that the Sup35NM prion remains disordered when fused to the I27 carrier module (the CD spectrum of I27+Sup35NM closely resembles the sum of I27 and Sup35NM spectra weighted by the relative contribution of their respective sequences to the fusion protein). Whereas the spectrum at 5 d (inset) shows features indicating that RC conformations are still predominant, spectral analysis reveals a significant increase in β-structure (right panel). Remarkably, the CD spectrum at 5 d has lost the majority of its intensity, possibly because of oligomerization and the formation of insoluble aggregates (see [C]). QBP1 (20 µM) appears to suppress these oligomerization processes and to reduce the formation of β-structure. Apparently, I27+Sup35NM acquires more β-structure than the isolated Sup35NM. This could be attributed to the positioning of the NP termini in close proximity when it is nested within I27, which might encourage the formation of contacts. (B) The ^1^H NMR spectra of I27-MCS (black), Sup35NM (blue), and I27+Sup35NM (red) are shown on the left with an expanded view of the HN resonances on the right. The line broadening of the I27+Sup35NM spectrum is more evident than that observed for the other carrier-guest proteins examined (Figures S2B, S3B, S4B, S5B), probably due to this being the largest guest studied (which is in a RC conformation) and/or the formation of the oligomers and aggregates inferred by CD (A). Therefore, to confirm that the native structure of the I27 carrier is maintained, a 2D ^1^H NOESY spectrum was recorded. In this spectrum, signals arising from the packing of aliphatic and aromatic groups in the native hydrophobic core of I27 were observed and rigorously assigned, thus allowing us to conclude that the native conformation of I27 is still compatible with the Sup35NM graft (data not shown). (C) Aggregation of Sup35NM samples. Sup35NM, either isolated or fused to the I27 carrier, aggregated over the incubation period at 37°C. The presence of QBP1 impedes (or at least reduces) aggregation of Sup35NM. (D) TEM images of Sup35NM (1), Sup35NM+QBP1 (2), I27+Sup35NM (3), I27+Sup35NM+QBP1 (4), Sup35NM (6), I27+Sup35NM (7), and pFS-2+Sup35NM (8). Samples for panels 1–4 were observed after a 32-d incubation at 37°C, while samples for panels 6–8 were incubated for 29 d at 4°C. The concentrations of pFS-2 polyprotein, carrier-guest protein, guest protein, and QBP1 were 20, 10, 10, and 20 µM, respectively. The presence of amyloid fibers was reduced by QBP1 peptide, both in Sup35NM (2) and I27+Sup35NM (4) samples, although fibers remained visible. Amyloid formation was reduced at 4°C (the temperature used for protein storage before SMFS experiments). Although still visible, the number of fibers formed by pFS-2+Sup35NM (8) were considerably less than Sup35NM (6) or I27+Sup35NM (7) at the same conditions. The TEM images were acquired at 60,000× (1, 2), 40,000× (3), 30,000× (4), 100,000× (6), 120,000× (7), and 80,000× (8). The AFM image (5) was acquired using the dynamic mode in liquid as described previously [66]. Scale bars correspond to 0.3 (1, 2), 0.45 (3), 0.6 (4), 0.5 (5), 0.18 (6), 0.15 (7), and 0.22 (8) µm.
(TIF)

Figure S7 **Congo Red binding assay.** Concentration (µM) of Congo Red dye bound to the amyloid aggregates formed by representative NPs (upper panel), as calculated through the bathochromic and hyperchromic shifts undergone by the dye upon specific binding to amyloid aggregates [39]. Isolated Sup35NM was used as a positive control for these measurements. The bottom panel shows an optical fluorescence micrograph taken with a 63× objective showing amyloid aggregates formed by I27+Sup35NM bound to Congo Red.
(TIF)

Figure S8 **Standard heteropolyprotein approach (in series) for the nanomechanical analysis of polyQ tracts.** (A) Cartoon representing the heteropolyprotein strategy used in this analysis: (I27)_4_-Q_n_-(I27)_3_. This strategy is conceptually similar to that recently used for the nanomechanical analysis of α-synuclein [26]. Two lysine residues were placed flanking the polyQ tracts to enhance the solubility of the heteropolyproteins [67]. (B) SMFS spectra of the indicated polyproteins. The left traces show typical recordings of a NM conformation for the polyQ tracts (in orange) while several possible M conformers of the protein (I27)_4_-Q_51_-(I27)_3_ are shown on the right (in red). However, as these force peaks lie in the frequently contaminated proximal region of the recordings, one cannot unequivocally demonstrate that they originate from true M conformers of the NP monomer [45]. (C) Turbidimetry of (I27)_4_-Q_n_-(I27)_3_ samples, monitored by absorbance at 405 nm over the incubation period at 37°C. The lack of turbidity suggests an inability to aggregate [5]. (D) TEM image of (I27)_4_-Q_51_-(I27)_3_ after 28 d at 37°C (left). Proteins were used at a concentration of 10 µM and no fibers were observed. The image was acquired at 30,000× and the scale bar corresponds to 1 µm. AFM image of (I27)_4_-Q_51_-(I27)_3_ incubated for 30 d at 37°C (right) in which no amyloid fibers were observed. The image was acquired using the dynamic mode in liquid conditions as described previously [66]. Scale bar corresponds to 0.5 µm.
(TIF)

Figure S9
**Thermodynamic stability of the carrier protein upon insertion of the guest.** (A) Thermal denaturation parameters derived by CD spectroscopy assuming a two-state (N↔D) equilibrium model with pre- and post-transition baselines linearly depending upon temperature [68]. The half transition temperature (*T*_m_) and the van't Hoff enthalpy change (Δ*H*_vH_) were directly derived from fitting while the entropy change of unfolding (Δ*S*) was calculated using the Gibbs equation [69]. The change in the conformational stability relative to the I27-MCS variant (ΔΔ*G*) was estimated using the following equation:
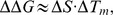
where Δ*T*_m_ is the difference in the *T*_m_ value for a variant relative to I27-MCS [70]. The Δ*S* value used was 193 cal·mol^−1^·K^−1^, which is the mean value for all the variants. (B) Differential scanning calorimetry (DSC) curves of indicated proteins and summary of the denaturation parameters (*T*_mexp_ is the *T*-value at *Cp*_max_; Δ*H*_exp_ the enthalpy change estimated by integration of the DSC curve; *T*_m_, Δ*H*_cal_, and Δ*H*_vH_ the half transition temperature, and the calorimetric and the van't Hoff enthalpy changes estimated from the endotherm deconvolution; and Δ*T*_h–h_ the curve width at half-height). Insertion of A30P or A53T α-synuclein into the ubiquitin carrier significantly increases the denaturation enthalpy when compared to insertion of the wt α-synuclein. Moreover, the ratio between Δ*H*_cal_ and Δ*H*_vH_ is above unity, suggesting a tendency towards intermediate formation during unfolding. This effect disappears upon the addition of an excess (50 µM) of QBP1 to ubi+A53T α-synuclein samples, which then unfold following the two-state denaturation model.(TIF)

Figure S10 **Lack of correlation between** ***F*** **and Δ** ***L*** **_c_.** Scatter plots show that there is no correlation between *F* and Δ*L*_c_ values for: (A) polyQ tracts, (B) Aβ42, (C) α-synuclein, and (D) Sup35NM proteins. No clustering in these plots was observed for any of the NPs studied, strongly suggesting that no specific regions in the NPs mediate their mechanical stability. Furthermore, treatment of samples with QBP1/SV111 peptides did not result in the loss of any specific population of Δ*L*_c_ data points. In principle, this observation prevents us from extracting any structural information regarding the mechanism of QBP1 and SV111 binding to NPs.
(TIF)

Figure S11 **Effect of familial-disease mutations and QBP1 on the number of structured regions per molecule.** The average number of force peaks per molecule is shown for: (A) polyQ tracts; (B) Aβ42; (C) α-synuclein; and (D) Sup35NM proteins. NM conformations correspond to 0 force peaks. These events are excluded from the average calculations shown in the figure. The familial-disease mutations increase the number of force peaks per molecule, suggesting that they increase the propensity to acquire M structures. By contrast, QBP1 reduces this number for Q_62_ and Arc Aβ42 but not for A53T α-synuclein or Sup35NM. Additional controls are required to test if these average differences are significant. SV111 does not reduce this number in Aβ42.
(TIF)

Figure S12 **Putative additional hM events.** (A) Example of an event considered as a putative hM. In gray we show the force peak due to the unfolding of the carrier module (C) and a region of the NP that is shorter than its full length. The Δ*L*_c_ value associated with this force peak is larger than that of a normal ubiquitin repeat but not as large as that expected if the entire NP was stretched. To explain these frequent events, we assume that the polyprotein attachments (either from the tip or the substrate) were not maintained until the end of the experiment (i.e., the polyprotein molecule may have detached before reaching the forces required to fully unfold the region trapped [28]). Therefore, it is reasonable to assume that its mechanical stability would be higher than the force of detachment (last peak in the spectrum, indicated by the arrow). (B–E) Force of detachment values for the recordings considered to contain putative additional events for polyQ tracts, Aβ42, α-synuclein, and Sup35NM, respectively. Putative hM events (with detachment forces ≥400 pN) are shown in black. This analysis suggests that the population of hM conformers observed directly in our study represents an under-estimate of the actual propensity of the NPs to form these conformers. Thus, it appears that SMFS experiments miss a considerable fraction of hM conformers owing to the experimental approach, which seems to filter out several hM events. These data were not included in our sample size (n). In (B–E) each vertical bar represents a single event (one molecule). Note that Q_62+_QBP1 and Sup35NM+QBP1 yielded no putative hM events and therefore are not represented here.
(TIF)

Figure S13 **Intrinsic disorder predictions for the fusion polyproteins/proteins used in the study.** PONDR values below 0.5 indicate a tendency to form ordered structures, while those over 0.5 indicate a tendency for disorder. PONDR VLXT and PONDR VL3 data are shown as red and blue lines, respectively. (A) pFS-2 (left) and pFS-2/I27 (I27 as the carrier protein, right): these predictions show a reasonable correlation with the proteins contained in the pFS polyproteins, where the disordered region corresponds to the N2B stretch while the ordered regions correspond to the I27/ubi repeats (Figure 1A, [30]). (B) I27-MCS (left) and ubi-MCS (right). (C–G) NPs/VAMP2 alone (top, guest) and carrier-guest proteins (bottom), as indicated in the figure. The corresponding guest sequences are shaded yellow in all plots. Scales for guest and carrier/guest predictions were normalized such that the region of interest is same length in both plots. The ends of the regions sharing common sequences were located in the same *x* position to facilitate data comparison of guest and carrier/guest proteins. As Q_35_ and Q_62_ start at the same position in both constructs ([C] top and bottom) but are of different lengths, the corresponding NP shading starts at the same position but extends differently. The first yellow shaded area corresponds to Q_19_, while Q_35_ extends to the end of the grey shaded region and Q_62_ to the end of the second yellow shaded zone. The non-shaded region in the guest plots corresponds to different residues in the protein used, which do not belong to the guest sequence (i.e., purification tags and residues added in polyQ expansions [71]). PONDR VLXT and VL3 use different parameters and window sizes, and were trained on very different datasets; as a result, their predictions differ considerably, especially for longer NPs ([F] α-synuclein and [G] Sup35NM). Nonetheless, all the NPs (except polyQ tracts, as discussed in Text S1) contain segments that are predicted to be ordered, in agreement with our SMFS data. Our results suggest that the structured regions predicted here must be small and fluctuating to be compatible with the overall RC behavior observed in CD/NMR (Figures S2, S3, S4, S5, S6).
(TIF)

Table S1 **Summary of controls for structure, aggregation, and fibrillogenesis of the NP.** Experiments were carried out with the carrier-guest proteins alone (unless otherwise stated). ND, not determined; +, positive result; −, negative result. Abbreviations for the techniques are provided in the text. iAFM, imaging AFM. QBP1 and SV111 are inhibitors referred to in the main text and described in the Text S1 supplementary methods section.
(DOC)

Table S2 **Mechanical stability (** ***F*** **) of the carrier proteins upon insertion of guest proteins.** The *F*-values of the carriers reveal a significant decrease in their mechanical stability upon insertion of the guest proteins (both in the NM or M conformations) when compared to the carriers alone (204±26 pN for I27 [72] and 203±35 for Ubi [73], at comparable pulling speeds). This appears to indicate that the guest protein affects the mechanical stability of the carriers. Considering that our criteria for selection of SMFS recordings imply the observation of the force peak for the carrier unfolding, our SMFS data may be an underestimation (Table 1). SD, standard deviation.
(DOC)

Table S3 **Putative M events and number of events per molecule.** # peaks/molecule, average number of M regions (force peaks) per molecule. The values reported are calculated considering only M conformers, excluding those monomers exhibiting a NM conformation (which result in 0 force peaks; see Figure S11). Error calculations are explained in the Text S1 supplementary methods.
(DOC)

Table S4 **Summary of the oligonucleotides used to design the I27 carrier module and for the cloning of NPs.** The restriction sites introduced by PCR into the amplified sequences are highlighted in italics. Mutagenic oligonucleotides are indicated by (Mut) and the mutations introduced are highlighted in italics and bold. The extra sequences added to the terminus of each restriction site were chosen on the basis of recommendations from New England Biolabs, which enhance the digestion efficiency of linear DNA sequences. All oligonucleotides used were purchased from Sigma-Aldrich.
(DOC)

Text S1 **Supplementary results, discussion, methods, and references.**
(DOC)

## Abbreviations

Δ*L*_c_: contour length increase
AFM: atomic force microscopy
Arc: arctic
CD: circular dichroism
DSC: diferential scanning calorimetry
*F*: unfolding force
hM: hyper-mechanostable
IDP: intrinsically disordered protein
M: mechanostable
MCS: multicloning site
NM: non-mechanostable
NMR: nuclear magnetic resonance
NP: neurotoxic protein
pFS: plasmid for force spectroscopy
QBP1: polyQ-binding peptide 1
RC: random coil
SCA: spinocerebellar ataxia
SDS: sodium dodecyl sulfate
SMFS: single-molecule force spectroscopy
TEM: transmission electron microscopy
WLC: worm-like chain
wt: wild-type
