# Supplementary figures and images for: Common Features at the Start of the Neurodegeneration Cascade
Source: PLoS Biol. 2012 May 29;10(5):e1001335. doi: 10.1371/journal.pbio.1001335 (PMC3362641; doi:10.1371/journal.pbio.1001335)

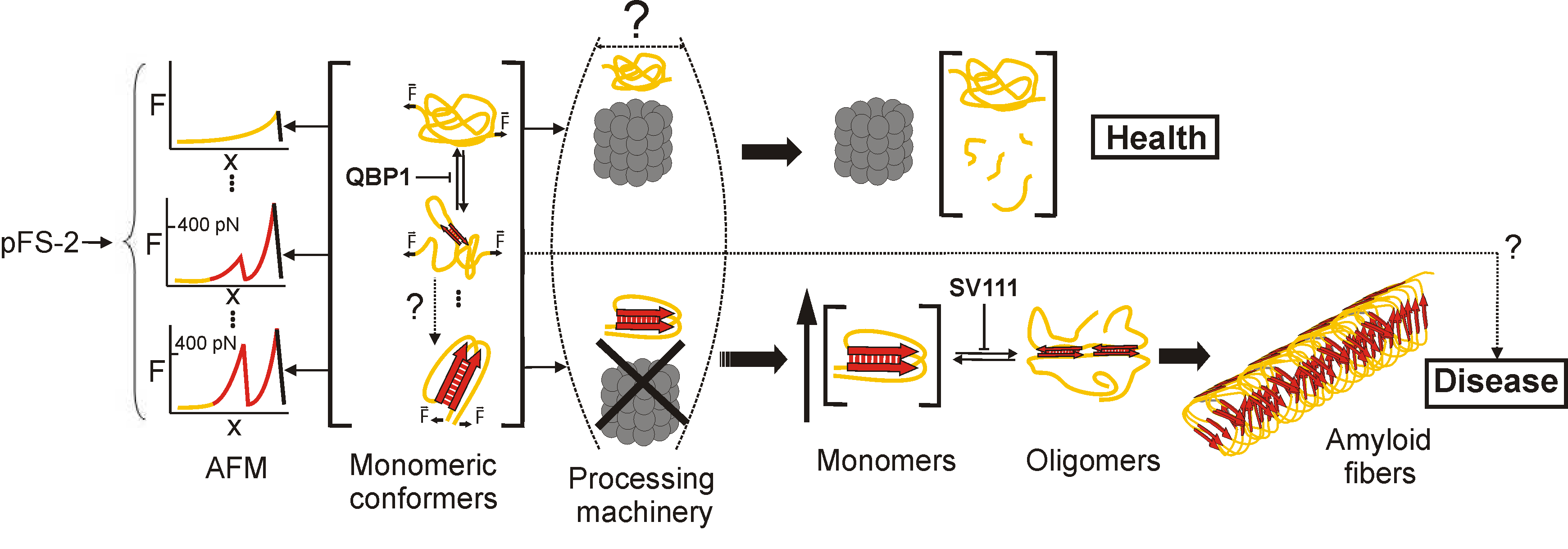

Supplement: Figure S1 — Mechanical hypothesis of the primary cause for amyloid-related neurodegenerative diseases. The use of the newly developed pFS-2 vector [30] has allowed us to observe a high degree of conformational polymorphism in different NPs, comprising conformations with no detectable mechanical stability (NM conformers, that exhibit no force peaks in SMFS) and M conformations (with at least one force peak), the latter including the subset of hM conformers (with at least one force peak with F≥400 pN). As discussed in the main text, it is reasonable to assume that both M and hM conformers are β-structured. These different conformers are thought to be in a highly dynamic equilibrium [21]; however, once formed the hM conformers may be kinetically trapped by a high unfolding barrier. We tentatively propose a plausible mechanism of cytotoxicity in which hM conformers mechanically block the cell's protein-processing machinery leading to an increase in their cytosolic concentration and affecting multiple cellular processes, which ultimately results in the formation of oligomers and amyloid fibers. These hM conformers may originate sequentially from less toxic intermediate β-structured conformations (on-pathway) or they may trigger the disease through a pathway other than that proposed here. Alternatively, hM conformers may represent kinetically trapped by-products of the amyloidogenic/cytotoxic pathway. The steps blocked by the two inhibitor peptides used in this study are indicated: QBP1 blocks the conformational change and displaces the putative RC/β-structure equilibrium towards the RC conformer (thereby interrupting the pathogenic cascade in its earliest stages), while SV111 inhibits the oligomerization of the β-hairpin monomers of Aβ42 [38]. (TIF) [file pbio.1001335.s001.tif]

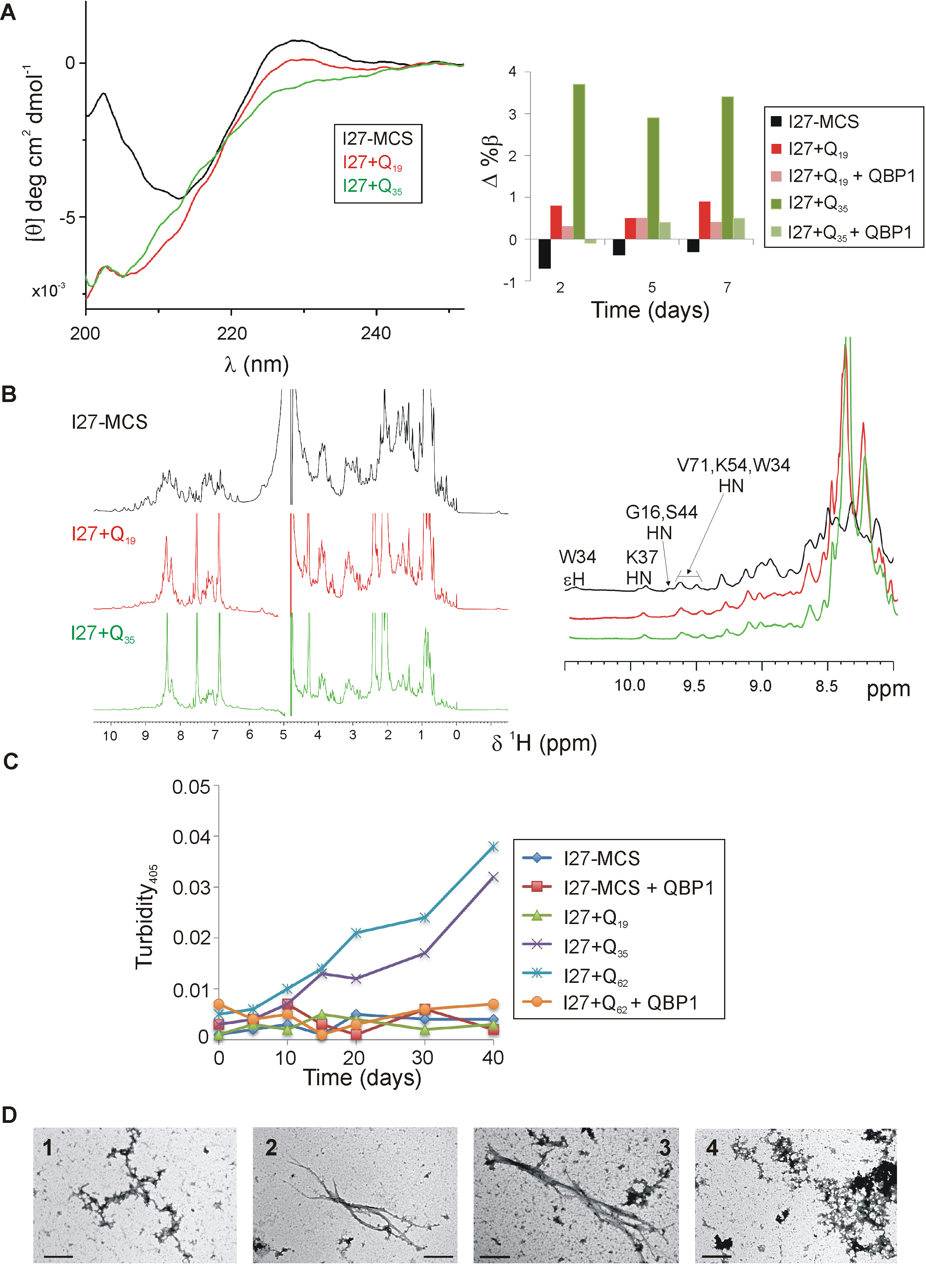

Supplement: Figure S2 — Controls for polyQ tract structure and aggregation in the carrier protein. (A) Far-UV CD spectra (left) and estimated increase in β-structure content (Δ%β, relative to day zero) over the incubation period at 37°C (right). The features of the CD spectra indicate that the Q19 and Q35 tracts fused to the I27 carrier remain disordered ([θ] = molar ellipticity per residue). In addition, QBP1 appears to reduce the increase in the content of β-structure in I27+Q35. (B) 1H NMR. Left, complete 1D spectra; right, zoom of HN region where representative resonances of the I27 moiety are labeled [59]. The spectrum of I27-multicloning site (MCS, top) is very similar to that of the fully folded wt protein [59], and it exhibits the extensive chemical shift dispersion typical of structured proteins. In the constructs containing Q19 (middle, red) or Q35 (bottom, green) the I27-MCS spectrum is retained. Since the 1H chemical shift is exquisitely sensitive to the local environment, these data are unequivocal evidence that the native backbone structure is maintained when the carrier hosts the MCS, Q19, or Q35. Rather sharp peaks appear in the variants containing Q19 or Q35. These signals can be assigned to glutamine residue resonances: backbone HN, 8.4 ppm; sidechain H2N, 7.50 and 6.84; Hα, 4.3; Hγ, 2.3; Hβ, 2.1; and Hβ′, 2.0 ppm. The sharpness of these signals and the similarity of the chemical shift values to the reference values for glutamine in an RC conformation [60] indicate that these polyQ segments are disordered when grafted in I27. CD and NMR experiments were not carried out for I27+Q62 because of the difficulty in producing the protein in the required concentration, although it must be noted that both I27+Q35 (used for our CD experiments) and I27+Q62 showed equivalent fibrillogenic behavior when followed by TEM (see below). (C) Aggregation of polyQ samples over the incubation period at 37°C monitored by turbidimetry at 405 nm. Only the I27+Q35 and I27+Q62 samples aggregated. Q [file pbio.1001335.s002.tif]

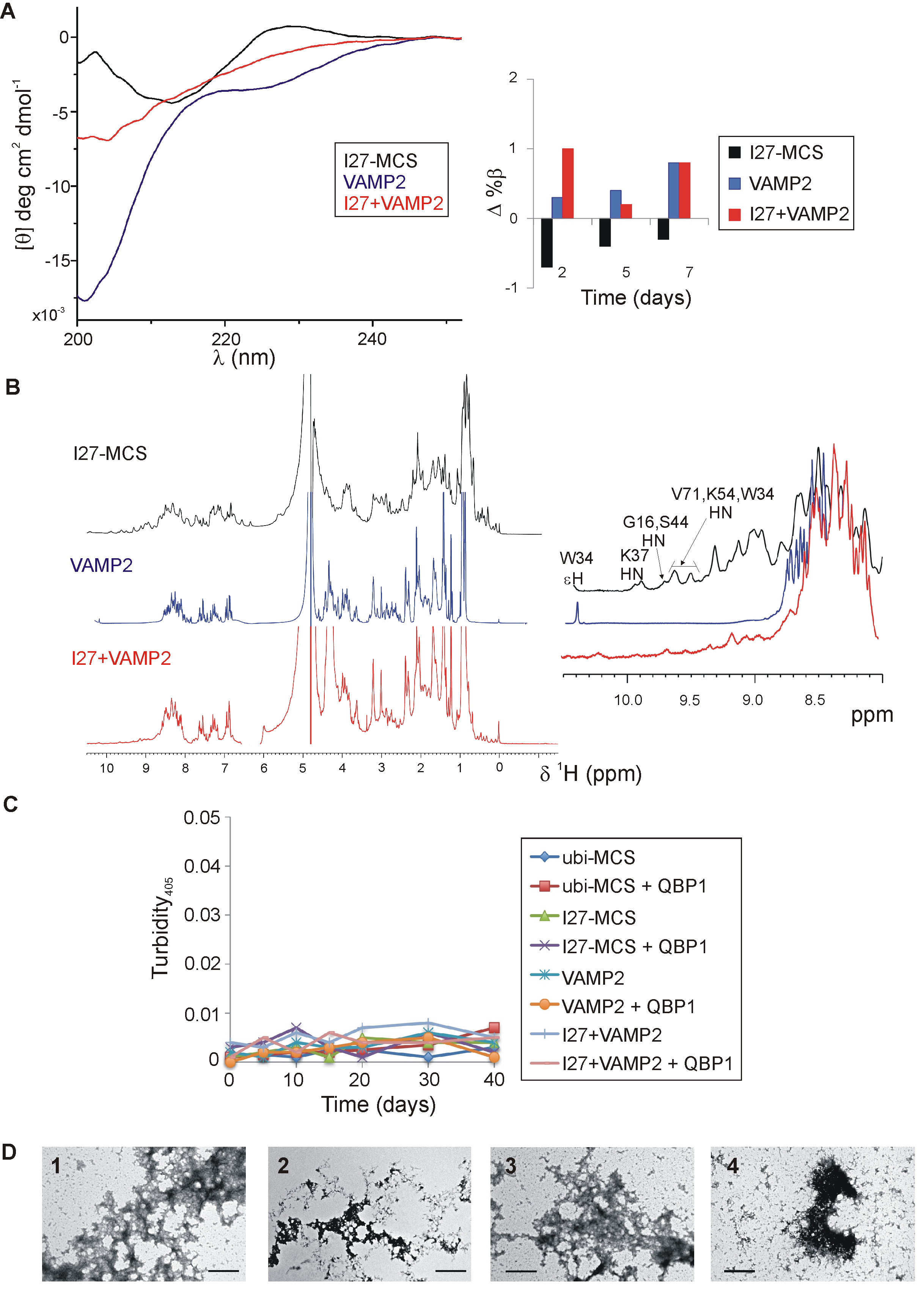

Supplement: Figure S3 — Controls for VAMP2 structure and aggregation in the carrier protein. (A) Far-UV CD spectra (left), and estimated increase in β-structure content over the incubation time at 37°C (right). Considering that the experimental uncertainty is about 1% Δβ, no significant increase in β-structure over time was observed for any of these proteins. (B) 1H NMR. The VAMP2 spectrum (middle, blue) shows resonances whose chemical shift values and line widths, and lack of signals (right), are hallmarks of a RC. In the spectrum of the carrier-guest (bottom, red), signal broadening and retention of native-like I27 signals were observed, indicating that I27 remains folded while VAMP2 is still disordered. An additional 2D 1H NOESY spectrum (not shown) allowed us to assign unambiguously backbone amide, aliphatic, aromatic, and hydroxyl proton resonances arising from the tertiary structure of I27 and to definitively corroborate that I27 can adopt its native folded structure when hosted VAMP2. In this spectrum, no NOEs between I27 and VAMP2 could be unambiguously identified (data not shown), suggesting the lack of interaction between both proteins in the construct. (C) As expected, given the non-amyloidogenic nature of this IDP, VAMP2 did not aggregate under any of the conditions studied [35]. (D) TEM images of the I27-MCS (1), ubi-MCS (2), VAMP2 (3), and I27+VAMP2 (4) samples, used as negative controls for fibrillogenesis over 30-d incubation at 37°C. The protein concentration was 40 µM. None of these samples formed amyloid fibers and, instead, the images revealed clumps of amorphous aggregates. Images were acquired at 30,000× and the scale bars correspond to 0.6 µm. (TIF) [file pbio.1001335.s003.tif]

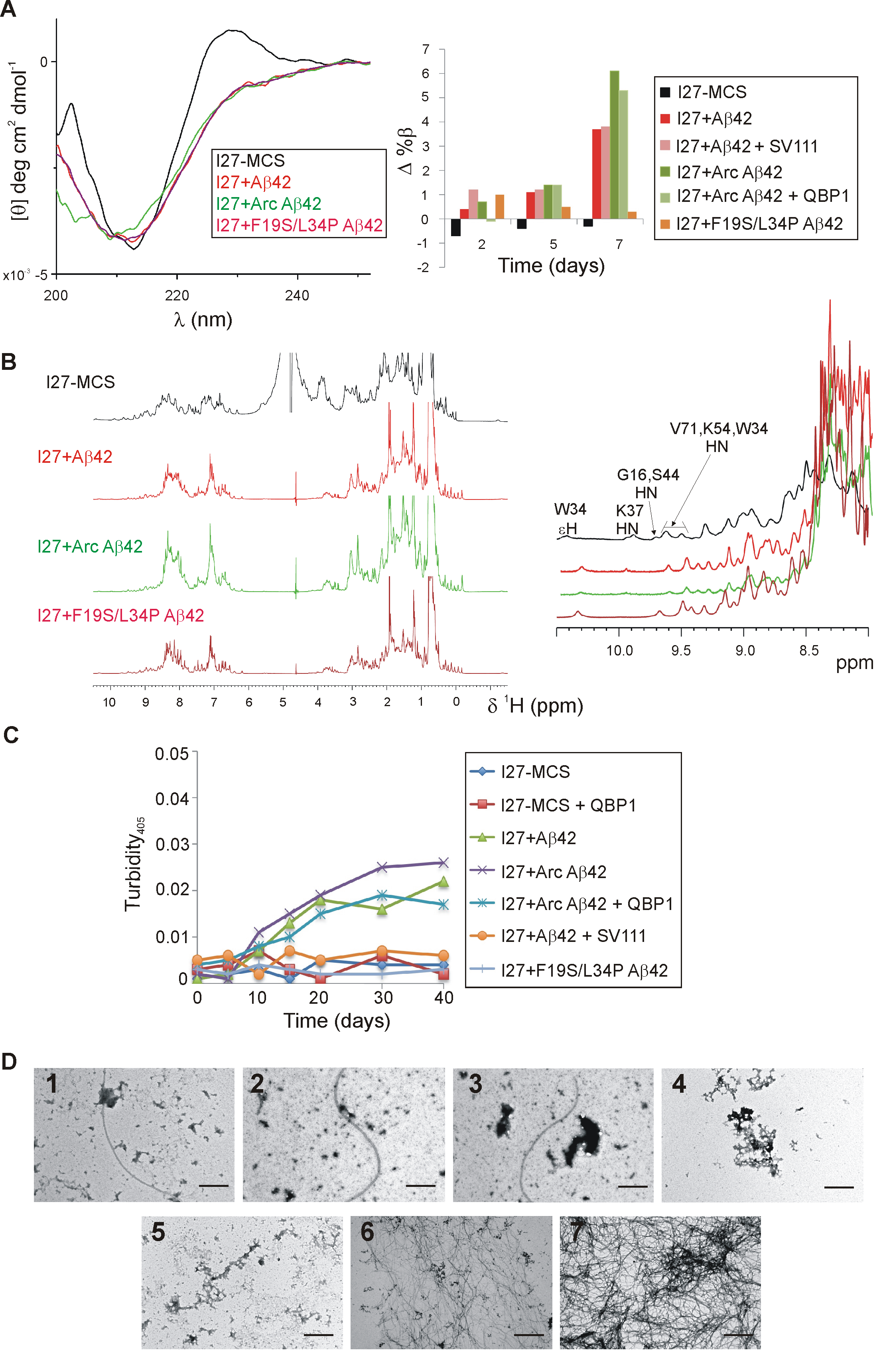

Supplement: Figure S4 — Controls for Aβ42 structure and aggregation in the carrier protein. (A) Far-UV CD spectra (left), and estimated increase in β-structure content during the incubation time at 37°C (right). Moreover, F19S/L34P Aβ42 does not undergo structural transitions, upon incubation at 37°C, and the presence of the QBP1 and SV111 peptides do not appear to affect this process (right, in close agreement with our SMFS results, Figure 3). (B) 1H NMR spectroscopy. Signals of the native I27 carrier (top, black) are retained with only slight shifts in the variants bearing the Aβ42 (red), Arc Aβ42 (green), and F19S/L34P Aβ42 (bottom, dark red) grafts indicating that the I27 carrier retains its native, folded structure. Signals around 8.5–8.0, 7.3–7.0, and 0.9 ppm increase in size in the presence of the guests, in line with previous observations that are consistent with hydrogens of amide, aromatic and methyl groups of Aβ42, respectively, in a predominantly RC conformation [61],[62]. (C) Aggregation of I27+Aβ42 samples over the incubation period at 37°C. The presence of QBP1 does not appear to affect the aggregation of Aβ42. By contrast, the SV111 peptide does inhibit the aggregation of I27+Aβ42, as previously reported for isolated Aβ42 [38]. F19S/L34P Aβ42 contains two point mutations reported to impede Aβ42 aggregation [39] and, accordingly, it does not aggregate in our experimental conditions. (D) TEM images of the I27+Aβ42 (1), I27+Arc Aβ42 (2), I27+Arc Aβ42+QBP1 (3), I27+Aβ42+SV111 (4), I27+F19S/L34P Aβ42 (5), Aβ42 (6), and Aβ42+QBP1 (7) samples after 32 d at 37°C, except for 6 and 7 whose images were taken after 13-d incubation. The protein, QBP1 and SV111 concentrations were 10, 20, and 100 µM, respectively, except for images 6 and 7, where the concentration of the Aβ42 protein alone corresponds to 50 µM while the concentration of QBP1 (7) was 500 µM. These observations closely reflect the expected results. No amyloid fibers were observed in the presence of SV111 or for F19S/L34P A [file pbio.1001335.s004.tif]

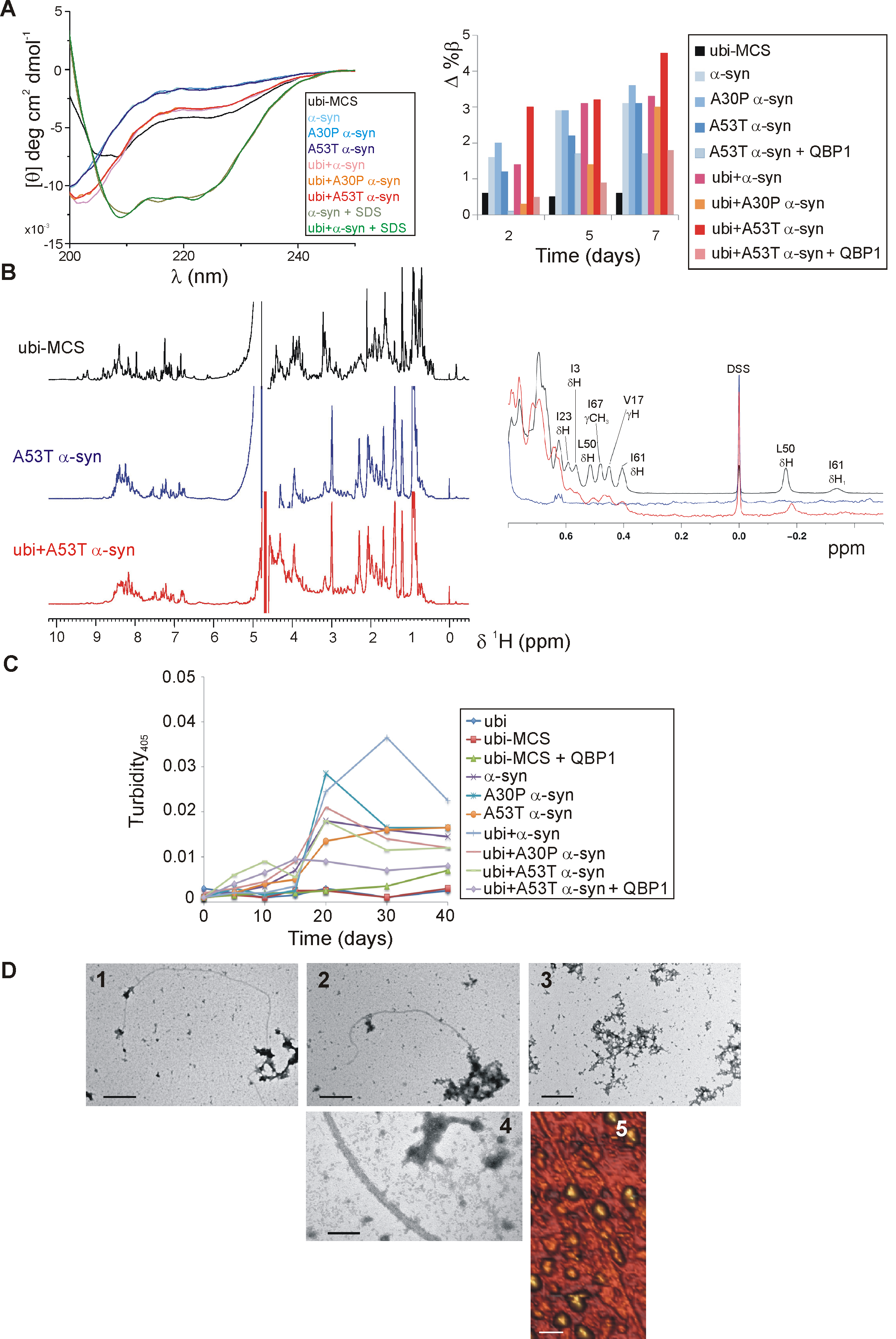

Supplement: Figure S5 — Controls for α-synuclein structure and aggregation in the carrier protein. (A) Far-UV CD spectra (left), and estimated increase in β-structure content during the incubation at 37°C (right). The ubiquitin module shows a typical α/β spectrum [63], α-synuclein proteins present characteristic RC spectra [64]. The carrier-guest protein spectra fairly agree with the addition of carrier and guest spectra weighted by the relative contribution of respective sequences to the fusion protein (not shown). This indicates that α-synuclein inserted into ubiquitin remains essentially disordered. In the presence of a membrane mimetic, SDS, α-synuclein as a guest within the ubiquitin carrier also appears to adopt a highly α-helical conformation as previously detected by CD and NMR [9]. Accordingly, we found that α-synuclein and ubi+α-synuclein showed α-helical structure in the presence of 1 mM SDS. When isolated or as a guest with ubiquitin, α-synucleins acquire β-structure, during incubation at 37°C, an effect that is suppressed or slowed down by the presence of QBP1 (right). (B) The 1H NMR spectra of A53T α-synuclein (blue) and ubi+A53T α-synuclein (red) are largely comparable, except for very slight changes (mainly at <1 ppm and ∼8 ppm). This indicates that the behavior of A53T α-synuclein is similar when isolated or inserted into ubiquitin and no strong interactions occur between the two proteins in the carrier-guest protein. Furthermore, the chemical shift values of the resonances in the upfield region in the 1H NMR spectra for ubi+MCS (black) and ubi+A53T α-synuclein (red) are very similar. Since these values are exquisitely sensitive to local environment, this similarity is conclusive evidence that the hydrophobic core packing in ubiquitin is unaffected by the presence of the A53T α-synuclein guest. In the spectrum, the broader lines of ubiquitin carrying the A53T α-synuclein guest relative to ubi+MCS are consistent with the larger size and slower tumbling of the former (right) [file pbio.1001335.s005.tif]

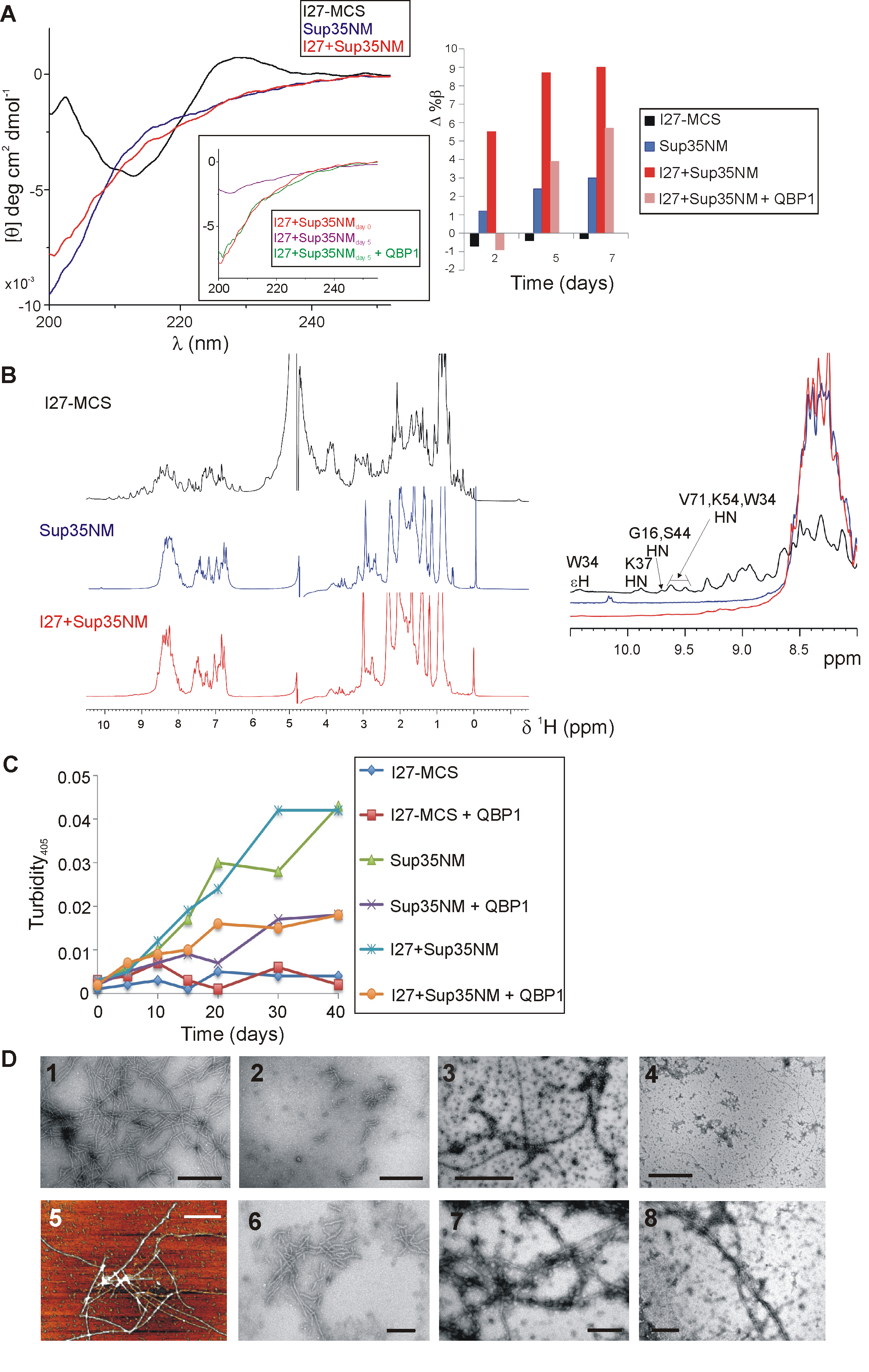

Supplement: Figure S6 — Controls for Sup35NM structure and aggregation in the carrier protein. (A) Far-UV CD spectra (left), and estimated increase in β-structure content during the incubation at 37°C (right). The CD spectra indicate that the Sup35NM prion remains disordered when fused to the I27 carrier module (the CD spectrum of I27+Sup35NM closely resembles the sum of I27 and Sup35NM spectra weighted by the relative contribution of their respective sequences to the fusion protein). Whereas the spectrum at 5 d (inset) shows features indicating that RC conformations are still predominant, spectral analysis reveals a significant increase in β-structure (right panel). Remarkably, the CD spectrum at 5 d has lost the majority of its intensity, possibly because of oligomerization and the formation of insoluble aggregates (see [C]). QBP1 (20 µM) appears to suppress these oligomerization processes and to reduce the formation of β-structure. Apparently, I27+Sup35NM acquires more β-structure than the isolated Sup35NM. This could be attributed to the positioning of the NP termini in close proximity when it is nested within I27, which might encourage the formation of contacts. (B) The 1H NMR spectra of I27-MCS (black), Sup35NM (blue), and I27+Sup35NM (red) are shown on the left with an expanded view of the HN resonances on the right. The line broadening of the I27+Sup35NM spectrum is more evident than that observed for the other carrier-guest proteins examined (Figures S2B, S3B, S4B, S5B), probably due to this being the largest guest studied (which is in a RC conformation) and/or the formation of the oligomers and aggregates inferred by CD (A). Therefore, to confirm that the native structure of the I27 carrier is maintained, a 2D 1H NOESY spectrum was recorded. In this spectrum, signals arising from the packing of aliphatic and aromatic groups in the native hydrophobic core of I27 were observed and rigorously assigned, thus allowing us to conclude that the native conformation of I27 is still compati [file pbio.1001335.s006.tif]

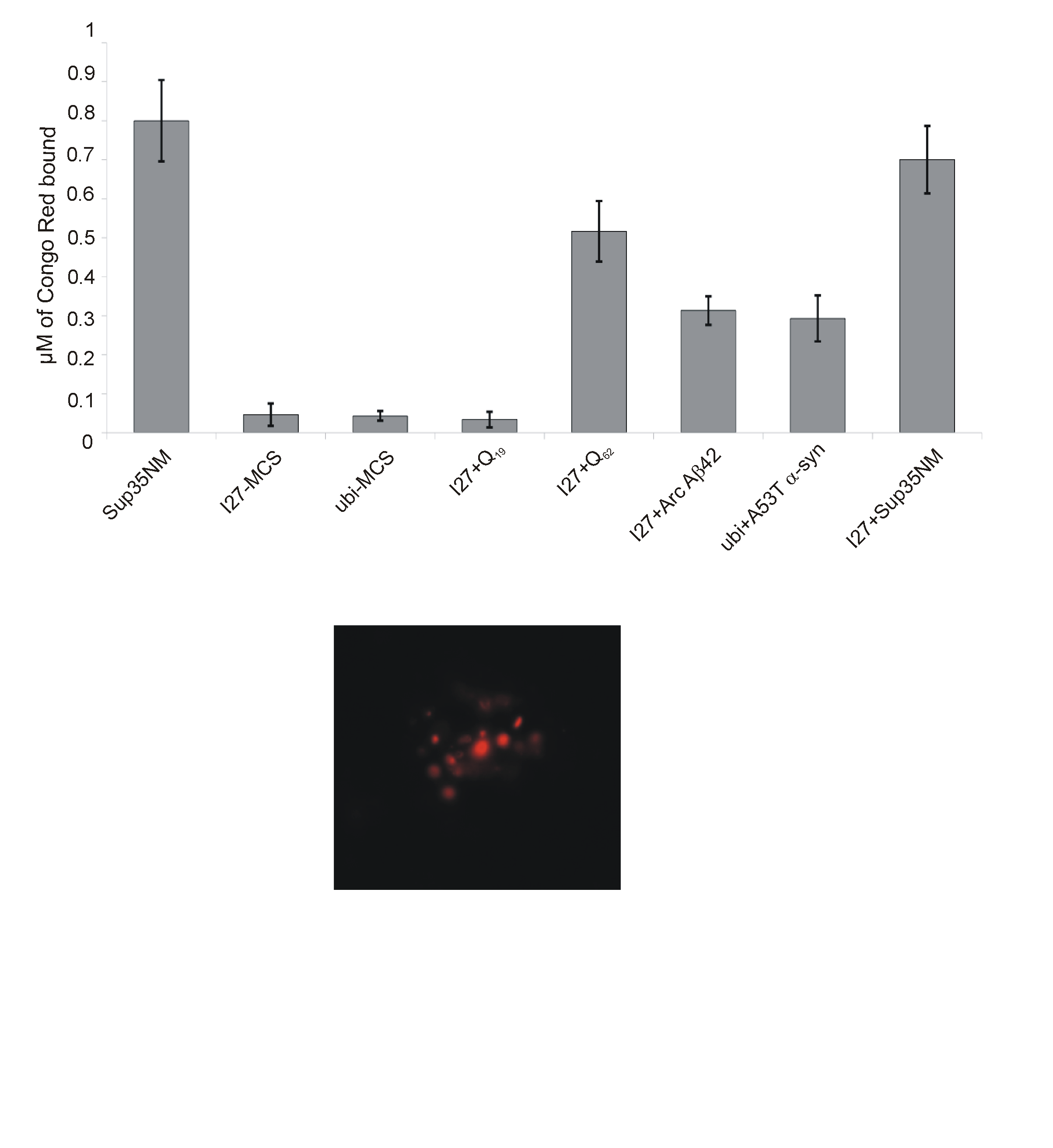

Supplement: Figure S7 — Congo Red binding assay. Concentration (µM) of Congo Red dye bound to the amyloid aggregates formed by representative NPs (upper panel), as calculated through the bathochromic and hyperchromic shifts undergone by the dye upon specific binding to amyloid aggregates [39]. Isolated Sup35NM was used as a positive control for these measurements. The bottom panel shows an optical fluorescence micrograph taken with a 63× objective showing amyloid aggregates formed by I27+Sup35NM bound to Congo Red. (TIF) [file pbio.1001335.s007.tif]

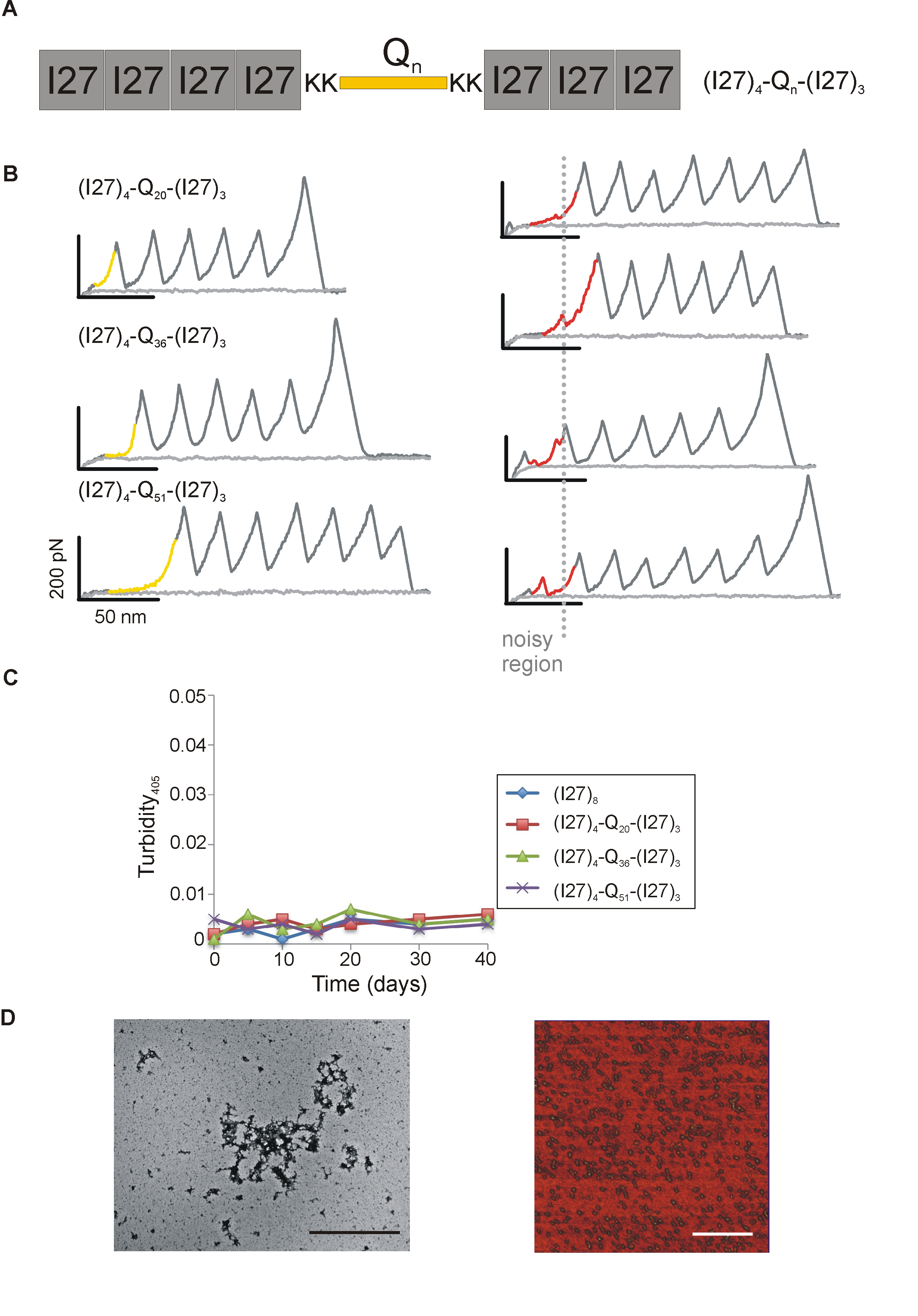

Supplement: Figure S8 — Standard heteropolyprotein approach (in series) for the nanomechanical analysis of polyQ tracts. (A) Cartoon representing the heteropolyprotein strategy used in this analysis: (I27)4-Qn-(I27)3. This strategy is conceptually similar to that recently used for the nanomechanical analysis of α-synuclein [26]. Two lysine residues were placed flanking the polyQ tracts to enhance the solubility of the heteropolyproteins [67]. (B) SMFS spectra of the indicated polyproteins. The left traces show typical recordings of a NM conformation for the polyQ tracts (in orange) while several possible M conformers of the protein (I27)4-Q51-(I27)3 are shown on the right (in red). However, as these force peaks lie in the frequently contaminated proximal region of the recordings, one cannot unequivocally demonstrate that they originate from true M conformers of the NP monomer [45]. (C) Turbidimetry of (I27)4-Qn-(I27)3 samples, monitored by absorbance at 405 nm over the incubation period at 37°C. The lack of turbidity suggests an inability to aggregate [5]. (D) TEM image of (I27)4-Q51-(I27)3 after 28 d at 37°C (left). Proteins were used at a concentration of 10 µM and no fibers were observed. The image was acquired at 30,000× and the scale bar corresponds to 1 µm. AFM image of (I27)4-Q51-(I27)3 incubated for 30 d at 37°C (right) in which no amyloid fibers were observed. The image was acquired using the dynamic mode in liquid conditions as described previously [66]. Scale bar corresponds to 0.5 µm. (TIF) [file pbio.1001335.s008.tif]

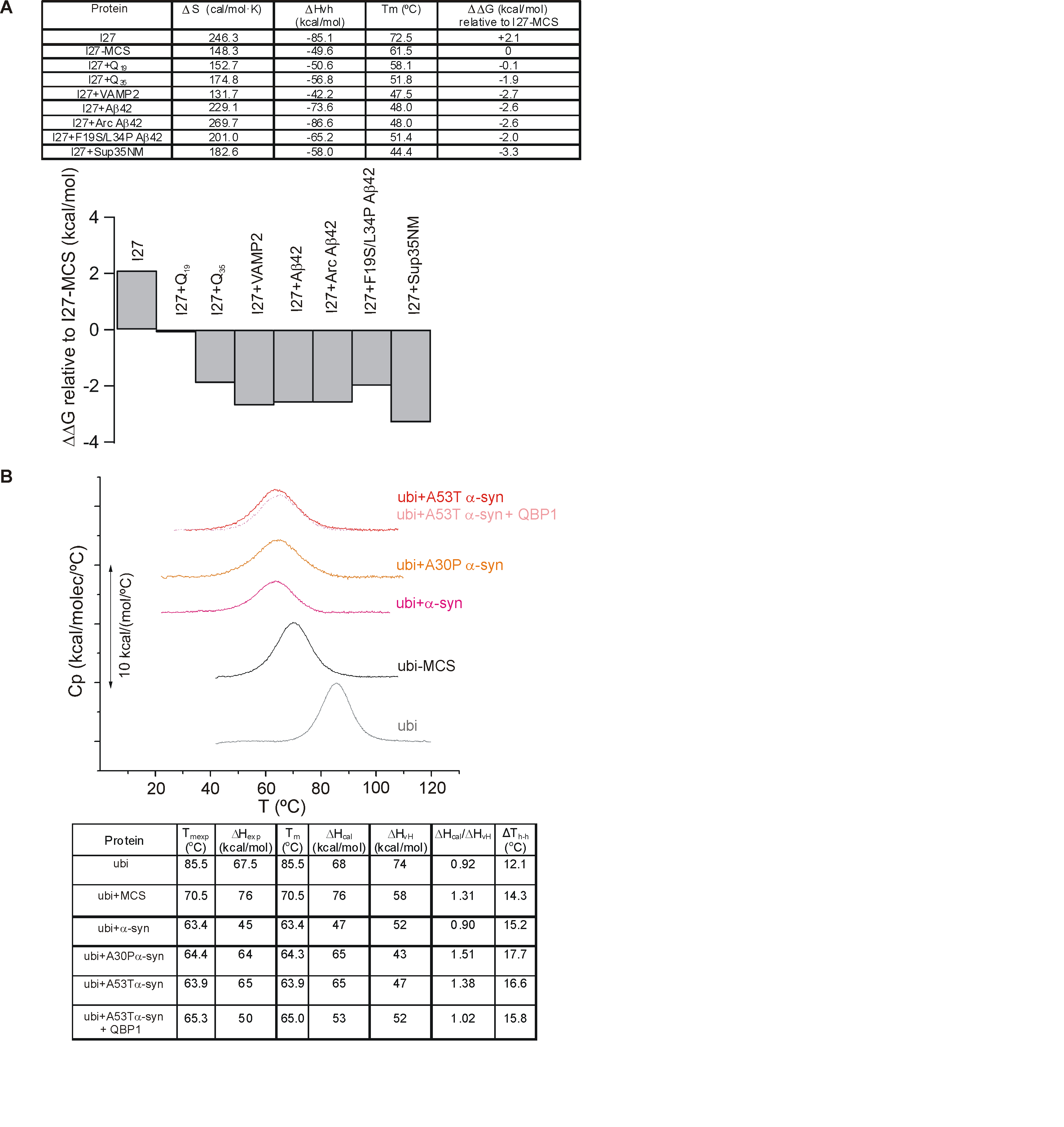

Supplement: Figure S9 — Thermodynamic stability of the carrier protein upon insertion of the guest. (A) Thermal denaturation parameters derived by CD spectroscopy assuming a two-state (N↔D) equilibrium model with pre- and post-transition baselines linearly depending upon temperature [68]. The half transition temperature (T m) and the van't Hoff enthalpy change (ΔH vH) were directly derived from fitting while the entropy change of unfolding (ΔS) was calculated using the Gibbs equation [69]. The change in the conformational stability relative to the I27-MCS variant (ΔΔG) was estimated using the following equation:where ΔT m is the difference in the T m value for a variant relative to I27-MCS [70]. The ΔS value used was 193 cal·mol−1·K−1, which is the mean value for all the variants. (B) Differential scanning calorimetry (DSC) curves of indicated proteins and summary of the denaturation parameters (T mexp is the T-value at Cp max; ΔH exp the enthalpy change estimated by integration of the DSC curve; T m, ΔH cal, and ΔH vH the half transition temperature, and the calorimetric and the van't Hoff enthalpy changes estimated from the endotherm deconvolution; and ΔT h–h the curve width at half-height). Insertion of A30P or A53T α-synuclein into the ubiquitin carrier significantly increases the denaturation enthalpy when compared to insertion of the wt α-synuclein. Moreover, the ratio between ΔH cal and ΔH vH is above unity, suggesting a tendency towards intermediate formation during unfolding. This effect disappears upon the addition of an excess (50 µM) of QBP1 to ubi+A53T α-synuclein samples, which then unfold following the two-state denaturation model. (TIF) [file pbio.1001335.s009.tif]

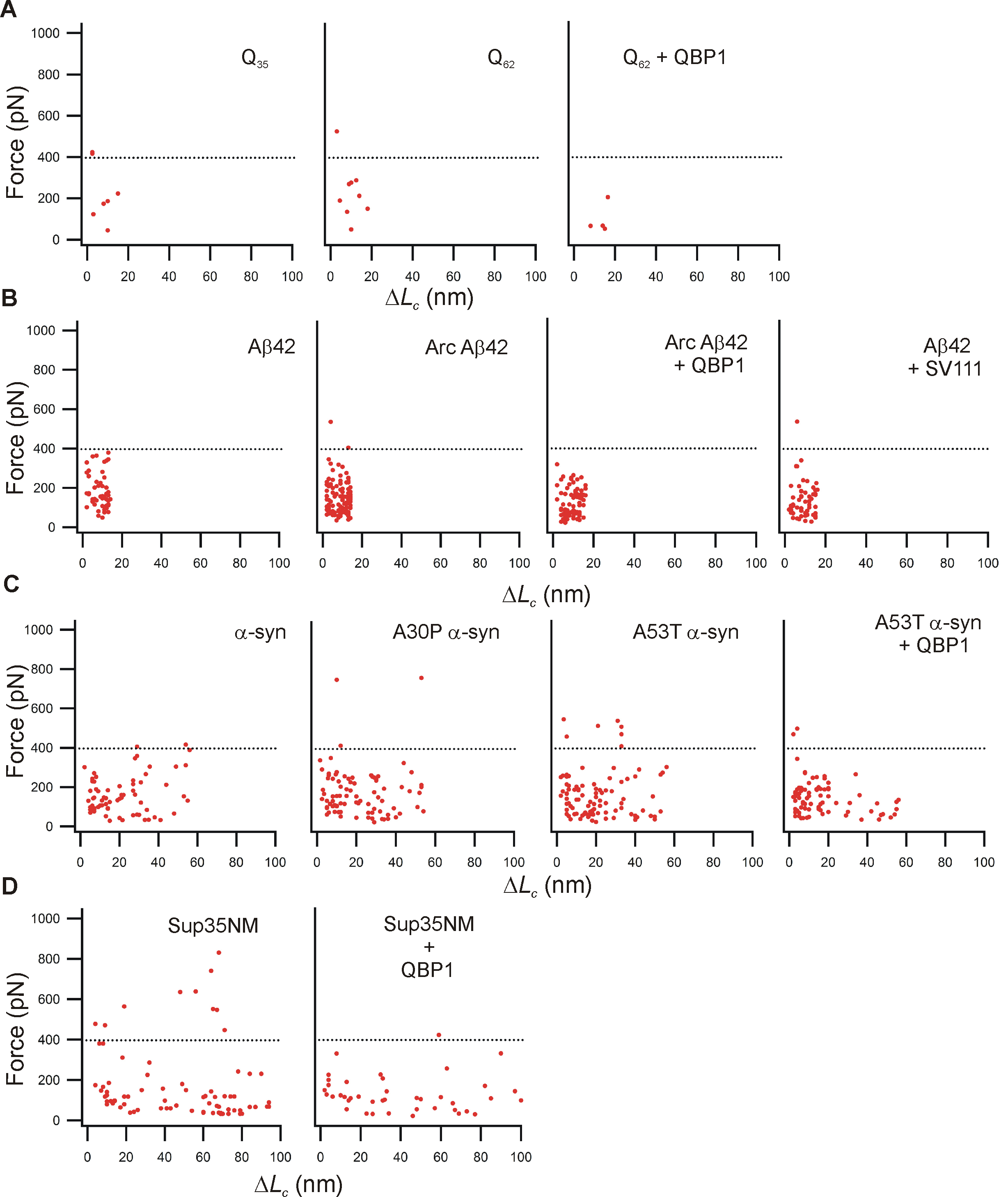

Supplement: Figure S10 — Lack of correlation between F and Δ L c. Scatter plots show that there is no correlation between F and ΔL c values for: (A) polyQ tracts, (B) Aβ42, (C) α-synuclein, and (D) Sup35NM proteins. No clustering in these plots was observed for any of the NPs studied, strongly suggesting that no specific regions in the NPs mediate their mechanical stability. Furthermore, treatment of samples with QBP1/SV111 peptides did not result in the loss of any specific population of ΔL c data points. In principle, this observation prevents us from extracting any structural information regarding the mechanism of QBP1 and SV111 binding to NPs. (TIF) [file pbio.1001335.s010.tif]

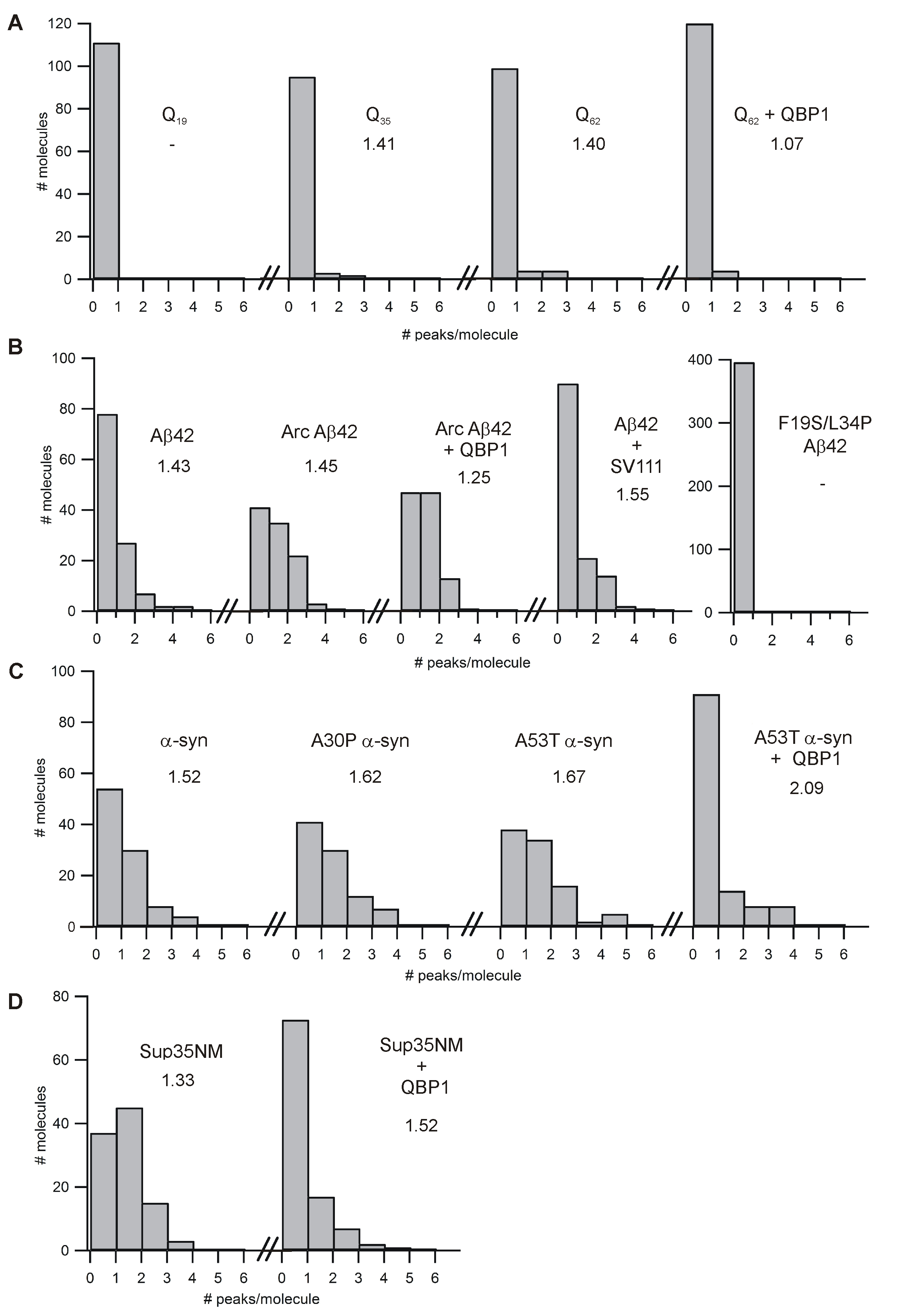

Supplement: Figure S11 — Effect of familial-disease mutations and QBP1 on the number of structured regions per molecule. The average number of force peaks per molecule is shown for: (A) polyQ tracts; (B) Aβ42; (C) α-synuclein; and (D) Sup35NM proteins. NM conformations correspond to 0 force peaks. These events are excluded from the average calculations shown in the figure. The familial-disease mutations increase the number of force peaks per molecule, suggesting that they increase the propensity to acquire M structures. By contrast, QBP1 reduces this number for Q62 and Arc Aβ42 but not for A53T α-synuclein or Sup35NM. Additional controls are required to test if these average differences are significant. SV111 does not reduce this number in Aβ42. (TIF) [file pbio.1001335.s011.tif]

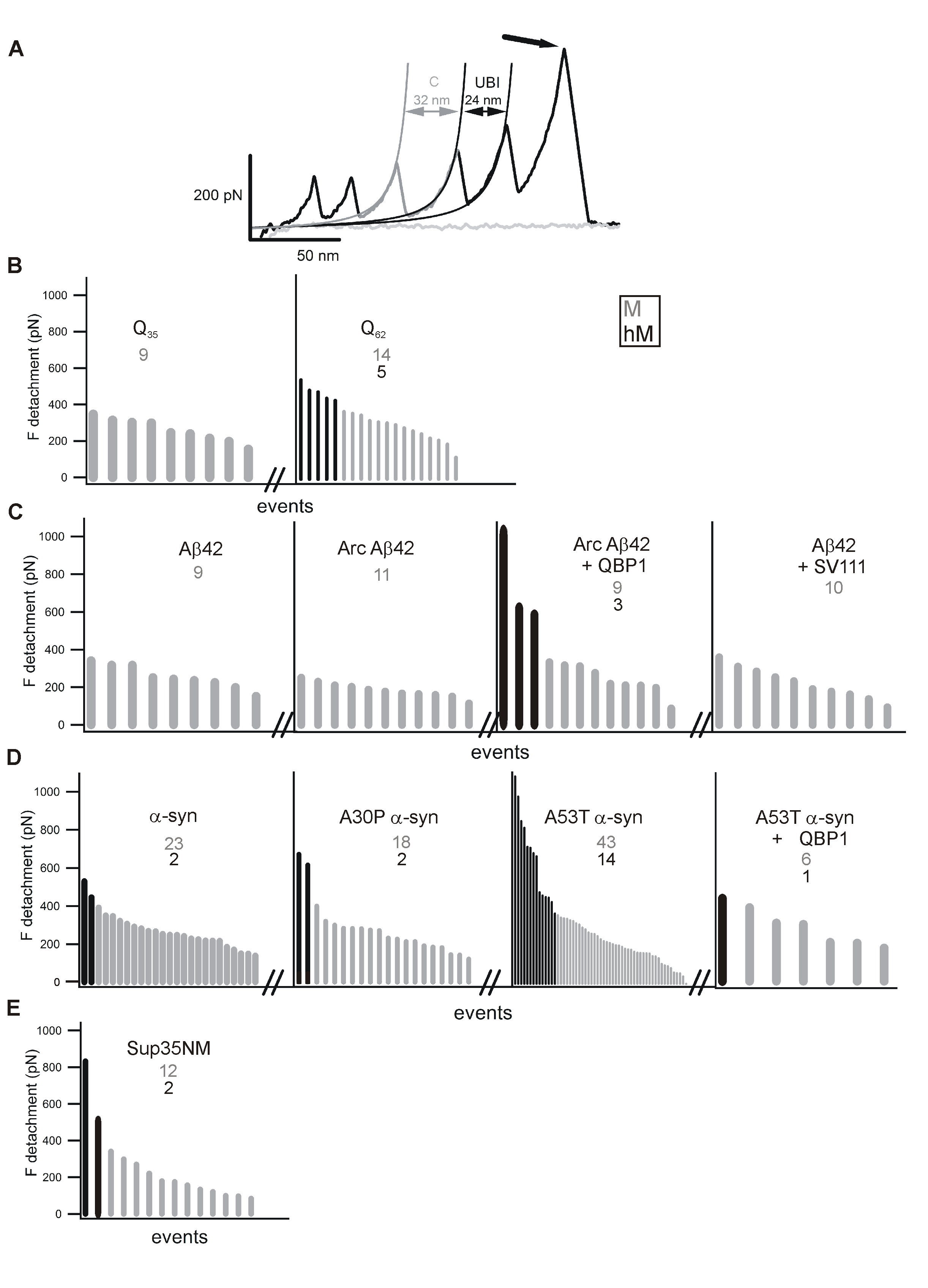

Supplement: Figure S12 — Putative additional hM events. (A) Example of an event considered as a putative hM. In gray we show the force peak due to the unfolding of the carrier module (C) and a region of the NP that is shorter than its full length. The ΔL c value associated with this force peak is larger than that of a normal ubiquitin repeat but not as large as that expected if the entire NP was stretched. To explain these frequent events, we assume that the polyprotein attachments (either from the tip or the substrate) were not maintained until the end of the experiment (i.e., the polyprotein molecule may have detached before reaching the forces required to fully unfold the region trapped [28]). Therefore, it is reasonable to assume that its mechanical stability would be higher than the force of detachment (last peak in the spectrum, indicated by the arrow). (B–E) Force of detachment values for the recordings considered to contain putative additional events for polyQ tracts, Aβ42, α-synuclein, and Sup35NM, respectively. Putative hM events (with detachment forces ≥400 pN) are shown in black. This analysis suggests that the population of hM conformers observed directly in our study represents an under-estimate of the actual propensity of the NPs to form these conformers. Thus, it appears that SMFS experiments miss a considerable fraction of hM conformers owing to the experimental approach, which seems to filter out several hM events. These data were not included in our sample size (n). In (B–E) each vertical bar represents a single event (one molecule). Note that Q62+QBP1 and Sup35NM+QBP1 yielded no putative hM events and therefore are not represented here. (TIF) [file pbio.1001335.s012.tif]

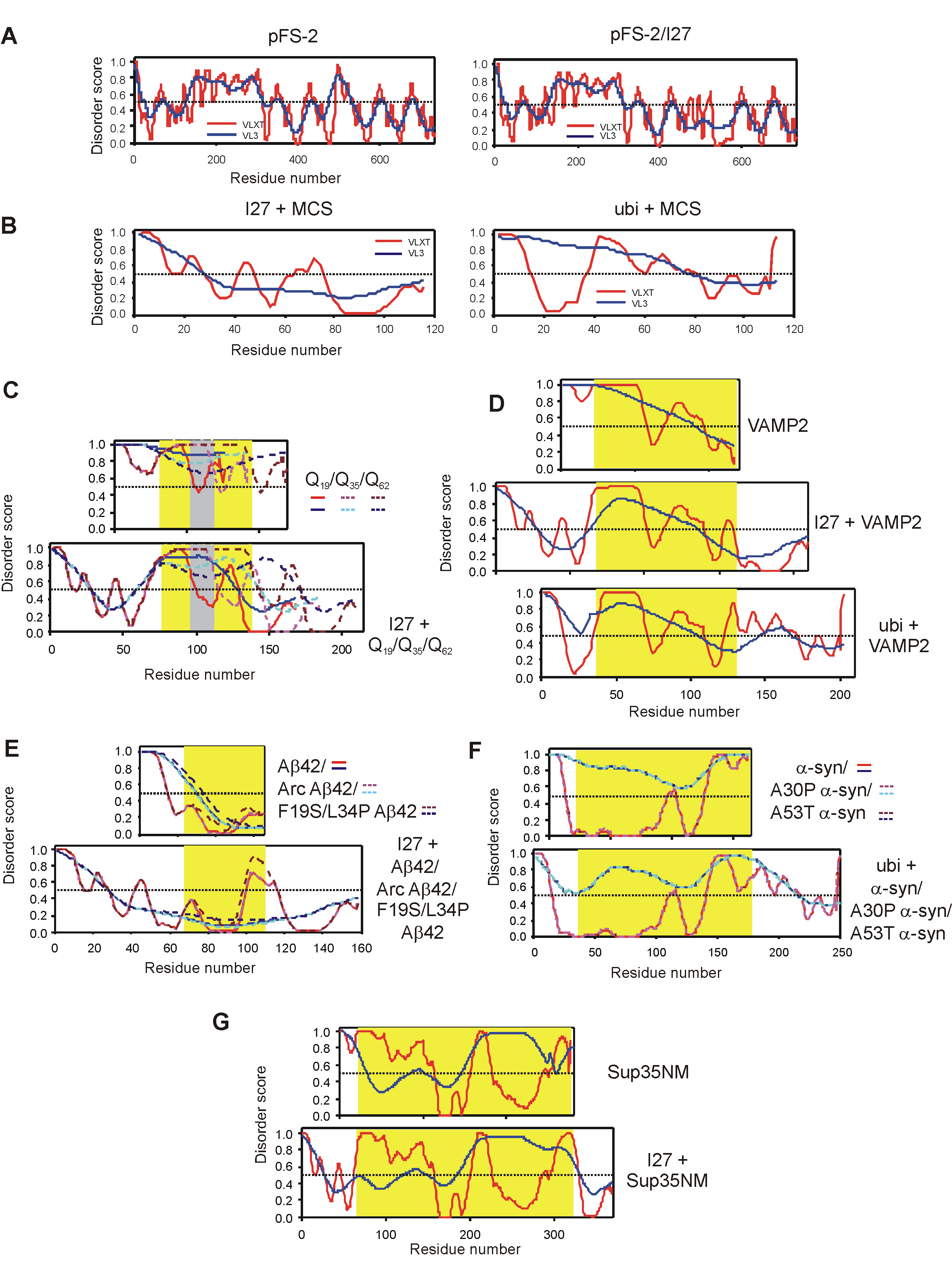

Supplement: Figure S13 — Intrinsic disorder predictions for the fusion polyproteins/proteins used in the study. PONDR values below 0.5 indicate a tendency to form ordered structures, while those over 0.5 indicate a tendency for disorder. PONDR VLXT and PONDR VL3 data are shown as red and blue lines, respectively. (A) pFS-2 (left) and pFS-2/I27 (I27 as the carrier protein, right): these predictions show a reasonable correlation with the proteins contained in the pFS polyproteins, where the disordered region corresponds to the N2B stretch while the ordered regions correspond to the I27/ubi repeats (Figure 1A, [30]). (B) I27-MCS (left) and ubi-MCS (right). (C–G) NPs/VAMP2 alone (top, guest) and carrier-guest proteins (bottom), as indicated in the figure. The corresponding guest sequences are shaded yellow in all plots. Scales for guest and carrier/guest predictions were normalized such that the region of interest is same length in both plots. The ends of the regions sharing common sequences were located in the same x position to facilitate data comparison of guest and carrier/guest proteins. As Q35 and Q62 start at the same position in both constructs ([C] top and bottom) but are of different lengths, the corresponding NP shading starts at the same position but extends differently. The first yellow shaded area corresponds to Q19, while Q35 extends to the end of the grey shaded region and Q62 to the end of the second yellow shaded zone. The non-shaded region in the guest plots corresponds to different residues in the protein used, which do not belong to the guest sequence (i.e., purification tags and residues added in polyQ expansions [71]). PONDR VLXT and VL3 use different parameters and window sizes, and were trained on very different datasets; as a result, their predictions differ considerably, especially for longer NPs ([F] α-synuclein and [G] Sup35NM). Nonetheless, all the NPs (except polyQ tracts, as discussed in Text S1) contain segments that are predicted to be ordered, in agreement wit [file pbio.1001335.s013.tif]
