# Supplementary material for: Common Features at the Start of the Neurodegeneration Cascade
Source: PLoS Biol. 2012 May 29;10(5):e1001335. doi: 10.1371/journal.pbio.1001335 (PMC3362641; doi:10.1371/journal.pbio.1001335)
Supplement: Table S1 — Summary of controls for structure, aggregation, and fibrillogenesis of the NP. Experiments were carried out with the carrier-guest proteins alone (unless otherwise stated). ND, not determined; +, positive result; −, negative result. Abbreviations for the techniques are provided in the text. iAFM, imaging AFM. QBP1 and SV111 are inhibitors referred to in the main text and described in the Text S1 supplementary methods section. (DOC) [file pbio.1001335.s014.doc]

| **Protein** | | | **Aggregation test (Turbidity / Congo Red)** | **Structural analysis (CD / NMR)** | **Fibers (TEM / iAFM)** |
| --- | --- | --- | --- | --- | --- |
| **PolyQ** | **Q_19_** | | - /- | +/+ | -/ND (- with QBP1) |
|  | **Q_35_** | | +/ND | +/+ | +/ND (- with QBP1) |
|  | **Q_62_** | **-QBP1** | +/+ | ND/ND | +/ND |
|  |  | **+QBP1** | -/ND | ND/ND | -/ND |
| **VAMP2** | **I27-VAMP2** | | -/ND | +/+ | -/ND |
|  | **Ubi-VAMP2** | | -/ND | +/+ | -/ND |
| **A*β*** | **A*β*42** | **-SV111** | +/ND | +/+ | +/ND |
|  |  | **+SV111** | -/ND | +/ND | -/ND |
|  | **Arc A*β*42** | **-QBP1** | +/+ | +/+ | +/ND |
|  |  | **+QBP1** | +/ND | +/ND | +/ND |
|  | **F19S/L34P A*β*42** | | -/ND | +/+ | -/ND |
| ***α*-syn** | **wt** | | +/ND | +/+ | +/ND |
|  | **A30P** | | +/ND | +/+ | +/ND |
|  | **A53T** | **-QBP1** | +/+ | +/+ | +/+ |
|  |  | **+QBP1** | +/ND (reduced) | +/ND | -/ND |
| **Sup35NM** | **-QBP1** | | +/+ | +/+ | +/+ (+ in pFS-2) |
|  | **+QBP1** | | +/ND (reduced) | +/ND | +/ND (highly reduced) |
