# Supplementary material for: Common Features at the Start of the Neurodegeneration Cascade
Source: PLoS Biol. 2012 May 29;10(5):e1001335. doi: 10.1371/journal.pbio.1001335 (PMC3362641; doi:10.1371/journal.pbio.1001335)
Supplement: Table S2 — Mechanical stability ( F ) of the carrier proteins upon insertion of guest proteins. The F-values of the carriers reveal a significant decrease in their mechanical stability upon insertion of the guest proteins (both in the NM or M conformations) when compared to the carriers alone (204±26 pN for I27 [72] and 203±35 for Ubi [73], at comparable pulling speeds). This appears to indicate that the guest protein affects the mechanical stability of the carriers. Considering that our criteria for selection of SMFS recordings imply the observation of the force peak for the carrier unfolding, our SMFS data may be an underestimation (Table 1). SD, standard deviation. (DOC) [file pbio.1001335.s015.doc]

| **Carrier** | **Guest** | **n** | ***F* (x ± SD) (pN)** |
| --- | --- | --- | --- |
| **I27** | Q_19_ | NM (n= 111) | 149 ± 62 |
|  | Q_35_ | NM (n= 95) | 156 ± 51 |
|  |  | M (n= 5) | 180 ± 11 |
|  | Q_62_ | NM (n= 99) | 200 ± 36 |
|  |  | M (n= 8) | 212 ± 36 |
|  | Q_62_ + QBP1 | NM (n= 120) | 169 ± 89 |
|  |  | M (n= 4) | 137 ± 10 |
|  | A*β*42 | NM (n= 78) | 128 ± 64 |
|  |  | M (n= 38) | 125 ± 76 |
|  | Arc A*β*42 | NM (n=39) | 119 ± 69 |
|  |  | M (n=63) | 116 ± 96 |
|  | Arc A*β*42 + QBP1 | NM (n=46) | 128 ± 51 |
|  |  | M (n=62) | 110 ± 71 |
|  | A*β*42 + SV111 | NM (n=89) | 133 ± 77 |
|  |  | M (n=39) | 156 ± 78 |
|  | F19S/L34P A*β*42 | NM (n=396) | 174 ± 43 |
|  |  |  |  |
|  | Sup35NM | NM (n=37) | 146 ± 30 |
|  |  | M (n=62) | 141 ± 79 |
|  | Sup35NM + QBP1 | NM (n=73) | 125 ± 78 |
|  |  | M (n=27) | 122 ± 61 |
|  | VAMP2 | NM (n=188) | 152 ± 50 |
|  |  |  |  |
| **ubi** | VAMP2 | NM (n=234) | 149 ± 48 |
|  |  |  |  |
|  | *α*-syn | NM (n=54) | 149 ± 115 |
|  |  | M (n=44) | 155 ± 35 |
|  | A30P *α*-syn | NM (n=41) | 161 ± 90 |
|  |  | M (n=51) | 115 ± 91 |
|  | A53T *α*-syn | NM (n=38) | 117 ± 67 |
|  |  | M (n=58) | 121 ± 94 |
|  | A53T *α*-syn + QBP1 | NM (n=224) | 182 ± 56 |
|  |  | M (n=35) | 187 ± 98 |

**-**
