# Supplementary material for: Common Features at the Start of the Neurodegeneration Cascade
Source: PLoS Biol. 2012 May 29;10(5):e1001335. doi: 10.1371/journal.pbio.1001335 (PMC3362641; doi:10.1371/journal.pbio.1001335)
Supplement: Table S3 — Putative M events and number of events per molecule. # peaks/molecule, average number of M regions (force peaks) per molecule. The values reported are calculated considering only M conformers, excluding those monomers exhibiting a NM conformation (which result in 0 force peaks; see Figure S11). Error calculations are explained in the Text S1 supplementary methods. (DOC) [file pbio.1001335.s016.doc]

| **Protein** | | | **M (%)** | **Putative M** | **hM (%)** | **Putative hM** | **# Peaks / molecule** |
| --- | --- | --- | --- | --- | --- | --- | --- |
| **PolyQ** | **Q_19_** | | 0 | **0** | 0 | **0** | **0** |
|  | **Q_35_** | | 5.0 (4) | **9** | 1 | **0** | **1.41** |
|  | **Q_62_** | **-QBP1** | 7.5 (5) | **19** | 2.8 | **5** | **1.4** |
|  |  | **+QBP1** | 3.3 (3) | **0** | 0 | **0** | **1.07** |
| **A*β*** | **A*β*42** | **-SV111** | 32.8 (9) | **9** | 0 | **0** | **1.43** |
|  |  | **+SV111** | 30.2 (8) | **10** | 0.8 | **0** | **1.55** |
|  | **Arc A*β*42** | **-QBP1** | 62.1 (10) | **11** | 1 | **0** | **1.45** |
|  |  | **+QBP1** | 57.4 (9) | **12** | 0 | **3** | **1.25** |
|  | **F19S/L34P A*β*42** | | 0 | **0** | 0 | **0** | **0** |
| ***α*-syn** | **wt** | | 44.9 (10) | **25** | 2 ± 1 | **2** | **1.52** |
|  | **A30P** | | 59.0 (10) | **20** | 3.3 | **2** | **1.62** |
|  | **A53T** | **-QBP1** | 60.4 (10) | **57** | 5.2 ± 1.0 | **14** | **1.67** |
|  |  | **+QBP1** | 13.5 (4) | **7** | 0.4 | **1** | **2.09** |
| **Sup35NM** | **-QBP1** | | 64.0 (10) | **14** | 8 | **2** | **1.33** |
|  | **+QBP1** | | 27.0 (9) | **0** | 1 | **0** | **1.52** |
