# Supplementary material for: Common Features at the Start of the Neurodegeneration Cascade
Source: PLoS Biol. 2012 May 29;10(5):e1001335. doi: 10.1371/journal.pbio.1001335 (PMC3362641; doi:10.1371/journal.pbio.1001335)
Supplement: Table S4 — Summary of the oligonucleotides used to design the I27 carrier module and for the cloning of NPs. The restriction sites introduced by PCR into the amplified sequences are highlighted in italics. Mutagenic oligonucleotides are indicated by (Mut) and the mutations introduced are highlighted in italics and bold. The extra sequences added to the terminus of each restriction site were chosen on the basis of recommendations from New England Biolabs, which enhance the digestion efficiency of linear DNA sequences. All oligonucleotides used were purchased from Sigma-Aldrich. (DOC) [file pbio.1001335.s017.doc]

| **Name (Mut: mutagenic)** | **Sequence 5→3** |
| --- | --- |
| I27-MCS 5’ (Mut) | GAAAGGACAGCCTTTGGCA***ACCGGTTCATCCCGGG***GCTTCCCCTGACTGTGAAATC |
| I27-MCS 3’ (Mut) | GATTTCACAGTCAGGGGAAGC***CCCGGGATGAACCGGT***TGCCAAAGGCTGTCCTTTC |
| pFS-2+*α*-Syn 5’ | *ACCGGT*ATGGATGTATTCATGAAAGGACTTTCAAAGGC |
| pFS-2+*α*-Syn 3’ | *CCCGGG*GGCTTCAGGTTCGTAGTCTTGATACCCTTCC |
| A30P *α*-Syn 5’ (Mut) | GGTGTGGCAGAAGCA***CCA***GGAAAGACAAAAGAGG |
| A30P *α*-Syn 3’ (Mut) | CCTCTTTTGTCTTTCCT***GG***TGCTTCTGCCACACC |
| A53T *α*-Syn 5’ (Mut) | GTGGTGCATGGTGTG***ACA***ACAGTGGCTGAGAAG |
| A53T *α*-Syn 3´ (Mut) | CTTCTCAGCCACTGT***TGT***CACACCATGCACCAC |
| ubi+α-Syn, ubi+A30P *α*-Syn, ubi+A53T *α*-Syn 5’ | GAATTCGGCTT*GCTAGC*ATGCAAATC |
| ubi+*α*-Syn, ubi+A30P *α*-Syn, ubi+A53T *α*-Syn 3’ | GAATTCGGCTT*GTCGAC*TCATCACCCACCTCTGAGAC |
| *α*-Syn, A30P *α*-Syn,  A53T *α*-Syn 5’ | *GCTAGC*ATGGATGTATTCATGAAAGG |
| *α*-Syn, A30P *α*-Syn,  A53T *α*-Syn 3’ | *GTCGAC*TCATCAGCTTCAGGTTCGTAGTCTTG |
| pFS-2+Q_n_ 5’ | atc*accggt*ATGGTTTCCACCCATCACCATCACCACCAGCAAC |
| pFS-2+Q_n_ 3’ | tat*cccggg*CGGCGGACCAGAGTTACCGTGATGCTGCTGC |
| pFS-2+VAMP2 5’ | ccaa*ACCGGT* atgtcggctaccgctgccaccg |
| pFS-2+VAMP2 3’ | tcc*CCCGGG*cttgaggtttttccaccag |
| pFS-2+Sup35NM 5’ | *ACCGGT*ATGTCGGATTCAAACCAAGGC |
| pFS-2+Sup35NM 3’ | *CCCGGG*ATCGTTAACAACTTCGTCATCC |
| pFS-2+A*β*42 5’ | *ACCGGT*GATGCAGAATTCCGACATGACTCAGG |
| pFS-2+A*β*42 3’ | *CCCGGG*CGCTATGACAACACCGCCCACC |
| Arc A*β*42 5’ (Mut) | GGTGTTCTTTGCA***gga***GATGTGGGTTCAAACAAAGG |
| Arc A*β*42 3’ (Mut) | CCTTTGTTTGAACCCACATC***tcc***TGCAAAGAACACC |
| F19S A*β*42 5’ (Mut) | CATCATCAAAAATTGGTGT***C***CTTTGCAGAAGATGTGGG |
| F19S A*β*42 3’ (Mut) | CCCACATCTTCTGCAAAG***G***ACACCAATTTTTGATGATG |
| L34P A*β*42 5’ (Mut) | GGTGCAATCATTGGACCCATGGTG***G***GCGGTGTTGTC |
| L34P A*β*42 3’ (Mut) | GACAACACCGC***C***CACCATGGGTCCAATGATTGCACC |
| I27-MCS (Δ4) 5’ (Mut) | GGACAGCCTTTGGCAACCGGT*(****Δ4****)*CCCGGGGCTTCCCC |
| I27-MCS (Δ4) 3’ (Mut) | GGGGAAGCCCCGGG*(****Δ4****)*ACCGGTTGCCAAAGGCTGTCC |
| VamP2 5’ | CTA*GCTAGC*atgtcggctaccgctgccaccg |
| Vamp2 3’ | CCG *CTCGAGctacta* cttgaggtttttccaccag |
| Sup35NM 5’ | CTA*GCTAGC*ATGTCGGATTCAAACCAAGGC |
| Sup35NM 3’ | CCG*CTCGAG* CTACTAATCGTTAACAACTTCGTCATCC |
| I27-MCS (Δ4), I27+Q_n_,  I27+ A*β*42, I27+Arc A*β*42,  I27+VAMP2,  I27+ F19S/L34P A*β*42, I27+Sup35NM 5’ | CTA*GCTAGC* ctaatagaagtggaGaagcctc |
| I27-MCS (Δ4), I27+Q_n_,  I27+ A*β*42, I27+Arc A*β*42,  I27+VAMP2,  I27+ F19S/L34P A*β*42, I27+Sup35NM 3’ | CCG*CTCGAG* CTACTACAATTCTTTCACTTTCAGATTGGC |
| Q_20_ 5’ | *CTAGC*AAGAAACAGCAACAACAGCAGCAGCAACAGCAACAACAACAGCAACAGCAGCAACAACAACAGCAGAAGAAA*T* |
| Q_20_ 3’ | *CTAGA*TTTCTTCTGCTGTTGTTGTTGCTGCTGTTGCTGTTGTTGTTGCTGTTGCTGCTGCTGTTGTTGCTGTTTCTT*G* |
| Q_30_ 5’ | *CTAGC*AAGAAACAGCAACAACAGCAGCAGCAACAGCAACAACAACAGCAACAGCAGCAACAACAACAGCAGCAGCAACAACAGCAGCAGCAACAGCAACAGAAGAAA*T* |
| Q_30_ 3’ | *CTAGA*TTTCTTCTGTTGCTGTTGCTGCTGCTGTTGTTGCTGCTGCTGTTGTTGTTGCTGCTGTTGCTGTTGTTGTTGCTGTTGCTGCTGCTGTTGTTGCTGTTTCTT*G* |
| Q_30_ 5’ | *CTAGC*AAGAAACAGCAACAACAGCAGCAGCAACAGCAACAACAACAGCAGCAGCAACAGCAGCAACAACAGCAACAGCAACAACAACAGCAACAGCAACAG |
| Q_30_ 3’ | *CTG*TTGCTGTTGCTGTTGTTGTTGCTGTTGCTGTTGTTGCTGCTGTTGCTGCTGCTGTTGTTGTTGCTGTTGCTGCTGCTGTTGTTGCTGTTTCTTG |
| Q_20_ 5’ | CAGCAACAGCAGCAACAACAACAGCAGCAACAGCAGCAACAACAGCAACAGCAACAACAGCAGAAGAAA*TCTAGA* |
| Q_20_ 3’ | *TCTAGA*TTTCTTCTGCTGTTGTTGCTGTTGCTGTTGTTGCTGCTGTTGCTGCTGTTGTTGTTGCTGCTGTTG*CTG* |
| Q_6_ 5’ | CAGCAACAGCAGCAACAGAAGAAA*TCTAGA* |
| Q_6_ 3’ | *TCTAGA*TTTCTTCTGTTGCTGCTGTTG*CTG* |
