# Supplementary material for: Common Features at the Start of the Neurodegeneration Cascade
Source: PLoS Biol. 2012 May 29;10(5):e1001335. doi: 10.1371/journal.pbio.1001335 (PMC3362641; doi:10.1371/journal.pbio.1001335)
Supplement: Text S1 — Supplementary results, discussion, methods, and references. (DOC) [file pbio.1001335.s018.doc]

**Text S1**

**CONTENTS**

1. Supplementary results

A. Unequivocal single-molecule identification

B. Preserved structure and behavior of NPs

C. Measuring intramolecular interactions in NPs

2. Supplementary discussion

A. On the possible existence of preferential conformations and the mechanism of

conformer formation

B. On the possible nature of the mechanical conformers

3. Supplementary methods

4. Supplementary references

**1. Suplementary results**

**A. Unequivocal single-molecule identification**

In standard SMFS, the use of polyproteins flanking (in series) the protein of interest is a necessary but insufficient condition for single-molecule identification. To this end, a constant is typically used as an additional single-molecule fingerprint: the length released after unfolding (Δ*L_c_*, see **Supplementary Methods**). Previous SMFS experiments on fibrillogenic IDPs, including NPs, either failed to obtain direct information on the protein of interest (polyQs [1]), or were hampered by the possible existence of contaminating noise (*α*-synuclein [2,3]; tau [4]). This was due to the fact that selected data included the proximal region of the SMFS recordings, known to be prone to contamination with unwanted interactions, including detachments and multiple molecule events [5]. Indeed, although non-fibrillogenic IDPs have already been analyzed unequivocally by SMFS and found to be featureless (titin PEVK [6] and titin N2B [7]), fibrillogenic proteins like NPs pose a challenge as they appear to have significant mechanical polymorphisms, which include low-mechanostable species [2,3,4]. Considering that the force peaks in the force-extension recordings are typically ordered from lower to higher values, the fact that the mechanical stability of most of these conformers may be below that of the marker means that the signal from the NP will be collected in the proximal (usually contaminated) region of the recordings. As NPs are not featureless but produce force peaks with variable Δ*L_c_* values, the use of polyproteins combined with the Δ*L_c_* is not yet sufficient for unequivocal single-molecule identification. In fact, we used this standard strategy in our preliminary studies constructing heteromeric polyproteins with polyQ tracts of different length (Q_20_, Q_36_ and Q_51_) flanked by a short linker that was followed by the marker polyprotein (titin I27 repeats, **Fig. S8a**). However, as is usual in this technique, most SMFS recordings obtained appeared to be contaminated in the proximal region (**Fig. S8b**, right), similar to those previously reported for *α*-synuclein [2]. Therefore, to obtain clean and robust data, it was clearly necessary to develop a new single-molecule marker strategy.

To overcome this problem, we have developed a new expression vector strategy that enables the unequivocal analysis of fibrillogenic IDPs (pFS-2; [8], **Fig. 1a**). In this strategy, rather than cloning the protein of interest intercalated between polyproteins (mechanically in series [9]) it was introduced within a tolerant loop of a carrier, either the titin I27 module or ubiquitin (both model proteins in protein nanomechanics). This “carrier-guest” protein strategy (*i.e.*, grafting of the NP within the carrier) mechanically protects the protein of interest inside the marker so that its unfolding signal always appears after that of the carrier, far from the noisy proximal region of the recordings (**Fig. 1b**, see **Supplementary Methods** for the details of this strategy and [8]).

**B. Preserved structure and behavior of NPs**

As mentioned, the performance of structural and amyloidogenic controls is a pre-requisite whenever a fusion protein is made using a NP [10-12]. However, the standard approach (mentioned in the previous section, **Fig.S8a**) unfortunately yields fusion polyproteins that neither aggregate, as assessed by 405 nm-turbidometric tests (**Fig. S8c** [13]), nor fibrillate, according to TEM and AFM (**Fig. S8d**). These constructs were similar to those used in a previous study with *α*-synuclein, in which no aggregation or fibrillogenesis controls for the polyprotein under study were included [2]. Indeed, the SMFS recordings of this study were similar to those presented here using the standard strategy (**Fig. S8b**, right). As lysine residues have previously been used successfully to enhance the solubility of monomeric polyQs (electrostatic repulsions were used here to slow the reaction kinetics), we speculate that mobility and steric restrictions in this construct may also prevent the pathological polyQ tracts from acquiring an aggregation-prone conformation. The effects on aggregation of the sequences flanking the polyQ tracts have been widely documented [14,15,16].

Accordingly, we decided to abandon the conventional polyprotein approach and to develop the carrier-guest strategy [8]. As a first control of our new strategy, we characterized the structure, stability, aggregation and amyloidogenesis of the new constructs using a battery of spectrophotometric and structural techniques (**Supplementary Methods**). For technical simplicity, we used the minimal neurotoxic fusion protein in these experiments, engineering only the carrier-guest protein (carrier+NP) without the polyprotein marker encoded in the pFS-2 vector.

For SMFS analysis, it is essential to maintain the integrity of the carrier protein, at least that of the mechanical clamp. As such, we first examined its structure and thermal stability. Our results show that the carrier protein was folded and its structure was essentially maintained (**Figs S2a,b-S6a,b**), although its thermodynamic stability was reduced (**Fig. S9**). Previous studies have reported similar findings: a decreased stability for myoglobin proteins carrying poly-Q grafts [17] and the maintenance of the tertiary fold by RNase A bearing a polyQ graft [10] though no thermodynamic measurements were performed in this case to determine stability changes. The destabilizing effect of introducing a longer loop into a protein has been examined both theoretically and experimentally. It is expected to depend on the type of residue inserted and the structure adopted. For flexible loops of the size of the NPs used in this study, a destabilization of about two kcal/mol is expected, though it could be higher if the NP adopts a stiffer conformation [18]. The predicted destabilization was in the range of the effects observed here by monitoring thermal denaturation using CD and DSC (**Fig S9**).

Importantly, the mechanical stability of the carrier was generally preserved, in agreement with far-UV CD (**Figs. S2a-S6a**, left) and ^1^H monodimensional NMR (**Figs. S2b-S6b**) spectra, albeit in some constructs was noticeably reduced (**Table S2**). Whereas conclusive evidence for fully folded carrier protein was detected in all preparations by 1D and 2D NMR spectroscopy, in some preparations, a significant fraction of carrier protein appeared to be denatured. It should be emphasized that molecules with unfolded carriers would not produce the force peaks expected for the native ones and would have been excluded from our SMFS analysis. The time course for the estimated *β*-structure content was also examined over incubation at 37ºC (0, 2, 5, and 7 days, see **Supplementary Methods**), resulting in the expected behavior (**Figs. S2a-S6a**, right). Furthermore, *α*-synuclein, alone and fused to the carrier protein, showed *α-*helical structure when bound to SDS micelles (**Fig. S5a,** left), as previously described [19].

The aggregation of the nested NPs in the carrier was also monitored by turbidity tests at 405 nm (**Figs. S2c-S6c**) and Congo Red binding assays, which confirmed that their aggregation was amyloidogenic (**Fig. S7**). When *α*-synuclein (wt and both A30P and A53T mutants) was incubated for different periods of time at 37ºC, all protein constructs, starting as a monomer at day 0, aggregated within the expected time frame as assessed by native PAGE gels (stained with Coomassie Brilliant Blue) and by immunoblots under PAGE denaturing conditions using a conformational antibody against oligomers of all NPs (A11 [20]; **data not shown**; see **Supplementary Methods**). The formation of amyloid fibers by the nested NPs was monitored by imaging AFM (**Figs. S5d** and **S6d**) and TEM (**Figs. 2-5** and **S2d-S6d**). For comparison, all these experiments were also performed with isolated constructs of A53T *α*-synuclein and Sup35NM (using the isolated NP, not fused to the carrier; **Figs. S5d** and **S6d**). Finally, as an additional control, TEM and AFM images were also acquired for Sup35NM using the whole neurotoxic polyprotein (pFS-2+Sup35NM), which again showed fiber formation, although at a lower density than that of the carrier-guest protein alone (**Fig. S6d**). All these structural/fibrillogenic controls and those used throughout the remainder of this work are summarized in **Table S1**.

Together, these experimental data lead us to conclude that, using the selected approach, surprisingly, the NPs studied appear to behave similarly when nested in the carrier protein and when isolated, particularly for *α*-synucleins and Sup35NM for which we can compare (for all parameters analyzed) with the isolated monomers. Hence, the carrier protein appears not to affect their amyloidogenic behavior, although it could presumably introduce considerable restrictions in the conformational space of the nested NP. If the N- and C-termini were close together in the structured conformation of an NP, then being nested within the carrier protein will entropically disfavor the unfolded ensemble relative to that conformation by up to 5 kcal/mol (based on destabilization of native proteins upon the reduction of disulfide bonds [21]). Alternatively, the positioning of the NP within the carrier protein will destabilize structured conformations in which the N- and C-termini are distant from each other. Furthermore, the modules flanking the carrier-guest protein in the pFS-2 polyprotein appear not to prevent aggregation of the NPs although they do slow down amyloidogenesis kinetics (**Fig. S6d**).

Finally, as an additional test of the overall integrity of the carrier and guest proteins, and of the behavior of the NPs, their sequences were studied by current order/disorder models (**Fig. S13**). The disorder predictions run with PONDR predictor for the isolated NP and the carrier-NP (with artificial NP neighbours) are comparable, providing further evidence that the carrier-guest strategy does not cause substantial structural alterations in the NP. Disorder predictions are in line with the SMFS behavior of constructs containing *α*-synuclein, A*β*42 and Sup35NM. Thus, these three proteins are predicted to contain regions with significant residual structure, whereas VAMP2 has almost no residual structure. In contrast, polyQ tracts are predicted to be highly disordered in all constructs, regardless of the polyQ length. Nevertheless, it should be noted that this homopolymer tends to adopt a highly compact conformation in aqueous solutions due to its poor solubility in water [22].

**C. Measuring intramolecular interactions in NPs**

Previous SMFS studies of NPs did not provide controls for intramolecular interactions [2,3]. The pFS-2 is a fair strategy to avoid the contamination of SMFS data with non-specific interactions from the proximal region of the AFM. However, other possible interactions must be ruled out before force events can be unequivocally attributed to the unfolding of NP monomers. These include interactions of the NP molecule with the carrier, with the AFM elements (the substrate or the tip) and with other surrounding NP molecules (intermolecular interactions). First, it should be noted that the pFS-2 strategy also ensures that the NP data are collected when the NP is far away from the surface and any possible spurious interactions (with other NP molecules or AFM elements) have most likely been broken at the beginning of the stretching process (*i.e*., before the carrier unfolds). Furthermore, the forces attributed to single rupture events of monomers in amyloid fibers from human prion protein (115 ± 5 pN at 9.4 nN/s) are on average lower than those of the carrier [23], and therefore these intermolecular interactions, if present, would most likely have been broken at the beginning of the stretching process.

Like other amyloidogenic proteins, NPs have an intrinsic propensity to oligomerize [24,25]. Therefore, it is critical to rule out intermolecular interactions in our study. Amyloid aggregation is an autocatalytic process that, like condensation reactions, only occurs above a critical concentration, and it is extremely slow at the initial oligomerization step. The critical concentrations reported, although varying considerably between NPs and for the same NP in different conditions, tend to be in the high micromolar range [26]. In our SMFS experiments, in order to study the nanomechanics of the monomer, we used a quite low concentration of the NP (in the low micromolar range), which should greatly slow down the oligomerization process [26]. Thus, pFS-2 fusion proteins were used in the range of 2-3 *μ*M, the amyloidogenic fraction representing roughly 5-25% of the total length of the polyprotein molecule. Furthermore, it should be noted that, as expected, situating the NP inside the carrier of the pFS-2 fusion protein dramatically reduces the kinetics of amyloid formation. Thus, using a pFS-2 fusion protein concentration above the amyloidogenic critical concentration (10-20 *μ*M, 7-10 times higher than the concentration used in SMFS experiments) for Sup35NM (the NP that was shown to be the most amyloidogenic), the fiber density after one month of incubation at 4ºC was more than an order of magnitude below that of the insert alone or the carrier-guest protein (in the same conditions), both of which were comparable (**Fig. S6d,** panels **6-8**). Based on these facts, we would not expect aggregation in our SMFS conditions. It should be noted also that in our aggregation/amyloidogenesis controls at 37ºC, the amyloidogenic fraction of the carrier-guest proteins (10-40 *μ*M) ranged from 22.1% (I27+Q_35_) to 68.5% (I27+Sup35NM), being 100% for the isolated NPs (*α*-synucleins and Sup35NM).

To further rule out the possibility of intermolecular interactions in our recordings, we used the SV111 peptide, known to inhibit oligomerization in A*β*42 but not the monomer transitions, acting also as a chemical chaperone that reverses oligomerization (EC_50_ = 63 ± 7 *μ*M [27]). Neither the conformational polymorphism nor the conformer frequency of A*β*42 were affected by this peptide (**Fig. 3** and **Table 1**), although it readily inhibited aggregation and fibrillogenesis of our specific fusion protein (**Fig. S4d** and **Table S1**), as seen previously [27]. These results strongly suggest that the behavior observed originates from *bona fide* intramolecular interactions in the NP monomer.

We also considered the possibility that the mechanical behavior (conformational polymorphism and hM conformers) was incorrectly attributed to the NP monomers and could actually be either an artifact induced by the fusion polyprotein or result from the rupture of AFM-attachment (protein-substrate or protein-tip) interactions. To rule out these two possibilities, we performed two different types of negative controls. Firstly, to test the possibility that the fusion polyprotein may have induced the formation of mechanostable structures in the guest NP, we examined a non-fibrillogenic/non-neurotoxic IDP: the cytoplasmic region of VAMP2 (synaptobrevin [28]), which was fused to I27 and ubiquitin, the two carrier proteins used in our experiments. We found no conformational polymorphism, hyper-mechanostability or aggregation/fibrillogenesis (**Figs. 2b** and **S3**), indicating that conformational polymorphism and hyper-mechanostability are intrinsic to the guest NP and associated with their fibrillogenic behavior (*i.e.*, not induced by the fusion polyprotein). Secondly, to test the possibility that detachments from the AFM elements may be the source of the force peaks in the recordings, we analyzed a double mutant of A*β*42 known to strongly suppress fibrillogenesis (F19S/L34P [29]), which produced results similar to those found for VAMP2 (**Figs. 3** and **S4** and **Table 1**). It seems reasonable to assume that the F19S/L34P mutant (with an almost identical sequence to the wt protein) should have similar chemical propensities for AFM attachment as the wt A*β*42 and thus, it should display a similar source of noise if such a putative artifact was present. Hence, these results rule out the possibility that detachment from the AFM elements contaminated our data. Moreover, the absence of peaks in the F19S/L34P A*β*42 is a further proof that the A*β*42 force peaks are not originated from interactions between the NP and the carrier.

Similarly, the complete absence of force peaks in Q_19_ suggests that the force peaks of Q_35_ and Q_62_ are not originated from interactions between the NP and the carrier or from interactions with the AFM elements (all these spurious interactions would contaminate our data). The chemical propensities among these constructs are very similar in this NP model, with the only difference being the number of glutamine residues.

Finally, there is also evidence that the interactions reported here are unrelated to the carrier, as the SMFS analysis in two different carriers (I27 and ubiquitin) of VAMP2 (**Table 1**) and a prion-like protein (R. Hervás, A. Galera-Prat and M. Carrión-Vázquez, **unpublished results**) resulted in comparable results.

Thus, under our experimental conditions and using very stringent criteria for single-molecule and monomeric identification (see **Supplementary Methods**) we can readily select *bona fide* intramolecular interactions. Importantly, we also provide strong evidence that our data originate from intramolecular interactions in the NP monomer rather than intermolecular interactions (SV111 experiments with A*β*42), AFM-attachment interactions (F19S/L34P A*β*42 and Q_19_), or carrier-induced structural changes (VAMP2 and Q_19_). Indeed, we rejected many recordings where the criteria for SMFS selection were not fulfilled (see **Supplementary Methods**), some of which may well correspond to those additional undesired interactions.

**2. Supplementary discussion**

**A. On the possible existence of preferential conformations and the mechanism of conformer formation**

We further analyzed our SMFS data to extract structural information on the possible conformations adopted by the monomer. As mentioned in the main text, we first examined the possible correlation between Δ*L_c_* and mechanical stability. For all the NPs and inhibitors used here, we found a broad range of both mechanical stabilities and Δ*L_c_* values for M events (**Figs.** **2-5**). No correlation between these two parameters was observed when they were plotted against each other (**Fig. S10**), suggesting that no preferential structures are formed in the process.

In our study, we have sampled conformations of the NP monomer at 4ºC in a time window of ~30 days. In order to understand the mechanism of formation of M conformers, future studies should address the kinetics of the process. If a sequential model applies (**Fig S1**), the positive correlation between the frequency of M conformers and that of hM conformers we observed (**Table 1**) should be lost by longer incubation times since, in this model, hM conformers form at the expense of a subpopulation or the whole set of remainder M conformers (*i.e.*, the frequency of hM conformers should increase while that of their precursors should decrease).

**B. On the possible nature of the mechanical conformers**

The present findings are compatible with a recent model of potential monomeric states proposed for the four NPs studied here: disordered-denatured ensemble (NM conformers), disordered-collapsed ensemble (M conformers) and structured (hM conformers [26]).

Based on our current understanding of protein mechanostability (*i.e*., *β*-structures tend to be more mechanostable than *α*-helices or RCs [30]), on the initial disordered state of the monomeric NPs populations, as assessed by CD and NMR spectroscopies, and on the observed gain in *β*-structure along the amyloid fibrillogenic pathway (as predicted by the conventional *β*-sheet model [24,13,31]), it seems reasonable to assume that the NM class of conformers corresponds to RC conformations of NPs, while the M class corresponds to *β*-structured conformers [24,32]. There is some direct evidence of the *β*-structure composition of the toxic conformers for polyQ tracts [13]. However, it should be noted that polyproline II helices [33,34], coiled-coil [35] and collapsed structures [22] have also been implicated in amyloidogenesis. Our interpretation is supported by the current standard view of amyloidogenesis [24,25] and it has been adopted in previous SMFS studies of NPs [2,3]. As the hM conformers exhibit such extraordinary mechanostability and are likely to be on-pathway intermediates in the amyloidogenesis and/or cytotoxicity pathways, they may represent the initial *bona fide* (stable) *β*-structured conformers. This interpretation is supported by previous reports that QBP1 blocks the *β*-structure transition and neurodegeneration [13,36] and by our results with this peptide, which dramatically reduced the presence of hM conformers and a subpopulation of M conformers that could represent putative hM precursors. However, hM conformers may alternatively be by-products of any of the aforementioned pathways.

PolyQ tracts provide also an important hint on the possible interactions that maintain the M conformers. Regardless of the nature of the precise structure of these conformers, the fact that they are present in expanded polyQ tracts strongly suggests that the interactions involved are mediated by hydrogen bonding since hydrophobic or electrostatic interactions are unlikely in these polypeptides at physiological conditions.

**3. Supplementary Methods**

*Cloning*

Human *α*-synuclein (residues 1-140, UniProtKB/Swiss-Prot code **P37840) was cloned by PCR using** a complete human brain cDNA library (Clontech**) as the template**. The human A*β*42 peptide (UniProtKB/Swiss-Prot code P05067) was cloned by PCR using the pcDNA3-APP clone kindly provided by Prof. Fernando Valdivieso as the template. Sup35NM prion from the yeast *Saccharomyces cerevisiae* (residues 1-253, UniProtKB/Swiss-Prot code P05453) was cloned by PCR using the pJCSUP35 plasmid (Addgene) as the template. PolyQ tracts were cloned by PCR using plasmids containing 19, 35 and 62 Qs as templates, kindly provided by Dr. Yoshitaka Nagai [37]. The cytoplasmic region (residues 1-94) of the VAMP2 protein from *Rattus norvegicus* (UniProtKB/Swiss-Prot code P63045) was cloned by PCR using a clone (VAMP2-PGEX-KGVAMP2 [38]) containing the complete VAMP2 cDNA sequence as the template, kindly provided by Prof. Richard Scheller.

All sequences were first cloned into the pCR2.1 (Invitrogen) or pT7Blue (Novagen) vectors and after sequence verification, the QuickChange kit (Stratagene) was used for site-directed mutagenesis into *α*-synuclein (A30P and A53T) and A*β*42 (Arctic-E22G- and F19S/L34P mutants). Finally, all sequences were cloned into the pFS-2 vector using the AgeI-SmaI restriction sites of the MCS region in the carrier module [8]. The carrier module used was ubiquitin for *α*-synuclein proteins and I27 for polyQ tracts, A*β*42, and Sup35NM proteins (**Fig. 1a**). Since VAMP2 was intended to serve as a control for a non-fibrillogenic IDP, both carrier modules were used with this protein and they produced identical results (**Tables 1** and **S1**).

The I27 carrier module was designed by mutating the cDNA sequence of the I27 module (PDB code 1tit) in order to insert the AgeI and SmaI restriction sites (a short MCS) between residues A42 and A43 of the CD loop of I27 (previously shown to be a tolerant loop; **H. Li & J.M. Fernández, personal communication**), which were subsequently used to clone in all the guest proteins. For the I27+MCS carrier protein alone (without a nested protein), the CATC nucleotide sequence was deleted to maintain the open reading frame after insertion of the short MCS (the sequence is referred to as I27-MCS_Δ4_, **Table S4**). This deletion was not necessary to maintain the open reading frame in the remaining carrier-guest proteins based on the I27 carrier module.

The design of the ubiquitin carrier module is detailed elsewhere [8]. In this case, two glycine codons were added to flank the nested sequence. Thus, using the aforementioned sites, the amino acids GGTG and PGTRGG from the MCS were added to the ubiquitin carrier on each side of the nested proteins (*α*-synucleins and VAMP2). For the I27 carrier module, the amino acids TG and PG were added to each side of the nested proteins (polyQ tracts*, *A*β*42, Sup35NM and VAMP2 constructs).

In order to simplify the experiments with NPs, the structural and thermal-stability measurements (NMR, DSC and CD), as well as the aggregation/fibrillogenesis studies (Turbidity test, Congo Red assay, TEM & AFM imaging, and non-denaturing PAGE) were performed using fusion proteins that consisted of only the carrier module with the nested NP (carrier-guest proteins), excluding the marker repeats from the pFS-2 polyprotein (used to help single-molecule identification). For comparison, the isolated NPs were also used as controls in the case of *α*-synuclein and Sup35NM. The cloned sequences were: human ubiquitin (ubi, UniProtKB/Swiss-Prot **code P0CG47**), ubi-MCS, *α*-synuclein, A30P *α*-synuclein, A53T *α*-synuclein, ubi+*α*-synuclein, ubi+A30P *α*-synuclein and ubi+A53T *α*-synuclein, cloned into the pET28a vector (Novagen) using the NheI and SalI restriction sites; human I27 (UniProtKB/Swiss-Prot code Q8WZ42), I27-MCS_Δ4_, I27+A*β*42, I27+Arc A*β*42, I27+F19S/L34P A*β*42, I27+Q_19_, I27+Q_35_, I27+Q_62_, Sup35NM, I27+Sup35NM, VAMP2 and I27+VAMP2, cloned into the pET28a vector (Novagen) using the NheI and XhoI restriction sites, while ubi+VAMP2 was cloned in the same vector using NheI and SalI. With these cloning strategies, the MGSSHHHHHHSSGLVPRGSHMAS amino acid sequence remained at the N-terminus of the proteins. Two stop codons were introduced just before the restriction site located at the 3’ end of the coding region of all the sequences cloned. In both the pFS-2 and pET28a plasmids, the polyQ tracts were flanked by the MVSTHHHHHHQQ and HHGNSGPP amino acid sequences at their N- and C-terminus, respectively, which belong to the original plasmids used as templates [37]. The corresponding individual proteins for A*β*42 and polyQs were not generated by this methodology because of the difficulties inherent to the production and analyses of these small peptides.

To construct the DNA that encodes for proteins (I27)_4_-Q_20/36/51_-(I27)_3_, we used 5´-phosphorylated double-stranded oligonucleotides containing different numbers of glutamine codons (CAG and CAA). These duplex oligonucleotides contained blunt or cohesive ends, enabling direct cloning into a desired vector digested with the appropriate restriction enzymes. The oligonucleotide containing Q_20_ was cloned into the NheI-XbaI sites of the pAFM vector (kindly provided by Dr. Jane Clarke and consisting of the pRSETA vector from Invitrogen carrying eight copies of the I27 module [9] to produce the (I27)_4_-Q_20_-(I27)_3_ sequence. To construct the (I27)_4_-Q_36/51_-(I27)_3_ clones, we first cloned an oligonucleotide encoding Q_30_ into the pRSETA vector using the NheI and PvuII restriction sites. PvuII leaves a blunt-ended sequence with a terminal CAG codon, which allowed us to introduce the remaining Q_6_ and Q_21_ *via* PvuII-XbaI in a second step. Finally, we cloned the whole fragment containing Q_36_ and Q_51_ into the pAFM vector *via* the NheI-XbaI for SMFS analysis. In order to enhance the solubility of the fusion NPs, the oligonucleotides containing the polyQ regions were also flanked by two lysine residues at the N- and C-termini of their coding regions [16] flanked in turn by two amino acids from the restriction sites.

*Escherichia coli* strains DH5*α* (Invitrogen) and XL1-Blue (Stratagene) were used for all cloning/mutagenesis steps. The oligonucleotides used for all the cloning procedures are summarized in **Table S4**. All the sequences were verified by sequencing both strands of the DNA.

*Expression and purification*

pFS-2 fusion proteins were expressed in the *E. coli* C41(DE3) strain, while the isolated proteins were expressed in BL21(DE3) (Invitrogen) or C41(DE3) strains [39]. The (I27)_4_-Q_20/36/51_-(I27)_3_ proteins were expressed in the BLR(DE3)/pLysS strain (Novagen). Cultures were grown at 37ºC until they reached an OD_595_ of 0.5-0.8, and expression was induced by addition of 1mM IPTG for 4 hours.

The bacteria cells were then lysed with 1 mg/ml lysozyme, 1% Triton X-100, as reported previously [40] for *α*-synuclein proteins. For the rest of the proteins the protocol included also the addition of 0.5% Tween-20 and sonication pulses. The recombinant proteins were purified by Ni^2+^-affinity chromatography using Histrap HP FPLC columns (GE Healthcare) on an FPLC apparatus (ÄKTA Purifier, GE Healthcare), with a buffer consisting of 50 mM sodium phosphate buffer/500 mM NaCl [pH 7.4] and with 50 mM and 500 mM imidazole for binding and elution, respectively. After this first step of Ni^2+^-affinity purification, which was common for all the proteins, additional purification steps were performed when necessary (as follows):

For pFS-2+*α*-synuclein proteins, the Ni^2+^-affinity purified fractions were concentrated and the buffer exchanged to PBS by ultrafiltration using Amicon 10K filters (Millipore). After a brief sonication pulse, the samples were purified again by Strep-tag affinity chromatography using Streptrap HP FPLC columns (GE Healthcare), with PBS and PBS/2.5 mM desthiobiotin [pH 7.4] as the binding and elution buffers, respectively. For the pFS-2+A*β*42, pFS-2+Sup35NM, pFS-2+polyQs and pFS-2+VAMP2 proteins, size-exclusion chromatography was carried out in the presence of 100 mM TrisHCl/1.25-1.5 M guanidinium chloride (GdmCl) [pH 7.5]. This concentration does not denature ubiquitin or the I27 domain, allowing contaminants that previously co-eluted with the recombinant protein to be removed on a HiLoad 16/60 200 PG column (GE Healthcare [41,42]). For all proteins, ~90% purity at the end of the purification procedures was reached, as estimated by SDS-PAGE electrophoresis (**not shown**). Finally, the purified fractions were again concentrated (up to 2-3 *μ*M) and the buffer exchanged to 10 mM TrisHCl/5 mM DTT [pH 7.5] or PBS [pH 7.4] by ultrafiltration using Amicon 10K filters (Millipore) prior to performing the SMFS experiments. In the case of the (I27)_4_-Q_20/36/51_-(I27)_3_ proteins, only one affinity purification step was carried out (similar to that described above) and the proteins were left at 5-7 *μ*M in PBS /0.2 mM EDTA/1 mM DTT [pH 7.4] for SMFS analysis.

For the isolated proteins, size-exclusion chromatography was performed after the Ni^2+^-affinity chromatography purification, using 100 mM TrisHCl/1.25-1.5 M GdmCl buffer [pH 7.5]. All proteins were >90% pure when analyzed by SDS-PAGE (**not shown**). PAGE in non-denaturing conditions [43] showed a single band (**not shown**). The purified fractions were again concentrated and the buffer exchanged, by ultrafiltration with an Amicon 3K (Millipore), to the most convenient one for later applications.

In all cases (polyproteins and isolated proteins), the concentration of the protein was determined by absorbance at 280 nm using its calculated molar extinction coefficient.

*SMFS*

For SMFS experiments, a drop of the protein preparation (~2-8 *μ*l of a solution at 2-3 *μ*M) was applied on top of a drop of the corresponding experimental buffer (10 mM TrisHCl [pH 7.5] or PBS [pH 7.4]) placed onto the substrate, gold-coated or nitrilotriacetic acid (NTA)-Ni^2+^ functionalized coverslips, and allowed to adsorb for 10-20 min. Both substrates used in this study rendered identical results.

*Gold-coated coverslips*

Both pAFM and pFS-2 polyproteins can bind covalently to gold-coated substrates due to the two cysteine residues at their C-terminus [8,9]. Gold-coated glass coverslips were purchased from Arrandee (Gold arrandee™).

*Functionalization of coverslips by NTA-Ni^2+^*

NTA-Ni^2+^ functionalized coverslips can also be used to attach pFS-2 polyproteins due to the His-tag present at their N-terminus [8]. The coverslips were first immersed overnight in a 20 N KOH solution and then placed under a flow of MilliQ water for 1 hr before they were finally incubated in a solution of 2% 3-mercaptopropyl trimethoxysilane (MPTS; Sigma-Aldrich)/0.02% acetic acid at 90ºC for 1 hr. Subsequently, the coverslips were washed in a MilliQ water flow for 1 hr and then cured for 15 min in an oven at 120ºC. After cooling at room temperature for about 10 min the coverslips were transferred to a 100 mM DTT solution for 15 min and then washed under a MilliQ water flow for 1 hr. A drop of ~50 *μ*l of a solution of 20 mg/ml maleimide-C3-NTA (Dojindo Laboratories) dissolved in 10 mM 3-(N-morpholino) propanesulfonic acid (MOPS) [pH 7.0] was then deposited on each coverslip for 30 min. After rinsing in MilliQ water, a drop (60 *μ*l) of 10 mM NiCl_2_ was added to each coverslip and incubated for 10 min. Finally, the coverslips were washed briefly in MilliQ water before storage.

*SMFS experiments*

All protein data were collected within 20-30 days of protein purification. Samples were kept at 4ºC between sessions. For experiments in the presence of the inhibitory peptides QBP1 [44] (20 *μ*M of the peptide dissolved in DMSO, **Figs. 2-5**) and SV111 [27] (100 *μ*M dissolved in PBS [pH 7.4] **Fig. 3**), the samples were incubated overnight at 4ºC. The IC_50_ of the QBP1 is 3.6 *μ*M and its relative inhibitory activity at 20 *μ*M is 85.6 % (at a protein concentration of 7.5 *μ*M [44]). Polyproteins were used at ~2 *μ*M (a stoichiometric inhibitor/protein ratio ~4 fold higher than that used elsewhere [44]). For SV111 the IC_50_ is 32 ± 1 *μ*M (at a concentration of A*β*42 of 25 *μ*M [27]) and the inhibitor/protein stoichiometric ratio used was ~30 fold higher than that used previously [27].

QBP1-M8 (sequence: WKWWPGIF) was synthesized at the Proteomic Facility of the CBMSO/CSIC-UAM using solid-state Fmoc chemistry with acetylation of the N-terminal amine group. HPLC analysis revealed a purity >95.6%. SV111 (sequence: GDKAGAEVLAAVKAIKEK) was purchased from GenScript USA, Inc. and it was >95.3% pure as determined by HPLC. The identity of SV111 was confirmed by mass spectrometry and NMR spectroscopy. Our custom-made single-molecule AFM, with added imaging capabilities and its mode of operation have been described previously [45]. Before each experiment, the cantilever tip was cleaned for 1 min with a UV lamp (UV/Ozone ProCleaner^TM^ Plus, Bioforce Nanosciences Inc.). The spring constant of each individual Si_3_N_4_ cantilever (MLCT-AUNM, Veeco Metrology Group; and Biolever, Olympus) was calculated using the equipartition theorem [46], resulting in values ranging from 35 to 70 pN/nm for the MLCT-AUNM and ~30 pN/nm for the Biolever cantilevers. Proteins were picked up by adsorption to the cantilever tip by approaching the piezoelectric device in *z* axis, which was then retracted for several hundred nm to stretch the adsorbed proteins. All experiments were performed at a constant pulling speed of 0.4 nm/ms in the so-called length-clamp mode of SMFS [30] and all the data were analyzed using Igor Pro 6 (Wavemetrics). The elasticity of the stretched proteins was analyzed using the WLC model of polymer elasticity [47,48]:

$F(x)=\frac{k_{B}T}{p}\left[\frac{1}{4\left(1-\frac{x}{L_{C}}\right)^{2}}-\frac{1}{4}+\frac{x}{L_{C}}\right]$*,*

where *F* is the force, *p* is the persistence length, *x* is the end-to-end length, and *L_c_* is the contour length of the stretched protein. *L_c_* and *p* are the adjustable parameters.

*Analysis of SMFS data*

The criteria used for selecting *bona fide* single-molecule recordings were quite stringent and can be summarized as follows: a) the inclusion of the N2B disordered polypeptide in the pFS-2 vector acts as a spacer to overcome the noisy proximal region of the force-extension spectra (50-70 nm if the polyprotein has been pulled from its termini, [8]). Thus, we rejected any recording whose putative NP force peak appeared in this region [2]; b) the recording should show several force peaks attributable to the unfolding of the ubiquitin repeats present in the pFS-2 vector as markers, the *F_u_* and Δ*L_c_* of which are characteristic and well described in the literature [49]; c) the polyprotein should not show more force peaks than those expected from the design of the pFS-2 protein (excluding those derived from the unfolding of the NPs due to their mechanical plasticity); d) the total length of the unfolded molecule should not be larger than the total length of the extended polypeptide (taking into the account the number of ubiquitin marker units unfolded and considering a gain in length of 0.4 nm *per* unfolded amino acid [50]); e) since the NP is “force hidden” inside the carrier module of the pFS-2 vector in the carrier-guest strategy, the force peak originating from the unfolding of the carrier module should always precede the force peaks corresponding to the unfolding of the grafted NP (**Fig. 1b**); f) any force peak that appeared at an extension shorter than that corresponding to the complete unfolding of the carrier module (26 nm for the carrier ubiquitin and 29.5 nm for the carrier I27, [8] was excluded from our analyses as, in principle, it may have originated from spurious interactions between the nested NP and the carrier; g) only force data that showed a Δ*L_c_* that, when summed, coincided exactly with the Δ*L_c_* of the NP (within a tolerance range of ± 1 nm) were included in our analyses (“b” and “c” in **Fig. 1b**). In the case of NM events the tolerance range was less stringent (± 2 nm), as these are more straightforward events to identify. To obtain about 100 molecules that fulfilled these criteria the number of required attempts was in the range of 50,000-100,000. In the case of (I27)_4_-Q_20/36/51_-(I27)_3_ proteins, based on the arrangement of modules in the polyprotein, the criterion used was the presence of at least 5 I27 force peaks, the minimum requirement to be completely sure that the region of interest (Q_20/36/51_) has been stretched (**Fig. S8a, b**).

The number of molecules (or conformers) in each group (NM, M and hM) shown in **Table 1** is independent of the number of force peaks observed *per* molecule. For a molecule to be considered mechanostable, it must exhibit at least one force peak. hM conformers are those mechanostable conformers with at least one peak with ≥400 pN. The error associated with the frequency of these events (**Tables 1** and **S3**) was obtained by assuming a 10 pN error (average peak-to-peak distance in the force trace) in the SMFS measurement of the 400 pN arbitrary limit, and subtracting or adding the events that were affected by the application of this range in the calculations.

*AFM imaging*

The formation of amyloid fibers in I27+Sup35NM was assessed by incubating a 12.5 *μ*M sample in 10 mM TrisHCl/5 mM DTT [pH 7.5] buffer for 30 days at 37ºC with no stirring (in the presence of 0.02% NaN_3_, used as a bacteriostatic agent). For AFM image acquisition, we used a 1:10 dilution of the protein and 1 mM CaCl_2_ was added to the experimental buffer to facilitate the adsorption of fibers to the mica substrate. A drop of the diluted protein was deposited on freshly exfoliated mica for 15 minutes and images were obtained using MLCT-AUNM cantilevers (Veeco Metrology Group) in the dynamic mode in the aforementioned buffer [51]. The fibers observed were several hundred nm long and ~15 nm high (**Figs. S6d** and **Table S1**).

To form (I27)_4_-Q_51_-(I27)_3_ amyloid fibers, a protein sample at a concentration of 12.8 *μ*M was incubated for 30 days at 37ºC with no stirring (in the presence of 0.02% NaN_3_). For AFM image acquisition, a 1:25 dilution of the protein in PBS buffer [pH 7.4] was used. Images were obtained on a mica substrate in PBS [pH 7.4], using both the aforementioned cantilevers and the dynamic mode [51] (**Fig. S8d**).

The AFM image of ubi+A53T *α*-synuclein fibers (**Fig. S5d** and **Table S1**) was obtained by incubating the sample (0.3 mM in PBS [pH 7.4] + 0.02% NaN_3_) for 51 days at 37ºC without stirring. We first obtained control images of recently exfoliated mica in the presence of filtered PBS (using MLCT-AUNM cantilevers from Veeco Metrology Group). After confirming that the free surface was adequately clean and flat for imaging, a 20 *μ*l aliquot of the sample was taken (following gentle resuspension of the precipitate), deposited on dry mica and left to adsorb for 15 min. The drop was dried by applying a very mild flow of N_2_ and then rehydrated with the PBS buffer present in the fluid cell of the AFM. This procedure is identical to that used previously for AFM imaging of *α*-synuclein amyloid fibers [52]. Images were acquired in the dynamic mode.

*CD of monomers*

**The far-UV CD spectra of the proteins (at** concentrations of 5-20 *μ*M) were collected at 20ºC in 10 mM KH_2_PO_4_ [pH 4.7] in a JASCO-J810 spectropolarimeter (JASCO Inc.) equipped with a Peltier temperature control unit and using quartz cuvettes of 1 mm pathlength, as described previously [53]. The buffer contribution was subtracted from the experimental data and corrected spectra were converted into molar ellipticity ([*θ*]) using the average molecular masses *per* residue with Spectra Analysis software (Jasco Inc.). After recording the first protein spectrum with samples kept at 4ºC (day 0), the protein was incubated at 37ºC without stirring (in the presence of 0.02% NaN_3_), and additional spectra were collected on days 2, 5 and 7 in order to monitor the structural changes experienced by the NPs and the VAMP2 control protein. Furthermore, NPs (**Figs. S2a** and **S4a-S6a**, left panel) and VAMP2 (**Fig. S3a,** left panel) were examined by CD upon addition of 20 *μ*M QBP1 or 100 *μ*M SV111. The final concentration of DMSO in the QBP1 samples was below 0.01% to avoid DMSO interference with the ellipticity (*θ*) measurements. Finally, *α*-synuclein, alone and inserted in ubiquitin carrier, was measured in the presence of 1mM sodium dodecyl sulfate (SDS). The secondary structure content of each protein and its evolution during incubation were estimated by spectra deconvolution using the CDNN analysis program [54] (**Figs. S2a-S6a**, right panel). The weighted contribution of the carrier module was subtracted to estimate the secondary structure content of the NPs in the carrier-guest proteins.

For thermal stability measurements (**Fig. S9a**), the ellipticity changes induced by increasing the temperature (at a constant heating rate of 60ºC/hr) were measured at 224 nm in 100 mM sodium phosphate buffer/100 mM NaCl [pH 7.4] using protein concentrations of 12.5-50 *μ*M. The reversibility of the denaturation process was determined by comparing the spectra of the native proteins (before their thermally-induced denaturation) with those obtained after rapidly re-cooling the heated samples. The unfolding process of all tested samples was reversible, with the exception of the I27-MCS; in this case the proline residue inserted in the loop may interfere with protein folding [55].

*DSC of monomers*

DSC experiments were carried out in a VP-DSC microcalorimeter (MicroCal, LLC) as described previously [45]. Typically, samples were scanned from 10 ºC to 115 ºC, at a heating rate of 50 ºC/hr, and the protein concentrations ranged from 35 to 99 *μ*M in 10 mM glycine/acetic acid buffer [pH 3.0] (**Fig. S9b**). Data were collected and analyzed with the DSC-Origin software as described elsewhere [56]. The reversibility of the thermal transitions, as assessed by re-scanning the previously heated samples, decreased upon heating to temperatures much higher than the thermal transition range. Variation of the heating rate did not significantly modify the experimental curves, indicating a weaker influence of irreversibility within the temperature interval where protein denaturation occurred.

DSC measurements were only performed for ubiquitin-based constructs (*α*-synucleins) due to the high thermal stability of ubiquitin [57], which made them unsuitable for thermal stability measurements by CD.

*^1^H NMR of monomers*

In order to identify possible interactions between the carrier modules and the nested NPs that may induce structural changes in the latter, we performed monodimensional NMR experiments on all the monomeric proteins (**Figs. S2b-S6b**) and also ^1^H 2D NOESY spectra on some of the less stable carrier-guests polypeptides; namely, I27+VAMP2, I27+Sup35NM and ubi+A53T *α*-syn (**data not shown**). Accordingly, concentrated samples (0.1-1 mM) were dialysed against 10 mM KH_2_PO_4_ [pH 4.6-4.7] with 10% D_2_O. Measurements were taken at 25ºC using a Bruker AV 800 spectrometer (Bruker BioSpin) equipped with a cryoprobe with a *z*-gradient. The temperature was calibrated using an ethanol sample and the signal of the trimethyl moiety of sodium 2,2-dimethyl-2-silapentane-5-sulfonate [(CH_3_)_3_-Si-CH_2_-CH_2_-CH_2_-SO_3_^-^ Na^+^] (DSS) was used as an internal reference of the chemical shift [58]. Selective pre-saturation or a WATERGATE module [59] were used to reduce the signal of the water present in the sample. The mixing time for the 2D NOESY experiments was 100 ms. The spectra were analyzed using TopSpin 2.0 (Bruker BioSpin). The assigned signals in the ubiquitin and I27 spectra (**Figs. S2b-S6b**, right) were based on previously reported data [60,61].

*TEM imaging*

Monomer samples (in the presence or absence of 20 *μ*M QBP1 or 100 *μ*M SV111, as indicated) were incubated for 20-30 days at 37ºC at a concentration of 10-40 *μ*M in 10 mM TrisHCl [pH 7.5] in the presence of 0.02% NaN_3_ and with no stirring.

A*β*42 peptide was purchased to Shangai RD Biosciences LTD. For fibrillogenesis assays, the starting monomers were first prepared by dissolving 4.0 mg A*β*42 in 800 *µ*L hexafluoro-2-propanol (HFIP) for 10-20 min at RT. The resulting solution was aliquoted into siliconized eppendorf tubes (70 *µ*L each one), which were subjected to a gentle stream of N_2_ for 5-10 min to evaporate the HFIP. Samples were stored at -80ºC until use. To prepare fibers, each aliquot was reconstituted in 77.5 *µ*L DMSO using pipette mixing (instead of vortexing). After 3-5 min, 1.21 *m*L H_2_O were added leading to a final concentration of 60 *µ*M. Finally, the addition of QBP1 lead to an A*β*42 concentration of 50 *µ*M. QBP1 was assayed at the following concentrations: 0, 100, 200, 500 and 1000 *µ*M. TEM images were taken (**Fig. S4d,** panels **6** and **7**) after 13 days of incubation at 37ºC without stirring.

In order to test the ability to form amyloid fibers at the temperature at which the proteins were stored prior to SMFS experiments (4ºC), Sup35NM, I27+Sup35NM and pFS-2+Sup35NM proteins (20 *μ*M) were incubated at 4ºC in the aforementioned buffer for 29 days, while the (I27)_4_-Q_51_-(I27)_3_ protein was incubated at 37ºC (also without stirring) at a concentration of 10 *μ*M in PBS [pH 7.4] + 0.02% NaN_3_ for 28 days.

Subsequently, 10 *μ*l of several sample dilutions (0.45-4.5 *μ*M) was adsorbed onto carbon-coated 300-mesh copper grids (Ted Pella) and negatively stained for 30 s using 1-2% uranyl acetate (EMS; previously filtered to prevent aggregate formation), as described previously [62]. Immediately before use, the carbon-coated grids were *glow-discharged* to enhance their hydrophilicity using an Emitech K100X apparatus (Quorum Technologies). The formation of amyloid fibers (**Figs. 2-5, S2d-S6d** and **Table S1**) was analyzed on a JEOL 1200EX II (Jeol Limited) electron microscope equipped with a CCD Megaview III camera (Olympus Soft Imaging). The fibers were several hundred nanometers long and 20-23 nm wide. The images were acquired at a magnification of 30,000-150,000x and a voltage of 80 kV. The same protocol was used to obtain images for the polyprotein samples (I27)_4_-Q_51_-(I27)_3_ (**Fig. S8d**) and pFS-2+Sup35NM (**Fig. S6d,** panel **8**).

*Turbidmetry and Congo Red binding assays*

To measure turbidity (**Figs. S2c-S6c, S8c** and **Table S1**), monomeric protein samples (at 12.5-50 *μ*M in 10 mM TrisHCl [pH 7.5] with 0.02% NaN_3_ added) and (I27)_4_-Q_20/36/51_-(I27)_3_ proteins (at 10 *μ*M in PBS/0.2 mM EDTA/1 mM DTT [pH 7.4] with 0.02 % NaN_3_ added) were incubated at 37ºC without stirring in the presence or absence of 20 *μ*M QBP1 or 100 *μ*M SV111. The aggregation process of the samples was monitored indirectly by measuring their turbidity, based on the absorbance at 405 nm using a UV-visible spectrophotometer (Nanodrop, Thermo Scientific), as described previously [37].

Congo Red assays (**Fig. S7** and **Table S1**) were performed on monomer samples (10 *μ*M in the 10 mM TrisHCl buffer [pH 7.5]) incubated at 37ºC without stirring in the presence of 0.02% NaN_3_. After an appropriate incubation period (30 days), the protein solutions were mixed with a 30 *μ*M Congo Red solution (dissolved in 5 mM sodium phosphate buffer/300 mM NaCl [pH 7.5], and filtered through a 0.22 *μ*m filter to remove the Congo Red micelles formed) and incubated at room temperature for 30 min. The UV-visible spectra of these mixtures were measured in a UV-visible spectrophotometer (Nanodrop, Thermo Scientific). We observed the typical bathochromic and hyperchromic shifts in the samples that contained amyloid aggregates. The concentration of Congo Red bound to the formed amyloid aggregates was determined using the following equation: Congo Red (*μ*mol/l) = A_540_/25,295 – A_480_/46,306 [29] Congo Red bound to amyloid aggregates also exhibited typical fluorescence, which was monitored with a Leica DMI6000B inverted optical microscope (Leica Microsystems), and green birefringence, monitored under a polarized light microscopy (**not shown**).

***Order/disorder predictions***

Disorder predictions for all the proteins in this study were made using Predictor Of Naturally Disordered Regions (PONDR, Molecular Kinetics) VLXT and VL3. These predictors classify each residue within a sequence as either ordered or disordered. PONDR VL-XT integrates three feed-forward neural networks: VL1 predictor [63], which predicts non-terminal residues, and XT [64], with two predictors that include the N-and C- terminal residues. PONDR VL3 is a feed-forward neural network that combines the predictions of 30 neural networks for the entire protein sequence [65]. As stated in **Fig. S13**, since PONDR VLXT and VL3 use different parameters and window size, and they are trained on very different datasets, it is expected that their outputs will differ significantly. PONDR VL3 is one of the most accurate predictors of long disordered regions in proteins and due to the longer averaging window, it provides relatively smooth curves. PONDR VLXT is very sensitive to sequence details and it is frequently used to identify sequence features potentially associated with protein function.

**4. REFERENCES TO SUPPLEMENTARY METHODS**

1. Dougan L, Li J, Badilla CL, Berne BJ, Fernández JM (2009) Single homopolypeptide chains collapse into mechanically rigid conformations. Proc Natl Acad Sci USA 106: 12605-12610.

2. Sandal M, Valle F, Tessari I, Mammi S, Bergantino E et al. (2008) Conformational equilibria in monomeric α-synuclein at the single-molecule level. PLoS Biol 6: 99-108.

3. Brucale M, Sandal M, Di Maio S, Rampioni A, Tessari I et al. (2009) Pathogenic mutations shift the equilibria of α-synuclein single molecules towards structured conformers. Chembiochem 10: 176-183.

4. Wegmann S, Schoeler J, Bippes CA, Mandelkow E, Muller DJ (2011) Competing interactions stabilize pro- and anti-aggregant conformations of human tau. J Biol Chem 286: 20512-20524.

5. Carrión-Vázquez M, Oberhauser AF, Fisher TE, Marszalek PE, Li H et al. (2000) Mechanical design of proteins studied by single-molecule force spectroscopy and protein engineering. Prog Biophys Mol Biol 74: 63-91.

6. Li H, Oberhauser AF, Redick SD, Carrión-Vázquez M, Erickson HP et al. (2001) [Multiple conformations of PEVK proteins detected by single-molecule techniques.](http://www.ncbi.nlm.nih.gov/pubmed/11526214) Proc Natl Acad Sci USA 98: 10682-10686.

7. Li H, Linke WA, Oberhauser AF, Carrión-Vázquez M, Kerkvliet JG et al. (2002) [Reverse engineering of the giant muscle protein titin.](http://www.ncbi.nlm.nih.gov/pubmed/12198551) Nature 418: 998-1002.

8. Oroz J, Hervás R, Carrión-Vázquez M (2012) Unequivocal single-molecule identification in AFM-based force spectroscopy with pFS vectors. Biophys J 102: 682-690.

9. Steward A, Toca-Herrera JL, Clarke J (2002) Versatile cloning system for construction of multimeric proteins for use in atomic force microscopy. Prot Sci 11: 2179-2183.

10. [Sambashivan S](http://www.ncbi.nlm.nih.gov/pubmed?term="Sambashivan S"%5BAuthor%5D), Liu Y, [Sawaya MR](http://www.ncbi.nlm.nih.gov/pubmed?term="Sawaya MR"%5BAuthor%5D), [Gingery M](http://www.ncbi.nlm.nih.gov/pubmed?term="Gingery M"%5BAuthor%5D), [Eisenberg D](http://www.ncbi.nlm.nih.gov/pubmed?term="Eisenberg D"%5BAuthor%5D) (2005) Amyloid-like fibrils of ribonuclease A with three-dimensional domain-swapped and native-like structure. Nature 437: 266-269.

11. Teng PK, Eisenberg D (2009) Short protein segments can drive a non-fibrillizing protein into the amyloid state. Protein Eng Des Sel 22: 531-536.

12. Zhao M, Cascio D, Sawaya MR, Eisenberg D (2011) Structures of segments of α-synuclein fused to maltose-binding protein suggest intermediate states during amyloid formation. Protein Sci 20: 996-1004.

13. Nagai Y, Inui T, Popiel HA, Fujikake N, Hasegawa K et al. (2007) A toxic monomeric conformer of the polyglutamine protein. Nat Struct Biol 14: 332-340.

14. Wetzel R (2010) Misfolding and aggregation in huntington disease and other expanded polyglutamine repeat diseases. In: Ramírez-Alvarado M, Kelly JW, Dobson CM, editors. Protein Misfolding Diseases: Current and Emerging Principles and Therapies. John Wiley & Sons, pp. 305-324.

15. Perutz MF, Johnson T, Suzuki M, Finch JT (1994) Glutamine repeats as polar zippers: their possible role in inherited neurodegenerative diseases. Proc Natl Acad Sci U S A 91: 5355-5358.

16. Venkatraman P, Wetzel R, Tanaka M, Nukina N. & Goldberg AL (2004) Eukaryotic proteasomes cannot digest polyglutamine sequences and release them during degradation of polyglutamine-containing proteins. Mol Cell 14: 95-104.

17. Tanaka M, Morishima I, Akagi T, Hashikawa T, Nukina N (2001) Intra- and intermolecular β-pleated sheet formation in glutamine-repeat inserted myoglobin as a model for polyglutamine diseases. J Biol Chem 276: 45470-45475.

# 18. Wang L, Rivera EV, Benavides-Garcia MG, Nall BT (2005) Loop entropy and cytochrome *c* stability J Mol Biol 353: 719-729.

19. Ulmer TS, Bax A, Cole NB, Nussbaum RL (2005) Structure and dynamics of micelle-bound human α-synuclein. J Biol Chem 280: 9595-9603.

20. Kayed R, Head E, Thompson JL, McIntire TM, Milton SC et al. (2003) [Common structure of soluble amyloid oligomers implies common mechanism of pathogenesis.](http://www.ncbi.nlm.nih.gov/pubmed/12702875) Science 300: 486-489.

21. Pace CN, Grimsley GR, Thomson JA, Barnett BJ (1998) Conformational stability and activity of ribonuclease T_1_ with zero, one and two intact disulfide bonds. J Mol Biol 263: 11820-11825.

22. Vitalis A, Wang X, Pappu, RV (2008) Atomistic simulations of the effects of polyglutamine chain length and solvent quality on conformational equilibria and spontaneous homodimerization. J Mol Biol 384: 279-297.

23. Ganchev DN, Cobb NJ, Surewi K, Surewicz WK (2008) Nanomechanical properties of human prion protein amyloid as probed by force spectroscopy. Biophys J 95: 2909-2915.

24. Chiti F, Dobson CM (2006) Protein misfolding, functional amyloid and human disease. Ann Rev Biochem 75: 333-366.

25. Uversky V (2009) Intrinsic Disorder in Proteins Associated with Neurodegenerative Diseases. In: Ovádi J, Orosz F, editors. Protein Folding and Misfolding: Neurodegenerative Diseases, Springer-Verlag. pp. 21-75.

26. Frieden C (2007) Protein aggregation processes: In search of the mechanism. Protein Sci 16: 2334-2344.

27. Arslan PE, Mulligan VK, Ho S, Chakrabartty A (2010) Conversion of Aβ42 into a folded soluble native-like protein using a semi-random library of amphipathic helices. J Mol Biol 396: 1284-1294.

28. Hazzard J, Südhof TC, Rizo J (1999) NMR analysis of the structure of synaptobrevin and of its interaction with syntaxin. J Biomol NMR 14: 203-207.

29. Wurth C, Guimard NK, Hecht MH (2002) Mutations that reduce aggregation of the Alzheimer’s Aβ42 peptide: an unbiased search for the sequence determinants of amyloidogenesis. J Mol Biol 319: 1279-1290.

30. Carrión-Vázquez M, Oberhauser AF, Díez H, Hervás R, Oroz J et al. (2006) Protein nanomechanics –as Studied by AFM Single-Molecule Force Spectroscopy. In: Arrondo JLR, Alonso A, editors. Advanced Techniques in Biophysics. Springer-Verlag. pp.163-245.

# 31. Uversky VN, Dunker AK (2010) Understanding protein non-folding. **Biochem Biophys Acta 1804: 1231-1264.**

32. Nagai Y, Popiel HA (2008) Conformational changes and aggregation of expanded polyglutamine proteins as therapeutic targets of the polyglutamine diseases: exposed β-sheet hypothesis. Curr Pharm Des 14: 3267-3279.

33. Syme CD, Blanch EW, Holt C, Jakes R, Goedert M et al. (2002) A Raman optical activity study of rheomorphism in caseins, synucleins and tau. New insight into the structure and behaviour of natively unfolded proteins. Eur J Biochem 269: 148-156.

34. Uversky VN, Fink AL (2004) Conformational constraints for amyloid fibrillation: the importance of being unfolded. Biochem Biophys Acta 1698: 131-153.

35. Fiumara F, Fioriti L, Kandel ER, Hendrickson WA (2010) Essential role of coiled coils for aggregation and activity of Q/N-rich prions and polyQ proteins. Cell 143: 1121-1135.

36. Popiel HA, Nagai Y, Fujikake N, Toda T (2007) Protein transduction domain-mediated delivery of QBP1 suppresses polyglutamine-induced neurodegeneration in vivo. Mol Ther 15: 303-309.

37. Nagai Y, Tucker T, Ren H, Kenan DJ, Henderson BS et al. (2000) Inhibition of polyglutamine protein aggregation and cell death by novel peptides identified by phage display screening. J Biol Chem 275: 10437-10442.

38. Calakos N, Bennett MK, Peterson KE, Scheller RH (1994) Protein-protein interactions contributing to the specificity of intracellular vesicular trafficking. Science 263: 1146-1149.

39. Miroux B, Walker JE (1996) Over-production of proteins in Escherichia coli: mutant hosts that allow synthesis of some membrane proteins and globular proteins at high levels. J Mol Biol 260: 289-298.

40. Sambrook J (1989) Molecular Cloning: A laboratory manual. 2nd Ed., Cold Spring Harbor. 1659 p.

41. Carrión-Vázquez M, Oberhauser AF, Fowler SB, Marszalek PE, Broedel SE et al. (1999) Mechanical and chemical unfolding of a single protein: a comparison. Proc Natl Acad Sci USA 96: 3694-3699.

42. Went HM, Benítez-Cardoza CG, Jackson SE (2004) Is an intermediate state populated on the folding pathway of ubiquitin? FEBS Lett 567: 333-338.

43. Gallagher SR (2001) One-dimensional electrophoresis using nondenaturing conditions. Curr Protoc Protein Sci. John Wiley & Sons. Chapter 10, Unit 10.3.

# 44. Tomita K, Popiel HA, Nagai Y, Toda T, Yoshimitsu Y et al. 2009) Structure-activity relationship study on polyglutamine binding peptide QBP1. Bioorg Med Chem 17: 1259-1263.

45. Valbuena A, Oroz J, Hervás R, Vera AM, Rodríguez D et al (2009) On the remarkable mechanostability of scaffoldins and the mechanical clamp motif. Proc Natl Acad Sci USA 106: 13791-13796.

46. Florin EL (1995) Sensing specific molecular interactions with the atomic force microscope. Biosens Bioelectron 10: 895-901.

47. Bustamante C, Marko JF, Siggia ED, Smith S (1994) Entropic elasticity of lambda-phage DNA. Science 265: 1599-1600.

48. Marko JF (1994) Stretching DNA. Macromolecules 28: 8759-8770.

49. Carrión-Vázquez M, Li H, Lu H, Marszalek PE, Oberhauser AF et al. (2003) The mechanical stability of ubiquitin is linkage dependent. Nat Struct Biol 10: 738-743.

50. Ainavarapu SR, Brujic J, Huang HH, Wiita AP, Lu H et al. (2007) Contour length and refolding rate of a small protein controlled by engineered disulfide bonds. Biophys J 92: 225-233.

51. Valbuena A, Oroz J, Vera AM, Gimeno A, Gómez-Herrero J et al. (2007) Quasi-simultaneous imaging/pulling analysis of single polyprotein molecules by atomic force microscopy. Rev Sci Instrum. doi:10.1063/1.2794732.

52. Conway KA, Harper JD, Lansbury PT (1998) Accelerated in vitro fibril formation by a mutant α-synuclein linked to early-onset Parkinson disease. Nat Med 4: 1318-1320.

53. Varea J, Monterroso B, Sáiz JL, López-Zumel C, García JL et al. (2004) Structural and thermodynamic characterization of Pal, a phage natural chimeric lysin active against pneumococci. J Biol Chem 279: 43697-43707.

54. Böhm G, Muhr R, Jaenicke R (1992) Quantitative analysis of protein far UV circular dichroism spectra by neural networks. Protein Eng 5: 191-195.

55. [Pradeep L](http://www.ncbi.nlm.nih.gov/pubmed?term="Pradeep L"%5BAuthor%5D), [Shin HC](http://www.ncbi.nlm.nih.gov/pubmed?term="Shin HC"%5BAuthor%5D), [Scheraga HA](http://www.ncbi.nlm.nih.gov/pubmed?term="Scheraga HA"%5BAuthor%5D) (2006) Correlation of folding kinetics with the number and isomerization states of prolines in three homologous proteins of the RNase family. [FEBS Lett](javascript:AL_get(this, 'jour', 'FEBS Lett.');) 580: 5029-5032.

56. Varea J, Saiz JL, López-Zumel C, Monterroso B, Medrano FJ et al. (2000) Do sequence repeats play an equivalent role in the choline-binding module of pneumococcal LytA amidase? J Biol Chem 275: 26842-26855.

57. [Wintrode PL](http://www.ncbi.nlm.nih.gov/pubmed?term="Wintrode PL"%5BAuthor%5D), [Makhatadze GI](http://www.ncbi.nlm.nih.gov/pubmed?term="Makhatadze GI"%5BAuthor%5D), [Privalov PL](http://www.ncbi.nlm.nih.gov/pubmed?term="Privalov PL"%5BAuthor%5D) (1994) Thermodynamics of ubiquitin unfolding. [Proteins](javascript:AL_get(this, 'jour', 'Proteins.');) 18: 246-253.

# 58. Markley JL, Bax A, Arata Y, Hilbers CW, Kaptein R et al. (1998) Recommendations for the presentation of NMR structures of proteins and nucleic acids. IUPAC-IUBMB-IUPAB inter-union task group on the standardization of data bases of protein and nucleic acid structures determined by NMR spectroscopy. J Biomol NMR 12: 1-23.

# 59. [Piotto M](http://www.ncbi.nlm.nih.gov/pubmed?term="Piotto M"%5BAuthor%5D), [Saudek V](http://www.ncbi.nlm.nih.gov/pubmed?term="Saudek V"%5BAuthor%5D), [Sklenár V](http://www.ncbi.nlm.nih.gov/pubmed?term="Sklenár V"%5BAuthor%5D) (1992) Gradient-tailored excitation for single-quantum NMR spectroscopy of aqueous solutions. [J Biomol NMR](javascript:AL_get(this, 'jour', 'J Biomol NMR.');) 2: 661-665.

60. Improta S, Politou AS, Pastore A (1996) Immunoglobulin-like modules from components of muscle elasticity. Structure 4: 323-327.

61. Di Stefano DL, Wand AJ (1987) Two-dimensional ^1^H NMR study of human ubiquitin: a main chain directed assignment and structure analysis. Biochemistry 26: 7272-7281.

# 62. Chen B, Thurber KR, Shewmaker F, Wickner RB, Tycko R (2009) Measurement of amyloid fibril mass-per-length by tilted-beam transmission electron microscopy. Proc Natl Acad Sci USA 106: 14339-14344.

# 63. Romero P, Obradovic Z, Li X, Garner EC, Brown CJ et al. (2001) Sequence complexity of disordered protein. Proteins 42: 38-48.

# 64. Li X, Romero P, Rani M, Dunker AK, Obradovic Z (1999) Predicting protein disorder for N-, C-, and internal regions. Genome Inform Ser Workshop Genome Inform 10: 30-40.

# 65. Obradovic Z, Peng K, Vucetic S, Radivojac P, Brown CJ et al. (2003) Predicting intrinsic disorder from amino acid sequence. Proteins 53, Suppl 6: 566-572.
